# Ligand Postsynthetic Functionalization with Fluorinated Boranes and Implications in Hydrogenation Catalysis

**DOI:** 10.1021/acscatal.3c02764

**Published:** 2023-11-30

**Authors:** Macarena G. Alférez, Juan J. Moreno, Miguel A. Gaona, Celia Maya, Jesús Campos

**Affiliations:** Instituto de Investigaciones Químicas (IIQ), Departamento de Química Inorgánica and Centro de Innovación en Química Avanzada (ORFEO−CINQA), Consejo Superior de Investigaciones, Científicas (CSIC) and Universidad de Sevilla, Avenida Américo Vespucio 49, 41092 Sevilla, Spain

**Keywords:** pendant borane, hydrogenation, ligand functionalization, rhodium, σ-borane complex

## Abstract

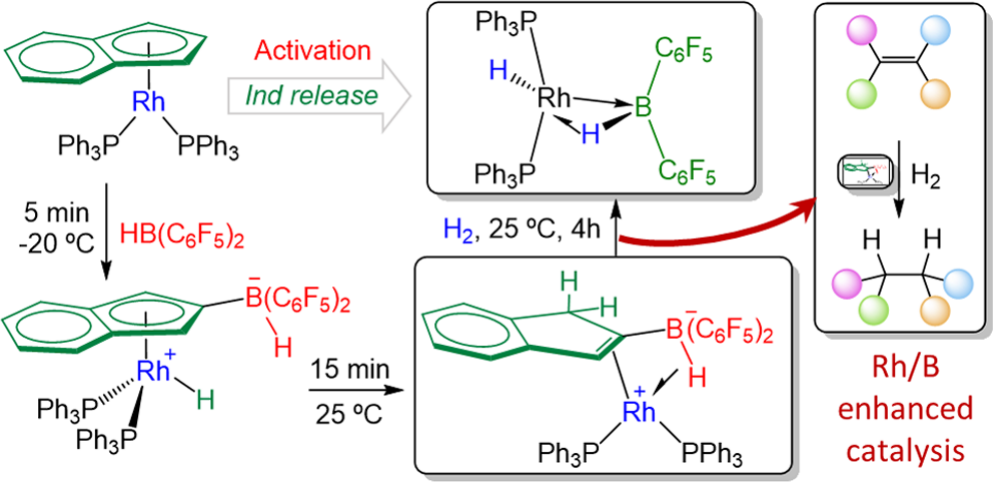

The incorporation of boron functionalities into transition-metal catalysts has become a promising strategy to improve catalytic performance, although their synthesis typically entails the preparation of sophisticated bifunctional ligands. We report here the facile and direct postsynthetic functionalization of rhodium(I) compound [(η^5^-C_9_H_7_)Rh(PPh_3_)_2_] (**1**) by treatment with perfluorinated boranes. Borane addition to **1** results in an unusual C(sp^2^)-H hydride migration from the indenyl ligand to the metal with the concomitant formation of a C–B bond. In the case of Piers’ borane [HB(C_6_F_5_)_2_], this is followed by a subsequent hydride migration that leads to an unprecedented 1,2-hydrogen shift reminiscent of Milstein’s cooperative dearomatization pathways. Computational investigations provide a mechanistic picture for the successive hydride-migration steps, which enriches the non-innocent chemistry of widespread indenyl ligands. Moreover, we demonstrate that the addition of Piers’ borane is highly beneficial for catalysis, increasing catalyst efficiency up to 3 orders of magnitude.

## Introduction

**Figure 1:**
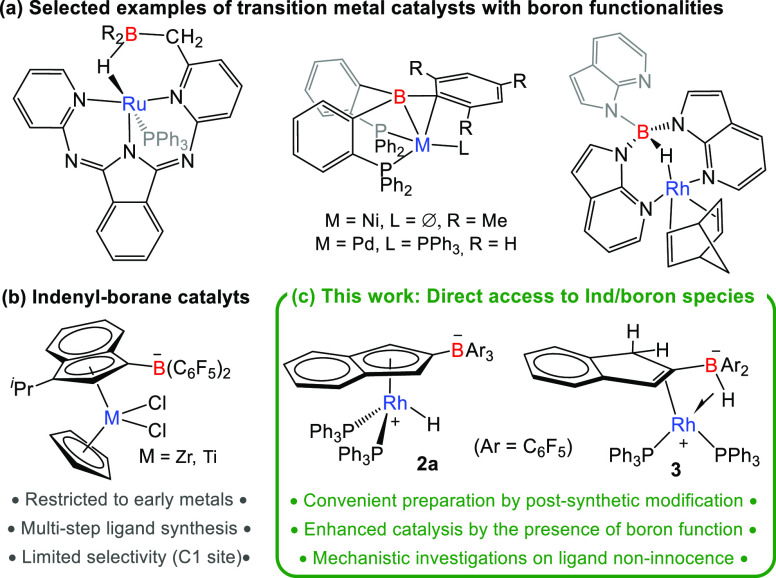
(a) Selected examples of transition-metal catalysts that contain boron functionalities; (b) representative example of polymerization catalysts based on an indenyl/boron ligand; and (c) rhodium complexes containing borate-functionalized indenyl ligands investigated in this work.

The broad concept of metal–ligand cooperation has enriched the traditional notion of an active metal site surrounded by spectator ligands.^1^ Among the wide variety of bifunctional ligands, those bearing a Lewis acidic site have enjoyed increasing popularity.^2^ Not surprisingly, ligands containing group 13 elements are the preferred choice, with boron as the more prevalent,^3,4^ including the recent development of ligands with multiple borane sites.^5^ Accordingly, catalytic applications of boron-containing transition-metal catalysts are rapidly increasing (Figure 1a). To cite some relevant examples, Peters^6^ and Owen^7^ demonstrated this positive partnership in hydrogenation reactions and Bourissou for the dehalogenation of aromatic species,^8^ while Szymczak has shown enhanced activity and selectivity during alkyne hydrogenation.^9^ Moreover, the flexibility of M–B bonds has proven crucial to accommodate challenging bond activation reactions at transition metals, as demonstrated by Peters in the context of N_2_ reduction.^10^ In addition, pendant boranes without direct M–B bonding offer great opportunities, as recently demonstrated by Werlé on the chemoselective reduction of nitroarenes.^11^

Borane containing ligands are usually tethered to the metal by two or more supporting groups to provide sufficient stability.^3^ While phosphine- and nitrogen-based donors have been amply used, the functionalization of widespread indenyl ligands with boron fragments remains comparatively underdeveloped. More precisely, they have been investigated in the narrow context of olefin polymerization (Figure 1b) with early transition metals (*i.e.*, Zr, Ti, and Hf),^12^ serving as self-activating catalysts^13^ and to develop a new class of highly tuneable donor/acceptor metallocenes.^14^ However, further applications have not yet widely emerged, despite the paramount position occupied by indenyl and related ligands in organometallic chemistry and homogeneous catalysis.

Besides, in terms of synthetic approaches, prior examples of indenylborane complexes require the independent and usually non-trivial preparation of the bifunctional ligand prior to metal coordination.^12,13,15^ This typically entails the use of hard to handle haloboranes and presents some limitations in terms of regioselectivity since the boron fragment is almost exclusively incorporated at the C1 position. Although the post-synthetic functionalization of the simpler cyclopentadienyl ligands in compounds of type (η^5^-C_5_H_5_)ML_*n*_ is well-known,^16^ this more convenient approach has not been applied to indenyl complexes.

In the context of ligand post-synthetic functionalization, we recently disclosed a reversible Cp* to metal hydride migration in compound [(η^5^-C_5_Me_5_)Rh(PMe_3_)_2_] upon addition of bulky Au(I) fragments with concomitant formation of new (Cp*)CH_2_–Au bonds.^17^ In contrast, ligand C–H bond activation does not take place for the related indenyl compound [(η^5^-C_9_H_7_)Rh(PPh_3_)_2_] (**1**), where we could only detect the formation of Rh → Au bimetallic adducts.^18^ Bearing in mind the high electrophilicity of perfluorinated boranes, we hypothesized that those could succeed in the direct functionalization of the indenyl ligand in **1** and thus provide facile access to bifunctional complexes with catalytic potential. With this goal, we describe herein the reactivity of compound **1** with perfluorinated boranes B(C_6_F_5_)_3_ and HB(C_6_F_5_)_2_, demonstrating that the boron functionality readily incorporates into the indenyl moiety (Figure 1c). The resulting complexes transcend previous related systems, which are mostly based on highly acidic early transition metals. Moreover, they are genuine motifs to investigate metallic FLP-type cooperativity^19^ because of the contrasting Lewis basic and acidic nature of the Rh(I) and borane fragments, respectively. In this vein, we provide preliminary studies that provide evidence of enhanced catalysis by using the hydrogenation of olefins as a model reaction.

## Results and Discussion

**Scheme 1:**
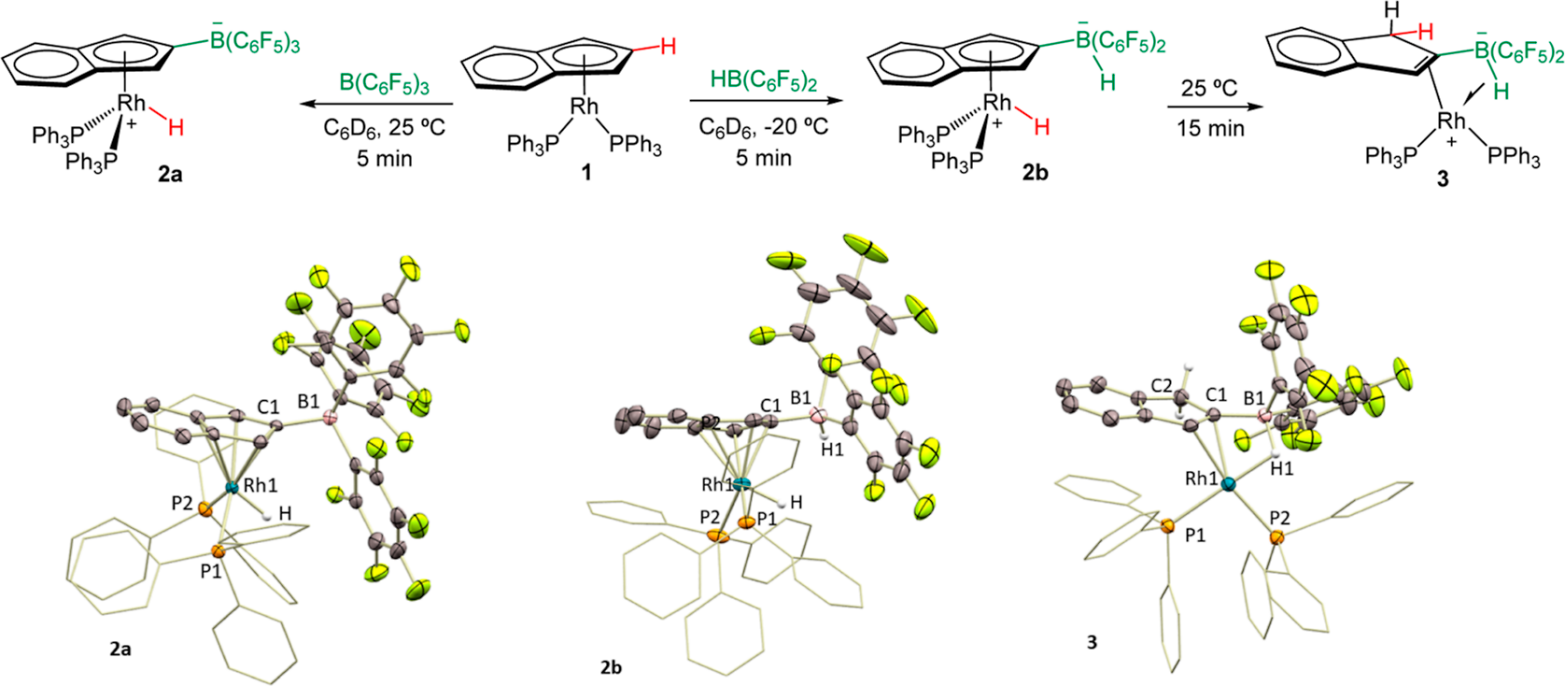
Activation of the Indenyl Ligand in Compound **1** upon Addition of Perfluorinated Boranes B(C_6_F_5_)_3_ and HB(C_6_F_5_)_2_ ORTEP diagrams of complexes **2a**, **2b**, and **3** are represented. Most hydrogen atoms are excluded for clarity, and thermal ellipsoids are set at 50% probability.

We first treated a benzene solution of compound **1** with an equimolar amount of the highly electrophilic B(C_6_F_5_)_3_ borane at 25 °C. This reaction rapidly leads to the activation of the C–H bond of the indenyl fragment at the C2 position toward compound **2a** (Scheme 1), where the new C–B bond results in a down-shifted ^11^B{^1^H} NMR resonance at −14.5 ppm [cf. 60 ppm for B(C_6_F_5_)_3_]. The migration of a hydride from the C2 position of the indenyl moiety generates a Rh–H bond, with the hydride resonating at −13.08 ppm (^1^*J*_HRh_ = 22.9, ^2^*J*_HP_ = 20.9 Hz) in the ^1^H NMR spectrum. This complex remains stable in solution for prolonged periods of time under an inert atmosphere.

The spontaneous formation of **2a** represents a rare case of electrophilic substitution of the coordinated indenyl fragment and, as we anticipated, is a very convenient route to access bifunctional ligands of this kind. This contrasts with all prior examples that required the independent synthesis of the borylated indene precursor. Moreover, placing the boron function at the C2 position of the indenyl ligand, as observed for compound **2a**, has remained elusive so far, and it was only accessed in unselective and very low-yielding syntheses.^13b^

Aside from trisubstituted B(C_6_F_5_)_3_, Piers’ borane HB(C_6_F_5_)_2_^20^ seems more suitable to access an active pendant borane functionality since the resulting borate moiety would contain a B–H bond susceptible to participating in chemical transformations.^4c,4d,6a^ Despite its reduced electrophilicity, this borane similarly reacts with compound **1** to yield the corresponding indenyl activated product **2b** (Scheme 1). Not surprisingly, the spectroscopic multinuclear NMR signature of **2b** is analogous to that of **2a** except for a new resonance in the ^1^H NMR spectrum at 4.70 ppm, which is attributable to the B–H terminus that sharpens upon decoupling from ^11^B.

Remarkably, this compound readily evolves at room temperature in solution to form the new species **3** (Scheme 1) that clearly differs from compounds **2** by NMR spectroscopy. The asymmetry of **3** is exemplified by two resonances in its ^31^P{^1^H} NMR spectrum at 42.2 (dd, ^1^*J*_PRh_ = 179, ^3^*J*_PP_ = 42 Hz) and 38.1 (dd, ^1^*J*_PRh_ = 183, ^3^*J*_PP_ = 42 Hz) ppm, contrasting with singlet resonances in the case of compounds **2a** and **2b** (37.8 and 41.2 ppm, respectively). ^19^F{1H} NMR reveals the presence of two nonequivalent fluorinated arenes that do not interconvert in the NMR time-scale, a feature that is exclusive of this system among all complexes reported in this work. Thus, two clearly separated signals due to the ortho-fluorine atoms are recorded by ^19^F{^1^H} NMR at −129.8 and −128.1 ppm. The symmetry of the indenyl ligand is broken as well. Two overlapped distinctive resonances arising from the diastereotopic geminal protons emerge at 3.78 ppm, further suggesting a loss of aromaticity. Finally, the hydridic signal in the ^1^H NMR spectrum exhibits a notable shift to higher frequencies from −13.72 in **2b** to −7.21 ppm in **3**, and now it sharpens upon ^11^B decoupling.

**Figure 2:**
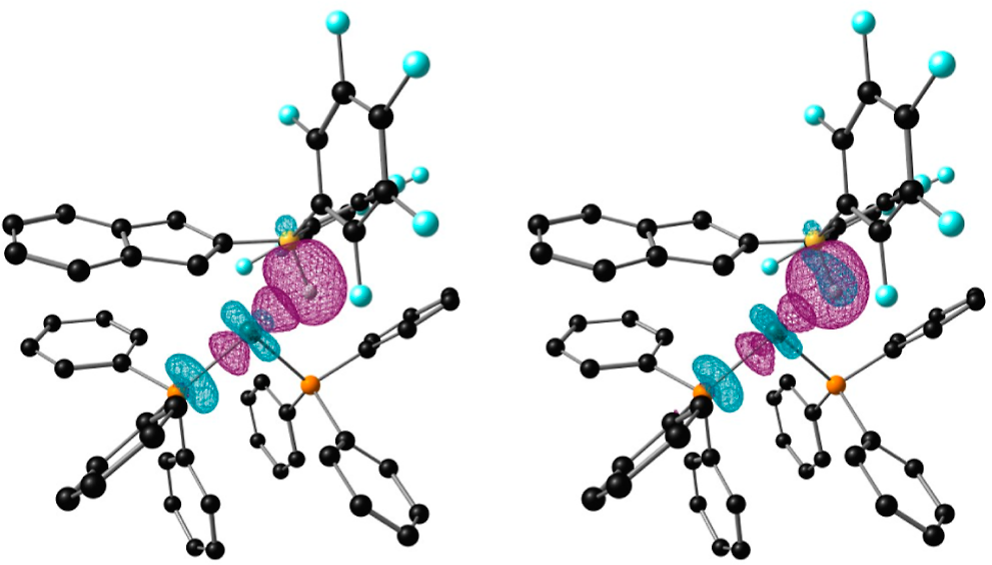
NBOs (left) and NLMOs (right) 261 (donor, BD B–H) and 262 (acceptor, BD* Rh–P) were calculated for complex **3**. Most H atoms were omitted for clarity.

The structure of compounds **2a**, **2b**, and **3** was authenticated by X-ray diffraction studies (Scheme 1).^21^ Compounds **2a** and **2b** exhibit the expected structure anticipated by spectroscopic analysis, with C–B bond distances [1.650(5), **2a**; 1.616(6) Å, **2b**] comparable to prior examples^15^ and other geometric parameters ranging normal values. The structure of **3** is more intriguing and can be described as a distorted square-planar Rh(I) species with two phosphine ligands and an η^2^-indene coordinated as an olefin that chelates the metal through an additional interaction with a BH fragment. We anticipated the latter interaction to be described as a 3-center-2-electron σ-borate complex. Related motifs have been previously rationalized as boron-hydrides stabilized by electrophilic metals^22^ or as metal hydrides that interact with pendant borane functions.^23^ Bearing in mind the intrinsic uncertainty of locating hydrogen atoms by X-ray diffraction techniques, the structure is defined by Rh–B and Rh–H bond distances of 2.412(2) and 1.80(3) Å and an acute B–H–Rh angle of 103.55(10)°, likely imposed by its intramolecular nature in a four-membered ring heterocycle. The bridging nature of the hydride fits well with the low-frequency signal recorded by ^1^H NMR (δ – 7.21).^24^ In turn, the relatively short Rh–B distance of 2.41, only moderately above the sum of their covalent radii (2.26 Å),^25^ suggests the direct participation of the three elements in a σ-type complex. We further examined the bonding situation in complex **3** by computational studies. The Quantum Theory of Atoms in Molecules (QTAIM) analysis carried out (Figure S33) shows bond critical points and paths between H and both Rh and B but not between Rh and B. In addition, the Lewis-type NBO analysis describes the B–H–Rh interaction as a B–H bond delocalizing into a σ* Rh–P orbital (Figure 2). Altogether, these results fit well with a description as a σ-borate rhodium complex. It is worth noting that the formation of **3** implies the migration of a hydride from the rhodium atom to the C1 position of the indenyl ligand, which losses its C_5_-aromaticity, somewhat resembling the well-known dearomatization pathways described by Milstein.^1a^ Moreover, that precise hydride ligand originates from a prior migration from the C2 position of the indenyl moiety upon addition of Piers’ borane (vide infra computational studies). Therefore, we disclose here a rare non-innocent behavior of the indenyl ligand that consists of an overall sequential two-step 1,2-H migration promoted by an electrophile, a unique process that to the best of our knowledge has remained undisclosed and that adds to the already rich chemistry of indenyl ligands.

**Figure 3:**
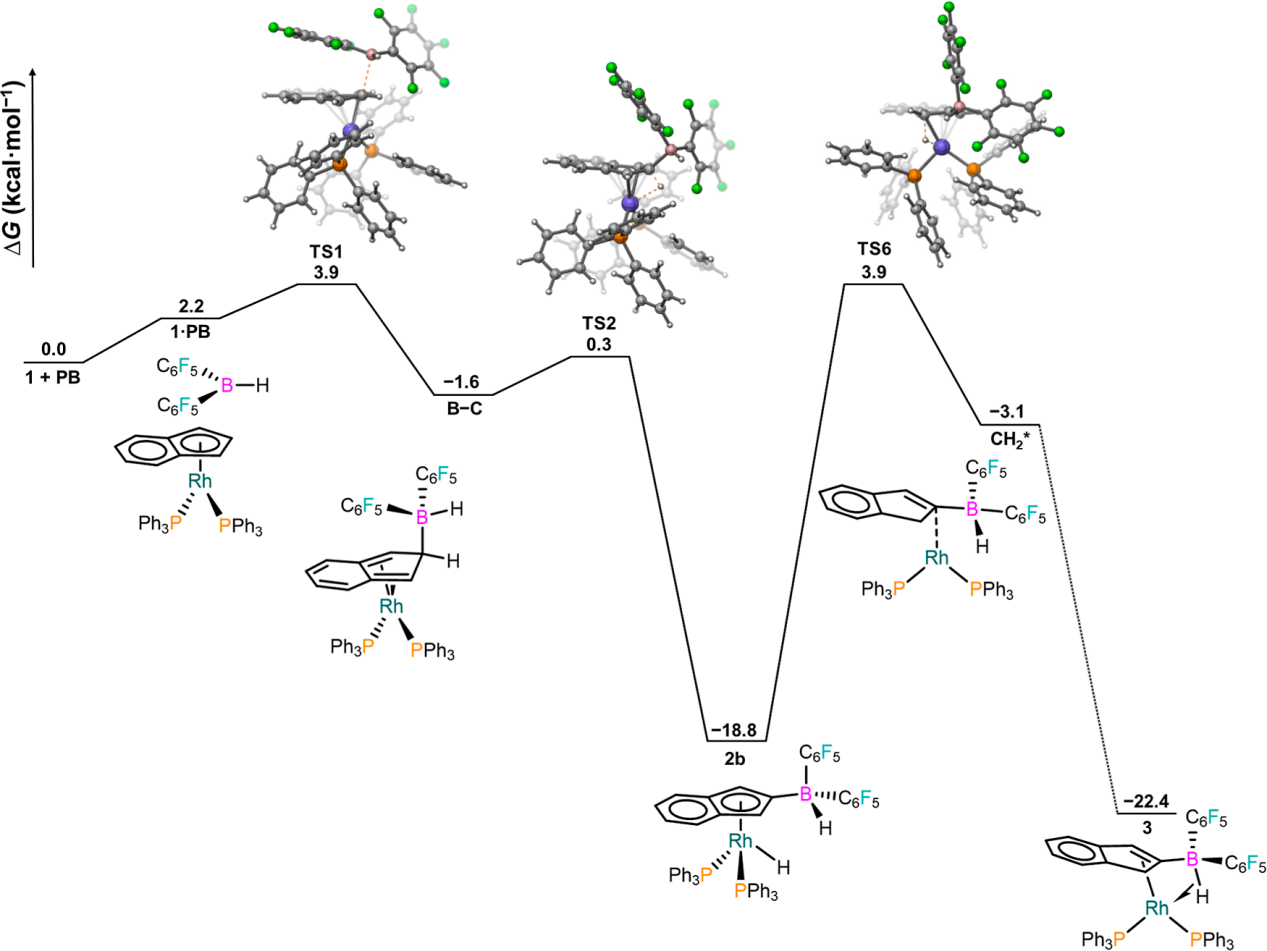
Free energy profile for the conversion of **1** and Piers’ borane into **3** at the SMD(dichloromethane)-PBE0-D3(BJ)/SDD(Rh)/6-311+G(2d,p)//SMD(dichloromethane)-PBE0-D3(BJ)/SDD(Rh)/6-31G(d,p) level of theory. The conversion of intermediate CH_2_* into its conformer **3** is expected to be facile (see Figure S35).

We naturally questioned ourselves about the precise mechanism to account for this process, which we investigated by computational means using Gaussian 09 (Revision E.01) software^26^ with the dispersion corrected PBE0-D3 functional (Figure 3).^27^ The electrophilic attack of the borane to the indenyl presents a very low barrier (TS1) to give a square planar Rh(I) diene complex (B–C), from which hydride transfer to Rh (also viewed as a C–H bond oxidative addition), concomitant with the restoration of aromaticity, is both facile (TS2) and largely exergonic. The most challenging step is the direct migration of the metal hydride to the indenyl (TS6, which also may be understood as a reductive elimination), in good agreement with the detection of intermediate **2b**. Once the CH_2_ moiety has been formed, coordination of the olefin to Rh gives complex **3**. We monitored the conversion of **2b** into **3** by ^31^P{^1^H} NMR spectroscopy (Figure S33), leading to a pseudo-first-order kinetic profile (*k* = 0.0011 s^–1^) corresponding to a Δ*G*_298_^‡^ of 21.5 kcal/mol, in excellent agreement with the calculated barrier (22.7 kcal/mol). Nonetheless, an alternative pathway involving the formation of an agostic B–H complex via TS3, which precedes the transfer of the metal hydride (TS4, 7.1 kcal/mol) from complex **2b**, was found to be only slightly higher in energy (Figure S36) and cannot therefore be ruled out.

The non-innocent behavior of the indenyl ligand upon reaction with Piers’ borane encouraged us to explore the catalytic potential of this system. To do so, we carried out preliminary investigations on the catalytic hydrogenation of olefins using rhodium precursor **1** and its boron-containing derivatives **2a** and **3**. For convenience, we selected the hydrogenation of styrene as a benchmark reaction to gauge the effect of the pendant borates. Hydrogenation of styrene toward ethylbenzene under mild conditions (25 °C, 0.5 atm of H_2_, 0.5 mol % [Rh]) proceeds in good yields after 1 h of reaction (Table 1) and, more importantly, provides a first hint on the effect of incorporating boron functionalities into the indenyl ligand.

**Table 1:**
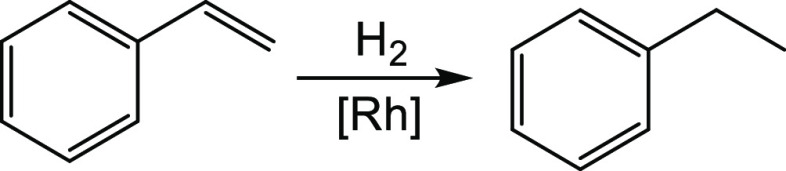
Catalyst Screening for the Hydrogenation of Styrene

entry	conditions^a^	cat	yield (%)^b^	TOF (h^–1^)
1	A	**1**	50	100
2	A	**2a**	63	126
3	A	**3**	81	162
4	B	**3**	100	58,823
5	C	**3**	25	125,000
6	B	**1**	trace	

a Conditions: (A): [Rh] 0.5 mol %, H_2_ (0.5 atm), 25 °C, 1 h, C_6_D_6_ (0.6 mL). (B) [Rh] 1 ppm, H_2_ (4 atm), 60 °C, 17 h, neat. (C) [Rh] 0.1 ppm, H_2_ (4 atm), 60 °C, 20 h, neat.

b ^1^H NMR yields using hexamethylbenzene as an internal standard.

From the results depicted in Table 1, it is clear that catalysis is enhanced in the presence of the borate functionality but only to a minor extent under rather mild conditions. More precisely, catalytic performance spans from 50% yield of ethylbenzene formation for Rh(I) precursor **1** (entry 1) up to 63 and 81% for the bifunctional systems **2a** and **3**, respectively (entries 2 and 3). As foreseen, the best catalytic performance is achieved with compound **3**, where the noninnocence behavior of the indenyl-boron ligand was already demonstrated by means of hydrogen migration events with the rhodium site. Next, we interrogated catalyst **3** and unfunctionalized **1** under harsher conditions. In particular, increasing the temperature to 60 °C and the H_2_ pressure to 4 atm and performing the catalysis in the absence of solvent allowed us to reach turnover numbers for compound **3** of up to 2.5 × 10^6^, associated with turnover frequencies (TOFs) of around 1.25 × 10^5^ h^–1^ (entry 5). Under the same conditions, the difference between **1** and **3** becomes dramatic as the former revealed inactive (entry 6). We exposed compound **1** to the aforesaid conditions to check whether the absence of catalyst results from limited stability, though **1** exhibited remarkable stability. Therefore, as discussed in detail in the following sections, the drastically increased activity of compound **3** derives from a more complex catalytic scenario. Besides, poisoning experiments with mercury and carbon disulfide, although not definitive, suggest the homogeneous nature of the active species (see Table S2 for further details).

**Scheme 2:**
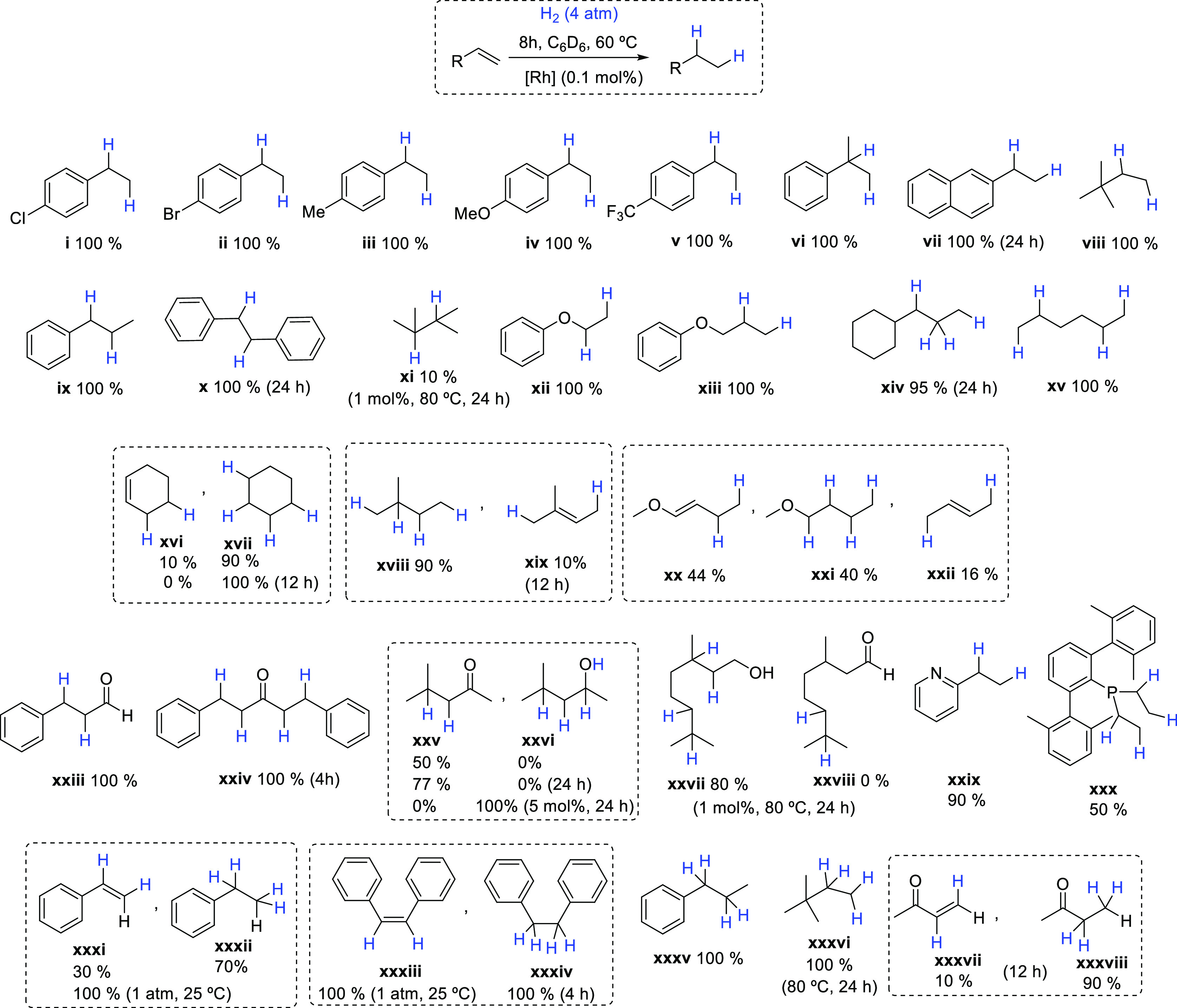
Preliminary Studies on Substrate Scope for Complex **3** as a Hydrogenation Catalyst (in Blue are the Hydrogens Added during Hydrogenation) Conditions that deviate from standard are indicated in parentheses.

We performed some preliminary investigations on the substrate scope with a series of unsaturated substrates, as shown in Scheme 2 (see the Supporting Information for experimental details). Aromatic and aliphatic alkenes, containing both electron-donating and electron-withdrawing groups, were readily hydrogenated under standard conditions (**i–viii** in Scheme 2). Internal olefins were also reduced (**ix–x**), though for the more challenging tetramethylethylene (**xi**), only low conversions were achieved even under harsher conditions. A vinyl (**xii**) and an allyl (**xiii**) ether could also be hydrogenated with full conversion without hydrogenolysis of the C–O bond, and similar results were obtained with an allene (**xiv**) and a terminal diene (**xv**). For other dienes (**xvi**–**xxii**), double hydrogenation of the two olefinic functions generally prevailed, though other isomers were also detected in variable amounts. α,β-unsaturated carbonyl compounds were selectively reduced at the olefinic C=C function, while the C=O bond remained intact (**xxiii–xxv**) unless longer reaction times and higher catalyst amounts are used (**xxvi**). The hydrogenation of terminal olefins attached to common ligands, as pyridine and phosphine, was also tested considering the inhibitor effect of free phosphines on other rhodium catalysts.^28^ Nonetheless, for this system, both vinylpyridine (**xxix**) and a divinyl terphenyl phosphine (**xxx**) could be hydrogenated in 90 and 50% yield, respectively. In the same vein, a catalytic run for styrene hydrogenation in the presence of two additional equivalents of PPh_3_ did not lead to catalysis decay. Finally, the hydrogenation of alkynes and α,β-unsaturated derivatives (**xxxi–xxxviii**) was also explored and led to reduced products in good yields, with specific regioselectivity with respect to semi- *vs* full-hydrogenation being controlled by tuning experimental conditions.

**Scheme 3:**
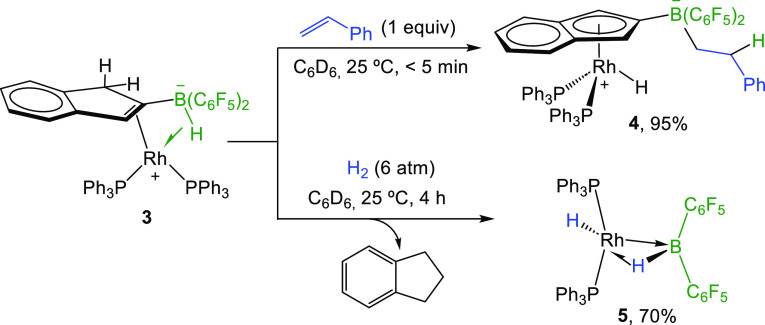
Stoichiometric Reactivity of Compound **3** towards Styrene and Dihydrogen

Aside from its direct use in catalysis, we decided to investigate the hydrogenation mechanism by both experimental and computational means in order to devise future and more challenging applications. We first carried out several stoichiometric experiments to shed some light on potential catalytic intermediates or resting states. Addition of one equivalent of styrene to a freshly prepared C_6_D_6_ solution of **3** resulted in immediate full conversion into a new species **4** (Scheme 3). Its formation is accompanied by a simplification of the corresponding ^31^P{^1^H} NMR spectrum to a single signal at 36.5 (d, ^1^*J*_PRh_ = 145 Hz) ppm in **4**. The higher symmetry of the latter species is further certified by the simpler ^1^H NMR pattern exhibited by the indenyl ligand (δ 6.96, 6.37, and 5.21 due to two protons each), which has recovered its aromaticity and η^5^-coordination. In addition, a low-frequency signal is now apparent at −13.70 ppm due to a Rh–H ligand, and no additional signals due to B–H units are visible. Instead, two signals due to two protons each at 3.03 and 2.01 ppm suggest the olefin to be inserted into the B–H bond. This occurs with concomitant regain of indenyl aromaticity, which by analogy to the DFT profile depicted in Figure 3, we attribute to the return of a hydrogen from the C1 position of the indenyl moiety to rhodium. Once more, this unusual insertion of the olefin into a borate B–H bond^3e^ demonstrates the reversible nature of the hydrogen migrations between the metal, the indenyl ring, and the boron function, again resembling the reversible aromatization/dearomatization routes described by Milstein as a key feature for catalysis.^1a^

**Figure 4:**
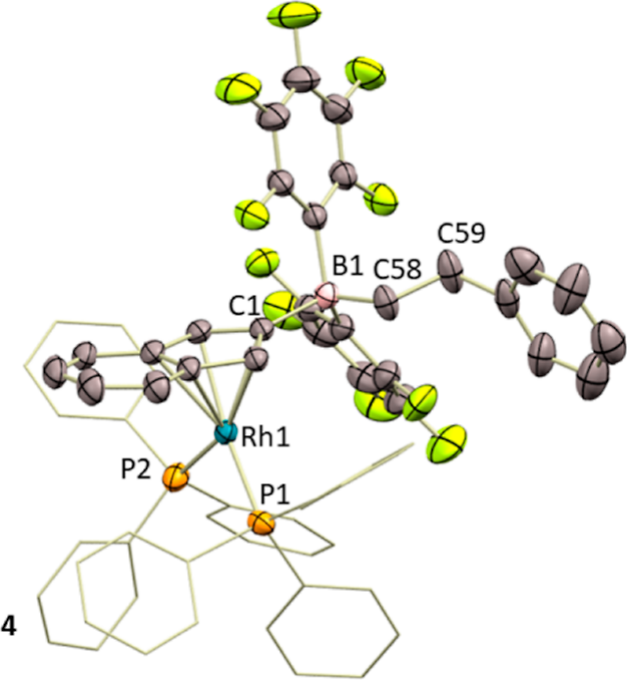
ORTEP diagram of complex **4**. Hydrogen atoms are excluded for clarity, and thermal ellipsoids are set at 50% probability.

Figure 4 depicts the X-ray diffraction structure of **4** that corroborates our NMR-based formulation. The structure is analogous to that of **2b** according to all of the geometric parameters. However, as opposed to the transformation of **2b** into **3**, in this case the absence of a stabilizing B–H unit seems to prevent the migration of the rhodium hydride toward the indenyl ring, which would otherwise result in a highly unsaturated metal site.

Contrarily, exposure of C_6_D_6_ solutions of **3** to a H_2_ atmosphere under catalytically relevant conditions (6 atm, 25 °C) did not provoke the immediate disappearance of the complex, as was observed after styrene addition. In this case, it required longer times (*ca.* 4 h) to produce a new species **5** in around 70% spectroscopic yield (Scheme 3). Its formation is accompanied by the appearance of indane (C_9_H_10_) in equimolar amounts [representative signals at ^1^H NMR, δ = 2.69 (t, ^3^*J*_HH_ = 7.4 Hz), 1.77 (q, ^3^*J*_HH_ = 7.4 Hz); and ^13^C{^1^H} NMR, δ = 32.7, 25.3] due to hydrogenation and C–B bond cleavage at the indenyl ligand. In turn, complex **5** is characterized by ^31^P{^1^H} and ^11^B{^1^H} resonances at 38.2 (^1^*J*_PRh_ = 115 Hz) and −1.2 (br) ppm, respectively. Nonetheless, the most distinctive feature of this compound is the presence of two low-frequency signals in the ^1^H NMR spectrum at −1.23 and −16.33 due to one proton each. Decoupling from either ^11^B or ^31^P causes the respective aforesaid resonances to clearly sharpen (Figure S21), suggesting that the lower-field resonance is directly coupled to the boron center as a B–H unit, while the higher-field signal is more influenced by the phosphine ligands. The chemical shift of the BH unit along with the geometrical parameters commented on below is indicative of some degree of σ-borane complex character, as later discussed in the context of computational studies. Besides, it has been recognized that the separation between *meta* and *para* fluorine atoms (Δ_m,p_) of perfluorinated boranes is indicative of the coordination mode of the borane.^29^ For compound **5**, Δ_m,p_ equals 3.3 [δ_F_ – 164.1 (F_m_), −160,8 (F_p_)], a slightly higher value than that in compounds **2b** (Δ_m,p_ = 2.5) and 4 (Δ_m,p_ = 2.8), in agreement with a less anionic character of the boron atom. However, this value is mildly lower than that for **3** (Δ_m,p_ = 4.6), as expected for a stronger coordination of the boron center to the metal in complex **5**. In solution, compound **5** exhibits dynamic behavior that accounts for the exchange of the Rh–H hydride with free dihydrogen and, at a lower rate, the intramolecular exchange between the two hydrides. Both processes were investigated by 2D-EXSY experiments, and the details are discussed in the Supporting Information (Figures S27 and S32).

**Figure 5:**
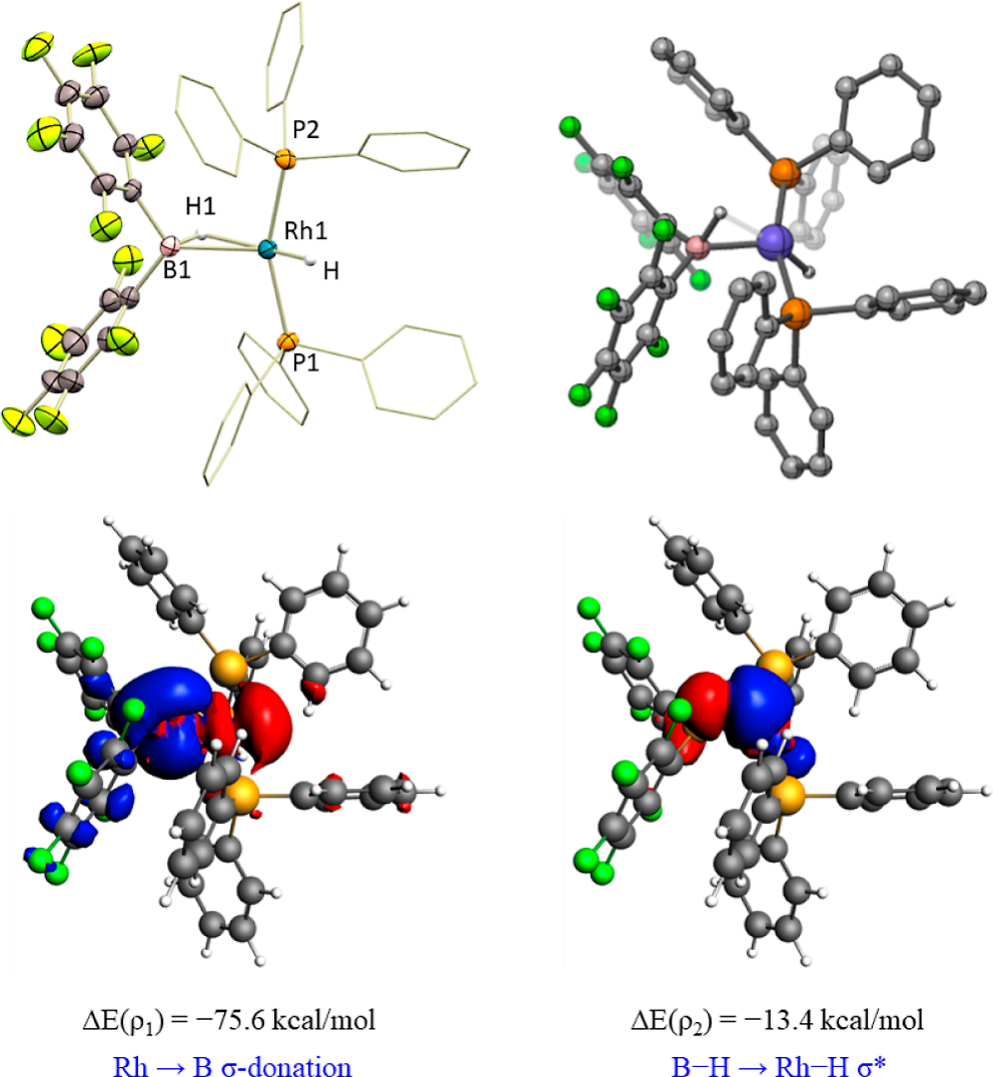
ORTEP diagram of complex **5**. Most hydrogen atoms are excluded for clarity, and thermal ellipsoids are set at 50% probability (up, left) and its DFT-optimized structure (up, right). Contour plots of NOCV deformation densities Δρ and associated energies Δ*E*(ρ) in **5** (down). Electron-density charge flows in the direction red → blue.

The molecular structure of **5** was again corroborated by X-ray diffraction studies (Figure 5), revealing the absence of the indenyl ligand, as deduced from NMR analysis, and the formation of a highly unsaturated Rh → borane adduct. The departure of the indenyl ligand has facilitated the rearrangement of the phosphines, which are now located in a trans disposition defined by a P–Rh–P angle of 156.58(5)°. The two hydride-type ligands were located in the Fourier electron density map, with the lowest-energy configuration found by DFT matching well the experimental geometry and suggesting that they are also located trans to each other (Figure 5, up; H–Rh···H angle of 164.65°, H–Rh distances of 1.56 and 1.84 Å, the latter presenting a B–H bond distance of 1.31 Å). The crystallographic B–Rh distance is considerably shorter than that in **3**, with a value of 2.316(5) Å, only slightly above of the sum of the corresponding covalent radii (2.26 Å)^25^ and in principle consistent with a strong dative interaction. The geometry around the boron center (not accounting for its hydride) is perfectly planar, as evinced by the sum of its three angles with Rh, C37, and C43 that accounts for an ideal 360°.

In principle, two extreme bonding scenarios could be envisioned for species **5**: a Rh(III) dihydride that features a B···H interaction with a boryl ligand, or a Rh(I) hydride with a Z-type borane ligand (BR_2_H) that engages in Rh → B and (B–H) → Rh interactions. EDA-NOCV indicates that the Rh–B bond stems from donation from the electron-rich Rh center to the B atom as the major contribution [Δ*E*(ρ_1_) = −75.6 kcal/mol] (Figure 5, down). The delocalization of the B–H bond onto the terminal Rh–H σ* orbital finds support by NBO (Figure S37) and EDA-NOCV (Figure 5, bottom), although it contributes to the stabilization of the Rh → B adduct to a lesser extent [Δ*E*(ρ_2_) = −13.4 kcal/mol]. In contrast, QTAIM analysis reveals bond critical points and paths for the B–H, B–Rh, and terminal Rh–H bonds, but not between the B–H hydrogen atom and the Rh center (Figure S34), which can still be consistent with a chemical bond between two atoms.^30^ Therefore, we propose that structure **5** may be better described as a virtually unsupported transition metal-borane adduct. Braunschweig recently described a related unsupported Pt → borane adduct that could be presumably identified in solution but which readily evolved through B–F bond activation toward zwitterionic species.^31^ The instability of these sought-after motifs has been partly attributed to the required deformation energies to access pyramidal conformations at boron^32^ which, in our case, is likely compensated by the weak (B–H) → Rh interaction.

**Figure 6:**
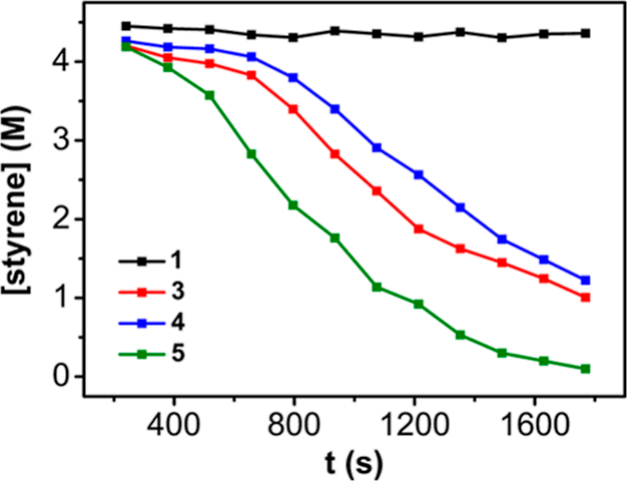
Comparison of the kinetic profiles for the hydrogenation of styrene for complexes **1**, **3**, **4**, and **5**. Conditions: [Rh] 0.001 mol %, H_2_ (6 atm), 25 °C, C_6_D_6_ (0.6 mL).

Next, we tested the catalytic competence of compounds **4** and **5** compared to **3** and the initial precursor **1**, although the high instability of the former complexes precluded a fully precise evaluation. In fact, while compound **4** could be isolated in acceptable purity and tested as a catalyst, the isolation of **5** in the pure form escaped our efforts. As such, we generated compound **5***in situ* under a hydrogen atmosphere and then added to the resulting solution styrene and fresh H_2_ gas to monitor the catalytic reduction of the olefin by ^1^H NMR. The resulting kinetic profile for catalysts **3**, **4**, and **5** is comparatively similar (Figure 6), only differing in a slightly reduced induction period for the freshly prepared solution of **5**. It is possible that Piers’ borane acts as an activating agent, facilitating the complete reduction and release of the indenyl ligand to yield **5**, whose remarkable apparent simplicity may unlock the rather enhanced catalytic performance. Thus, it is possible that the active species in this system does not contain a pendant borane function but rather the cooperative action of a highly unsaturated Rh fragment and Piers’ borane and that the role of *in situ*-generated weakly coordinating borate anions is also important.

**Figure 7:**
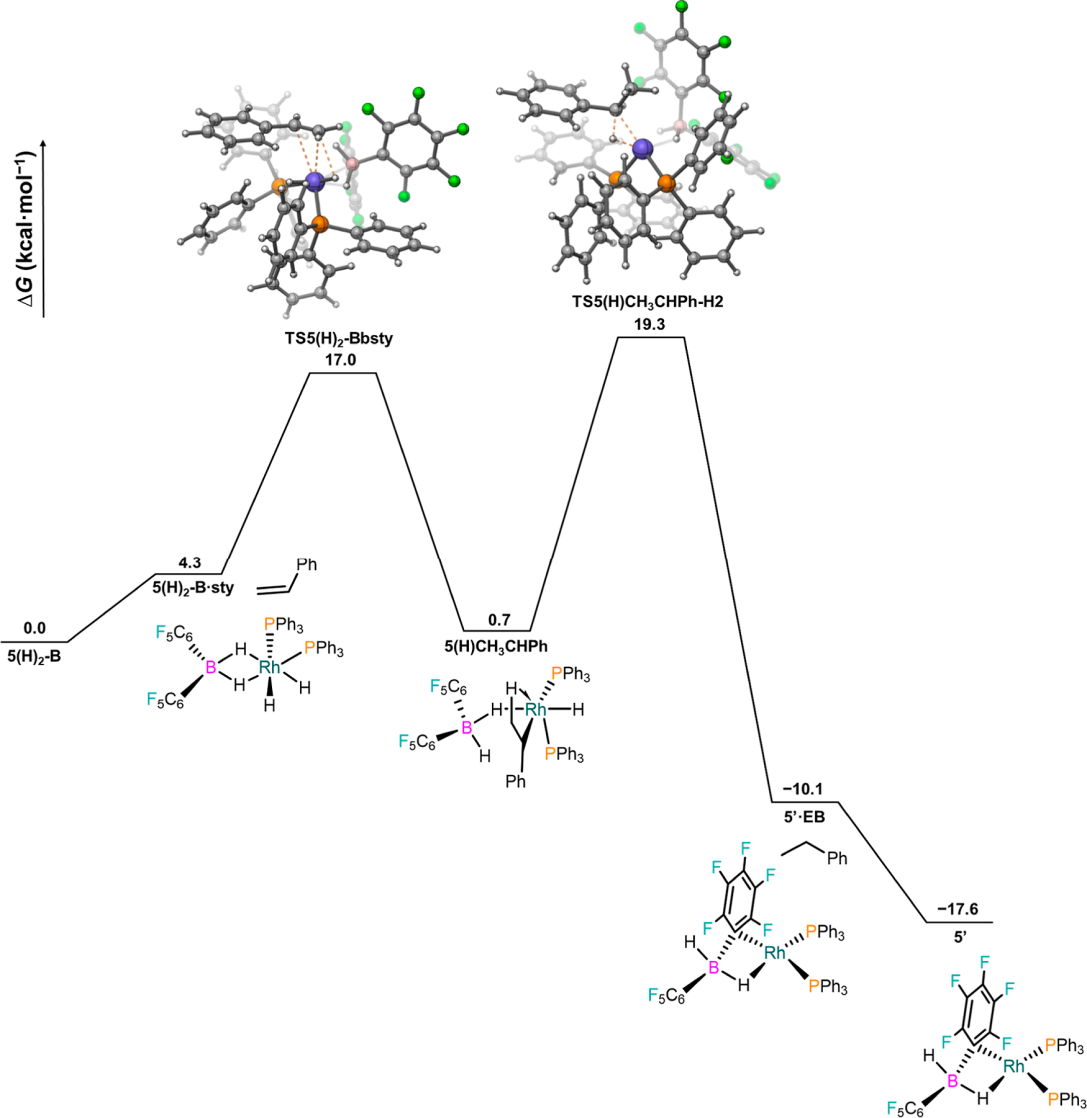
Free energy profile for the hydrogenation of styrene from 5(H)_2_–B at the SMD(dichloromethane)-PBE0-D3(BJ)/SDD(Rh)/6-311+G(2d,p)//SMD(dichloromethane)-PBE0-D3(BJ)/SDD(Rh)/6-31G(d,p) level of theory.

In this regard, we computationally investigated the reactivity of complexes **3** and **5** toward H_2_. While for complex **3** both the coordination and oxidative addition of H_2_ are endergonic (Figures S37 and 38), the more unsaturated **5** displays only mildly endergonic formation of a σ–H_2_ complex, from which oxidative addition leads to the exergonic generation of borate-bound species **5(H)**_**2**_ (Figures S39 and 40). Based on these results and the superior catalytic profile of **5** compared to **3** (Figure 6), we focused on exploring pathways for hydrogenating styrene based on **5** and **5(H**_**2**_**)**. We have found that the latter species can effectively hydrogenate styrene (Figure 7): a shift to the κ^1^ coordination mode from the borate opens a vacant site that allows styrene coordination [TS5(H)_2_-Bbsty, 17.0 kcal/mol], which is followed by a barrierless olefin insertion into a Rh–hydride. The resulting agostic complex evolves through reductive elimination via TS5(H)CH_3_CHPh-H_2_ (19.3 kcal/mol), yielding the exergonic formation of ethylbenzene (EB) and **5′**, a square planar species with greater stability than its isomer **5**.

**Figure 8:**
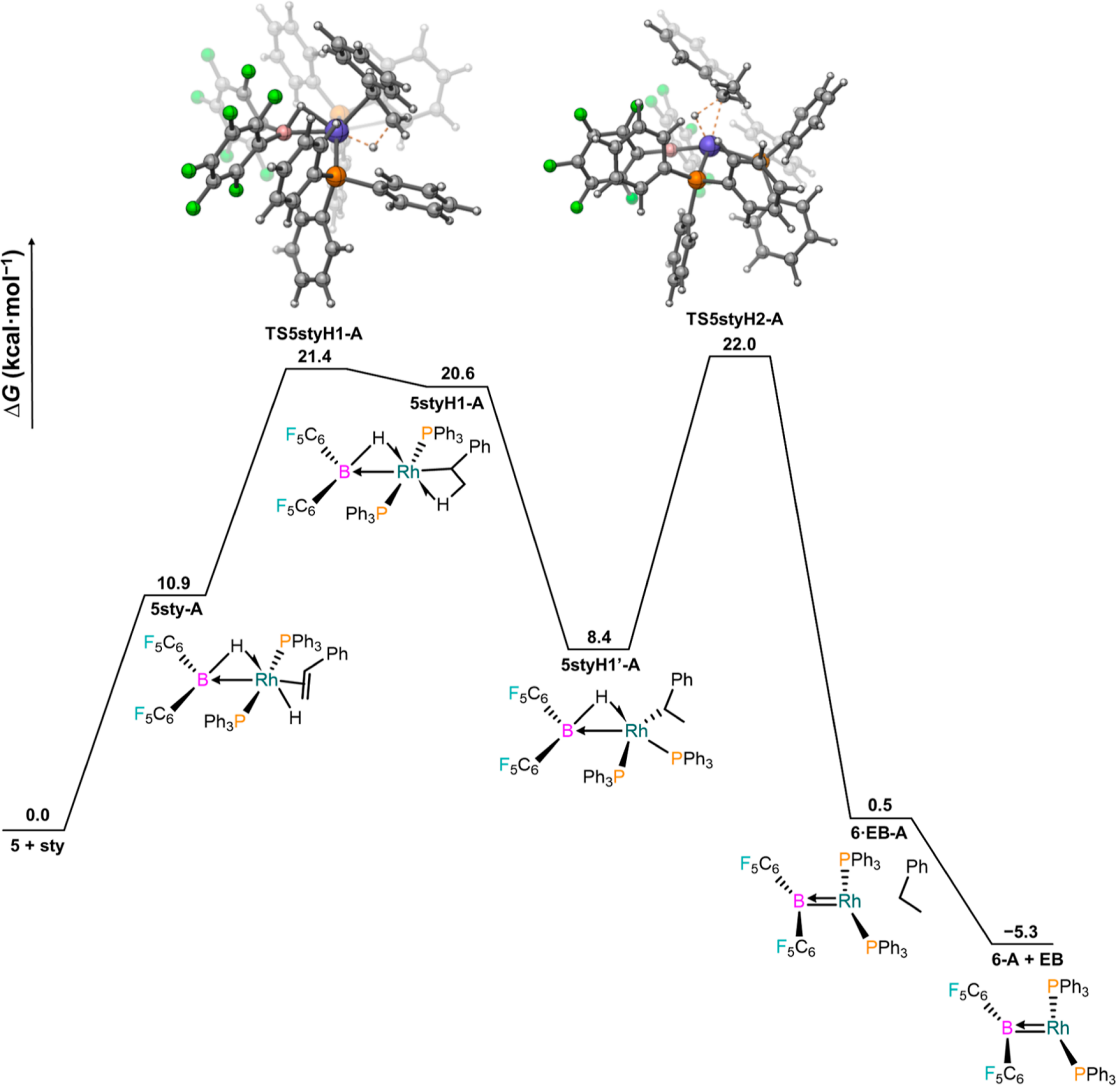
Free energy profile for the hydrogenation of styrene from **5** at the SMD(dichloromethane)-PBE0-D3(BJ)/SDD(Rh)/6-311+G(2d,p)//SMD(dichloromethane)-PBE0-D3(BJ)/SDD(Rh)/6-31G(d,p) level of theory.

Additionally, we investigated whether complex **5** could be catalytically competent through an alternative route involving direct transfer of its two metal-bound H atoms to styrene without the prior addition of H_2_. Indeed, Figure 8 shows that after styrene coordination (10.9 kcal/mol), both hydrides can be sequentially transferred via TS5styH1-A and TS5styH2-A, at 21.4 and 22.0 kcal/mol, respectively, giving the exergonic formation of ethylbenzene and the strongly unsaturated complex Ar_2_B–Rh(PPh_3_)_2_ (**6**), featuring a short Rh–B bond (1.91 Å). An alternative pathway is provided in Figure S42. NBO describes species **6** as Rh(I), according to 4 LPs on Rh, with the BAr_2_ fragment behaving as an anionic boryl ligand. Unsaturation at boron is compensated for by strong back-donation from Rh to the empty valence orbital of B (Figure S43). Contrary to **5**, EDA-NOCV indicates that for **6**, the electrostatic contribution is the major component of the bonding. However, the smallest value for the orbital interaction Δ*E*orb is obtained for the open-shell neutral fragments [Rh(PPh_3_)_2_]^•^ and [B(C_6_F_5_)_2_]^•^, which provide a bonding scenario consistent on a covalent σ bond and Rh → B π backdonation (Figure S44B). Importantly for catalysis, species **6** can facilely coordinate and oxidatively add H_2_ (13.4 kcal/mol), reforming complex **5** (Figure S45). Based on this, we attempted the reverse reaction, that is, dehydrogenation of **5** toward the unsaturated formally boryl complex **6**. Although our efforts to isolate and fully characterize such a compound have so far been unfruitful due to the high reactivity of these species, we could identify by NMR a major compound assigned to **6** after exposing solutions of **5** to dynamic vacuum. As expected, this species does not exhibit low-frequency ^1^H NMR hydride signals, while a ^31^P{^1^H} resonance is recorded at 43.9 Hz (^1^*J*_PRh_ = 211 Hz).

**Scheme 4:**
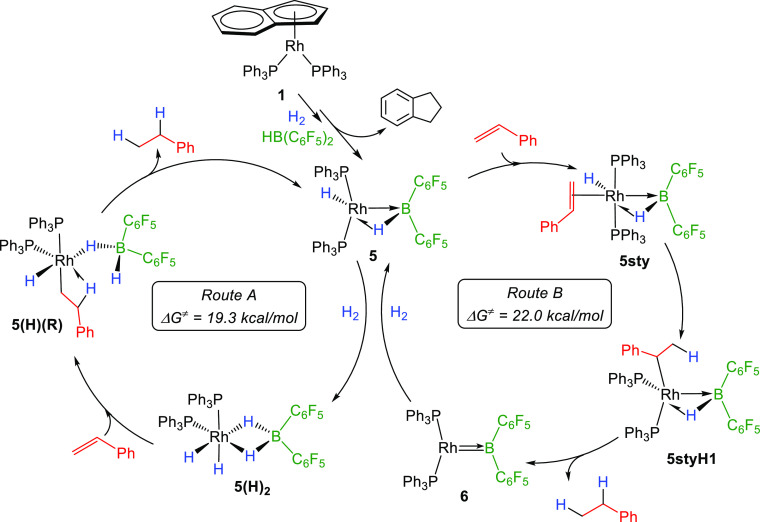
Proposed Competing Mechanisms for the Hydrogenation of Styrene Using the Combination of Precatalyst **1** and Piers’ Borane HB(C_6_F_5_)_2_ through Common Active Species **5**

Overall, it is rather clear that catalyst **3** containing the boron functionality is remarkably more efficient than its borane-free precursor **1** (Figure 6). Thus, while **3** reduces styrene with full conversion even at catalyst loadings as low as 0.001 mol %, the parent complex **1** only produces trace amounts of ethylbenzene under otherwise identical conditions. The above mechanistic investigations point to multiple roles of Piers’ borane to enhance catalysis. This is better exemplified in the overall mechanistic picture proposed in Scheme 4. On one hand, the borane functionalizes the indenyl ligand, facilitating its release as indane and giving access to a more reactive active species (**5**), characterized by a dative bond from rhodium to the borane. Thus, a second role of the borane entails the stabilization of the highly unsaturated Rh(I) fragment [Rh(H)(PPh_3_)_2_]. In addition, it also provides several plausible routes for Rh/B cooperation during the catalytic hydrogenation of styrene, the two main pathways being depicted in Scheme 4. Route A involves the initial oxidative addition of H_2_ onto **5**, while route B entails the initial coordination of the olefin, being both associated with Figures 7 and 8, respectively. In route A, the borane moiety directly participates in H–H bond cleavage across the Rh → B bond. Besides, the flexibility of the bridging hydrides enables the generation of a vacant site at the metal assisted by boron. In turn, the main intermediates in route B evince an active participation of the boron fragment both to stabilize the low coordinate fragment [Rh(PPh_3_)_2_] by means of formal boryl compound **6** and to activate dihydrogen across the Rh=B bond.

## Conclusions

The synthesis of boron containing transition-metal complexes has become a frontier approach toward innovative catalysts. At variance to the currently more extended methods involving the synthesis of sophisticated bifunctional ligands containing N- and P-donors, we exploit here a convenient postsynthetic functionalization procedure. This entails the direct treatment of electron-rich compound [(η^5^-C_9_H_7_)Rh(PPh_3_)_2_] with perfluorinated boranes, an approach that had been almost exclusively examined in the context of early transition-metal polymerization catalysis for simpler C_5_H_5_-based systems. We demonstrate that borate functions can be easily installed on the indenyl ligand through the formation of new C–B bonds. Importantly, this triggers an unprecedented stepwise 1,2-hydride shift at the indenyl moiety that evinces potential for catalysis. We investigated this potential for the benchmark hydrogenation of olefins. In this case, the catalyst efficiency is boosted up to 3 orders of magnitude compared to the boron-free precursor. Our mechanistic investigations suggest a complex role for the boron function, first as an activating fragment to access a highly unsaturated active species through indane release and, second, as a cooperative partner to accomplish catalytic hydrogenation by synergistic action with the rhodium center.

## Supporting Information Available

Experimental procedures, NMR spectra, crystal structure determinations, exchange EXSY experiments, details on catalytic studies, and computational details (PDF)
Crystallographic information (CIF)
Crystallographic information (CIF)
Crystallographic information (CIF)
Crystallographic information (CIF)
Crystallographic information (CIF)
Computational data (XYZ)

## Supplementary Material

cs3c02764_si_001.pdf

cs3c02764_si_002.cif

cs3c02764_si_003.cif

cs3c02764_si_004.cif

cs3c02764_si_005.cif

cs3c02764_si_006.cif

cs3c02764_si_007.xyz
