# Supplementary material for: Ligand Postsynthetic Functionalization with Fluorinated Boranes and Implications in Hydrogenation Catalysis
Source: ACS Catal. 2023 Nov 30;13(24):16055–66. doi: 10.1021/acscatal.3c02764 (PMC10852356; doi:10.1021/acscatal.3c02764)
Supplement: Supplementary file 1 — cs3c02764_si_001.pdf [file cs3c02764_si_001.pdf]

Supporting Information

Ligand post-Synthetic Functionalization with Fluorinated Boranes and Implications in Hydrogenation Catalysis

Macarena G. Alférez, Juan J. Moreno, Miguel A. Gaona, Celia Maya, Jesús Campos*

Instituto de Investigaciones Químicas (IIQ), Departamento de Química Inorgánica and Centro de Innovación en Química Avanzada (ORFEO-CINQA), Consejo Superior de Investigaciones, Científicas (CSIC) and Universidad de Sevilla, Avenida Américo Vespucio 49, 41092 Sevilla, Spain.

jesus.campos@iiq.csic.es

Abstract: The incorporation of boron functionalities into transition-metal catalysts have become a promising strategy to improve catalytic performance, though their synthesis typically entails the preparation of sophisticated bifunctional ligands. We report here the facile and direct post-synthetic functionalization of rhodium(I) compound [(η^5-C_9H_7)Rh(PPh_3)$_2$] (**1**) by treatment with perfluorinated boranes. Borane addition to **1** results in an unusual C(sp^2)-H hydride migration from the indenyl ligand to the metal with concomitant formation of a C–B bond. In the case of the Piers' borane (HB(C_6F_5)$_2$), this is followed by a subsequent hydride migration that leads to an unprecedented 1,2-hydrogen shift reminiscent of Milstein's cooperative dearomatization pathways. Computational investigations provide a mechanistic picture for the successive hydride-migration steps, which enrich the non-innocent chemistry of widespread indenyl ligands. Moreover, we demonstrate that addition of Pier's borane is highly beneficial for catalysis, increasing catalyst efficiency up to three orders of magnitude.

Table of Contents

Experimental procedures S2

NMR spectra S5

Crystal structure determinations S18

NMR reaction monitoring of catalytic runs S19

Exchange EXSY experiments for compound **5** S20

Optimization of catalytic conditions using complex **5** S24

Preliminary studies on substrate scope S25

Computational details S26

References S35

Experimental Procedures

General considerations

All preparations and manipulations were carried out using standard Schlenk and glove-box techniques, under argon or high-purity nitrogen atmosphere, respectively. All solvents were dried, stored over 4 Å molecular sieves, and degassed prior to use. Toluene (C_7H_8) and *n*-pentane (C_5H_{12}) were distilled under nitrogen over sodium. Benzene-d_6 and toluene-d_8 were dried over molecular sieves (4 Å). THF-d_8 was distilled under nitrogen over sodium/benzophenone. Rhodium complexes **1** and **2** were prepared according to previously reported procedures[1]. $B(C_6F_5)_3$ was acquired from commercial sources and sublimated prior to use and Piers´ borane ($HB(C_6F_5)_2$) was synthesized following a previously reported[2] procedure. Other chemicals were commercially available and used as received. Solution NMR spectra were recorded on Bruker AMX-300, DRX-400 and DRX-500 spectrometers. Spectra were referenced to external $SiMe_4$ (δ: 0 ppm) using the residual proton solvent peaks as internal standards (^{1}H NMR experiments), or the characteristic resonances of the solvent nuclei ($^{13}C\{^{1}H\}$ NMR experiments), while ^{31}P was referenced to H_3PO_4, ^{19}F to fluorotrichloromethane, and ^{11}B to $BF_3{\cdot}OEt_2$. Spectral assignments were made by routine one- and two-dimensional NMR experiments where appropriate. For elemental analyses a LECO TruSpec CHN elementary analyzer was utilized.

Synthesis and characterization of new compounds

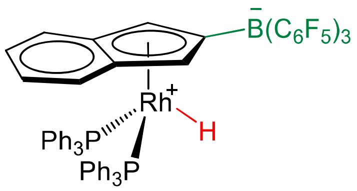

Compound 2a. In a scintillation vial inside the glovebox, a solid mixture of compound **1** (42 mg, 0.059 mmol) and $B(C_6F_5)_3$ (30 mg, 0.059 mmol) was dissolved in benzene or toluene (1.5 mL) at room temperature. Brownish crystals were grown by slow diffusion of pentane into the solution mixture in the glovebox freezer (−35 °C) (18 mg, 34%). Alternatively, a lower-scale reaction was carried out in a J. Young NMR tube inside the glovebox (**1**, 15 mg, 0.026 mmol; $B(C_6F_5)_3$, 15 mg, 0.028 mmol) and the reaction monitored by ^{1}H and $^{31}P\{^{1}H\}$ NMR Reaction monitoring revealed the immediate consumption of the precursors and the formation of **2a** as the major compound. Anal. Calcd. for $C_{63}H_{37}BF_{15}P_2Rh$: C, 60.3; H, 3.0. Found: C, 60.5; H, 3.1.

^{1}H NMR (400 MHz, toluene-d_8, −10 °C) δ: 6.98 (m, 4H, *o*-Ph), 6.82 (m, 8H, *o*-Ph,), 6.75 (m, 20H, overlapping *p*-Ph, *m*-Ph, Ind), 6.05 (m, 2H, Ind), 5.35 (m, 2H, Ind), −13.08 (apparent q, 1H, $^{1}J_{HRh}$ = 22.9 Hz, $^{2}J_{HP}$ = 20.9 Hz, RhH). $^{11}B\{^{1}H\}$ NMR (128 MHz, toluene-d_8, −10 °C) δ: −14.5. $^{13}C\{^{1}H\}$ NMR (125 MHz, toluene-d_8, −10 °C) δ: 148.7 (dm, $^{1}J_{CF}$ = 235 Hz, *o*-C_6F_5), 139.9-136.2 (overlapped with toluene signal, *p*- and m-C_6F_5), 133.3 (*m*-Ph), 132.8 (*p*-Ph), 130.1 (*o*-Ph), 127.7 (overlapping toluene signal, Ind),

122.0 (Ind), 117.5 (Ind), 116.0 (*ipso*-C_6F_5), 88.7 (Ind). $^{19}F\{^1H\}$ NMR (471 MHz, toluene-d_8, −10 °C) δ: −164.1 (br. t, $^3J_{FF}$ = 21 Hz, *m*-C_6F_5), −160.7 (t, $^3J_{FF}$ = 21 Hz, *p*-C_6F_5), −126.3 (br, *o*-C_6F_5). $^{31}P\{^1H\}$ NMR (162 MHz, toluene-d_8, −10 °C) δ: 37.8 (d, $^1J_{PRh}$ = 144 Hz).

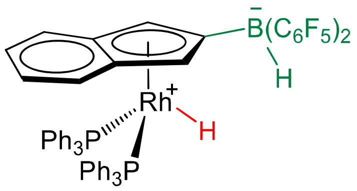

Compound 2b. In a scintillation vial inside the glovebox, a solid mixture of compound **1** (30 mg, 0.042 mmol) and $HB(C_6F_5)_2$ (20 mg, 0.057 mmol) was dissolved in toluene (1.0 mL) at −20 °C. Yellow crystals were grown by slow diffusion of pentane into the solution mixture in the glovebox freezer (−35 °C) (10 mg, 22%). To avoid further evolution of the complex towards **3** all manipulations were done keeping the solution temperature below −20 °C. Alternatively, a lower-scale reaction was carried out in a J. Young NMR tube inside the glovebox (**1**, 15 mg, 0.026 mmol; $HB(C_6F_5)_2$, 10 mg, 0.028 mmol) and the reaction monitored by ^1H and $^{31}P\{^1H\}$NMR reaction monitoring revealed the immediate consumption of the precursors and the formation of **2b** as the major compound.

^1H NMR (400 MHz, toluene-d_8, −10 °C) δ: 6.98 (m, 30H, overlapping PPh_3), 6.82 (m, 2H, Ind), 6.05 (m, 2H, Ind), 5.35 (m, 2H, Ind), 4.70 (br, 1H, BH), −13.10 (q, 1H, $^2J_{HP}$ = $^1J_{HRh}$ = 23.9 Hz, RhH). $^{11}B\{^1H\}$ NMR (128 MHz, toluene-d_8, −10 °C) δ: −22.1 (br). $^{13}C\{^1H\}$ NMR (125 MHz, toluene-d_8, −10 °C) δ: 148.5 (dm, $^1J_{CF}$ = 226 Hz, *o*-C_6F_5), 139.2 - 135.9 (overlapped with toluene signal, *p*- and m-C_6F_5), 135.5 (Ind), 134.0 (PPh_3), 133.3 (PPh_3), 129.9 (PPh_3), 127.1 (Ind), 121.5 (Ind), 119.1 (Ind), 115.8 (*ipso*-C_6F_5), 87.5 (Ind). $^{19}F\{^1H\}$ NMR (471 MHz, toluene-d_8, −10 °C) δ: −165.1 (m, *m*-C_6F_5), −162.6 (t, $^3J_{FF}$ = 21 Hz, *p*-C_6F_5), −130.5 (m, *o*-C_6F_5). $^{31}P\{^1H\}$ NMR (162 MHz, toluene-d_8, −10 °C) δ: 41.2 (d, $^1J_{PRh}$ = 145 Hz).

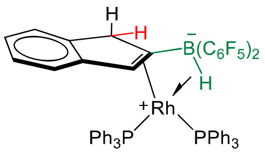

Compound 3. In a scintillation vial inside the glovebox, a solid mixture of compound **1** (30 mg, 0.042 mmol) and $HB(C_6F_5)_2$ (20 mg, 0.057 mmol) was dissolved in benzene or toluene (1.5 mL) at room temperature. Orange crystals were grown by slow diffusion of pentane into the solution mixture in the glovebox freezer (−35 °C) (18 mg, 39%). Alternatively, a lower-scale reaction was carried out in a J. Young NMR tube inside the glovebox (**1**, 15 mg, 0.026 mmol; $HB(C_6F_5)_2$, 10 mg, 0.028 mmol) and the reaction monitored by ^1H and $^{31}P\{^1H\}$ NMR reaction monitoring revealed the initial formation of **2b** which rapidly evolved towards **3** as the major species after around 15 min. Anal. Calcd. for $C_{57}H_{37}BF_{10}P_2Rh$: C, 63.0; H, 3.4. Found: C, 63.0; H, 3.2.

^{1}H NMR (500 MHz, toluene-d_8, −10 °C) δ: 7.63 (m, 6H, *o*-Ph), 7.13 (m, 6H, *o*-Ph,), 6.82 (m, 13H, overlapping *m*-Ph, Ind), 6.70 (m, 7H, overlapping *p*-Ph, Ind), 6.54 (m, 1H, Ind), 6.03 (m, 1H, Ind), 5.73 (m, 1H, Ind), 3.78 (m, 2H, CH_2), −7.21 (m, 1H, BH). ^{11}B{^{1}H} NMR (128 MHz, toluene-d_8, −10 °C) δ: −19.8 (br, $\omega_{1/2}$ = 250 Hz). ^{13}C{^{1}H} NMR (125 MHz, toluene-d_8, −10 °C) δ: 148.2 (dm, $^1J_{CF}$ = 235 Hz, *o*- C_6F_5), 136.2 - 132.9 (overlapped *m*-Ph, *p*- and *m*-C_6F_5), 127.1 (overlapping toluene *m*-Ph, *o*-Ph, *p*-Ph, Ind), 124.3 (Ind), 122.7 (Ind), 117.2 (*ipso*-C_6F_5), 106.7 (Ind), 44.2 (CH_2). ^{19}F{^{1}H} NMR (471 MHz, toluene-d_8, −10 °C) δ: −164.2 (m, *m*-C_6F_5), −159.6 (m, *p*-C_6F_5), −129.8 (m, *o*-C_6F_5), −128.1 (d, $^3J_{FF}$ = 22 Hz, *o*-C_6F_5). ^{31}P{^{1}H} NMR (162 MHz, toluene-d_8, −10 °C) δ: 42.2 (dd, $^1J_{PRh}$ = 179 Hz, $^3J_{PP}$ = 42 Hz), 38.1 (dd, $^1J_{PRh}$ = 183 Hz, $^3J_{PP}$ = 42 Hz).

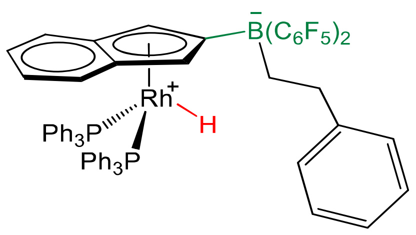

Compound 4. A solid mixture of compound **1** (30 mg, 0.042 mmol) and $HB(C_6F_5)_2$ (20 mg, 0.057 mmol) was dissolved in toluene (2 mL) under strictly inert conditions and styrene (2 µL, 0.042 mmol) was added. The solution was stirred at room temperature for 15 minutes. Reaction monitoring revealed the formation of **4**. The consumption of the precursors proceeded quantitatively by NMR spectroscopy, being **4** the major species. Orange crystals were grown by diffusion of pentane into the toluene solution of **4** in the glovebox freezer (15 mg, 30%). Anal. Calcd. for $C_{64}H_{44}BF_{10}P_2Rh$: C, 65.2; H, 3.8. Found: C, 65.3; H, 3.9.

^{1}H NMR (500 MHz, toluene-d_8, −10 °C) δ: 7.50 (m, 2H, o-styrene), 7.43 (m, 2H, PPh_3), 7.30 (m, 2H, m-styrene), 7.21 (m, 1H, *p*-styrene), 6.96 (m, 8H, Ind, PPh_3), 6.82 (m, 22H, PPh_3), 6.37 (m, 2H, Ind), 5.21 (m, 2H, Ind), 3.03 (m, 2H, CH_2), 2.01 (m, 2H, CH_2), −13.70 (apparent q, 1H, $^2J_{HP}$ = 23.2 Hz, $^1J_{HRh}$ = 22.8 Hz, RhH). ^{11}B{^{1}H} NMR (128 MHz, toluene-d_8, −10 °C) δ: −13.3 (br, $\omega_{1/2}$ = 265 Hz). ^{13}C{^{1}H} NMR (125 MHz, toluene-d_8, −10 °C) δ: 149.0 (*ipso*-styrene), 144.9 (Ind), 134.0 (PPh_3), 133.3 (PPh_3), 130.1 (PPh_3), 128.7 (*o*-styrene), 128.0 (*m*-styrene), 127.1 (PPh_3), 127.7 (PPh_3), 127.3 (Ind), 125.0 (*m*-styrene), 124.4 (*p*-styrene), 122.3 (Ind), 116.9 (Ind), 87.7 (Ind), 35.1 (CH_2), 26.6 (CH_2). Signals due to the fluorinated rings could not be unambiguously detected. ^{19}F{^{1}H} NMR (471MHz, toluene-d_8, −10 °C) δ: −164.6 (m, *m*-C_6F_5), −161.8 (t, $^3J_{FF}$ = 20 Hz, *p*-C_6F_5), −129.1 (br, *o*-C_6F_5). ^{31}P{^{1}H} NMR (162 MHz, toluene-d_8, −10 °C) δ: 36.5 (d, $^1J_{PRh}$ = 145 Hz).

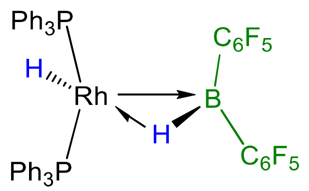

Compound 5. In a sealed NMR pressure tube or in a Fisher-Porter reactor a solid mixture of compound **1** (30 mg, 0.042 mmol) and $HB(C_6F_5)_2$ (20 mg, 0.057 mmol) was dissolved in C_7D_8 or C_6D_6 (0.5-2 mL) at

room temperature and stirred for 18 hours under H_2 atmosphere (6 atm). Reaction monitoring revealed quantitative consumption of the precursors and formation of compound **5** as the major species. Brown crystals of **5** were grown by slow diffusion of pentane into its toluene solution in the glovebox freezer (10 mg, 25%). Anal. Calcd. for $C_{48}H_{32}BF_{10}P_2Rh$: C, 59.2; H, 3.3. Found: C, 59.1; H, 3.5. However, and despite the values obtained for EA based on the small crop of crystalline material obtained, the high instability of this species prevented us from recording NMR data of the pure compound.

^{1}H NMR (500 MHz, C_6D_6, 25 °C) δ: 7.42 (m, 8H, *o*-Ph), 7.27 to 7.16 (m, 4H, *o*-Ph,), 7.00 (m, 12H, *m*-Ph, *p*-Ph), 6.90 to 6.82 (m, 6H, *m*-Ph), −1.23 (m, 1H, BH), −16.33 (m, 1H, RhH). ^{11}B{^{1}H} NMR (128 MHz, C_6D_6, 25 °C) δ: −1.2 (br). ^{13}C{^{1}H} NMR (125 MHz, C_6D_6, 25 °C) δ: 145.5 (dm, $^{1}J_{CF}$ = 245 Hz, *o*- C_6F_5), 140.5 (*ipso*-Ph_3), 139.2 (dm, $^{1}J_{CF}$ = 239 Hz, *p*-C_6F_5), 136.3 (dm, $^{1}J_{CF}$ = 236 Hz, *m*- C_6F_5), 134.0 (*o*-Ph_3), 133.7 (*m*-Ph_3), 130.2 (*p*-Ph_3), 128.4 (*o*-Ph_3). ^{19}F{^{1}H} NMR (471 MHz, C_6D_6, 25 °C) δ: −164.1 (m, *p*-C_6F_5), −160.8 (m, *m*-C_6F_5), −129.3 (br, *o*-C_6F_5). ^{31}P{^{1}H} NMR (162 MHz, C_6D_6, 25 °C) δ: 38.2 (d, $^{1}J_{PRh}$ = 115 Hz).

NMR spectra

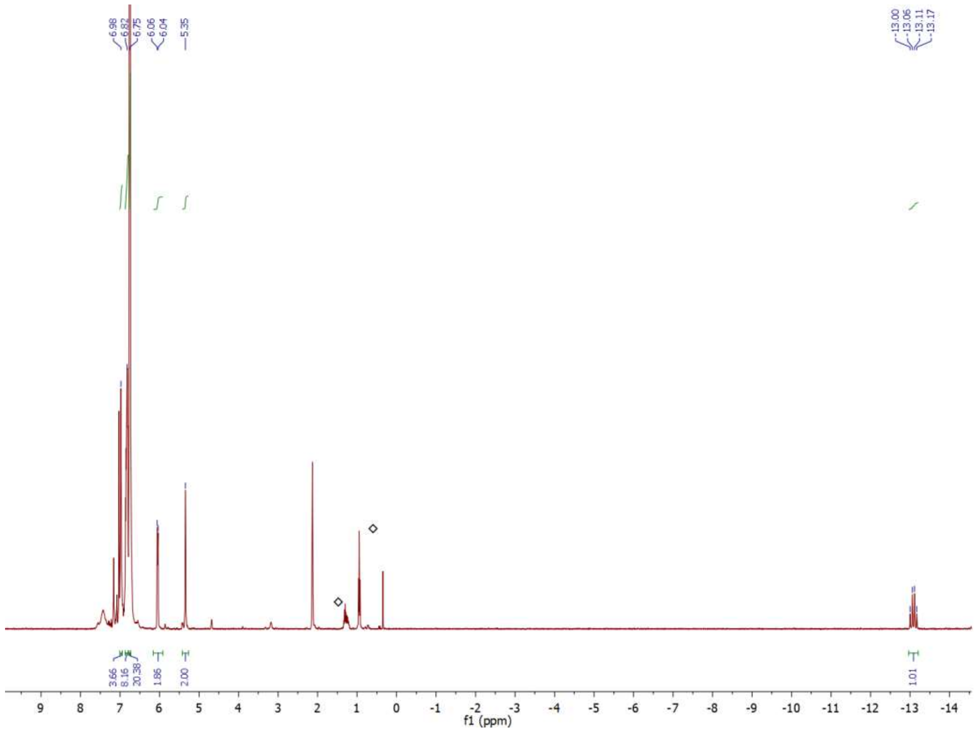

Figure S1. ^{1}H NMR of complex **2a**. Signals labeled with ◊ correspond to pentane.

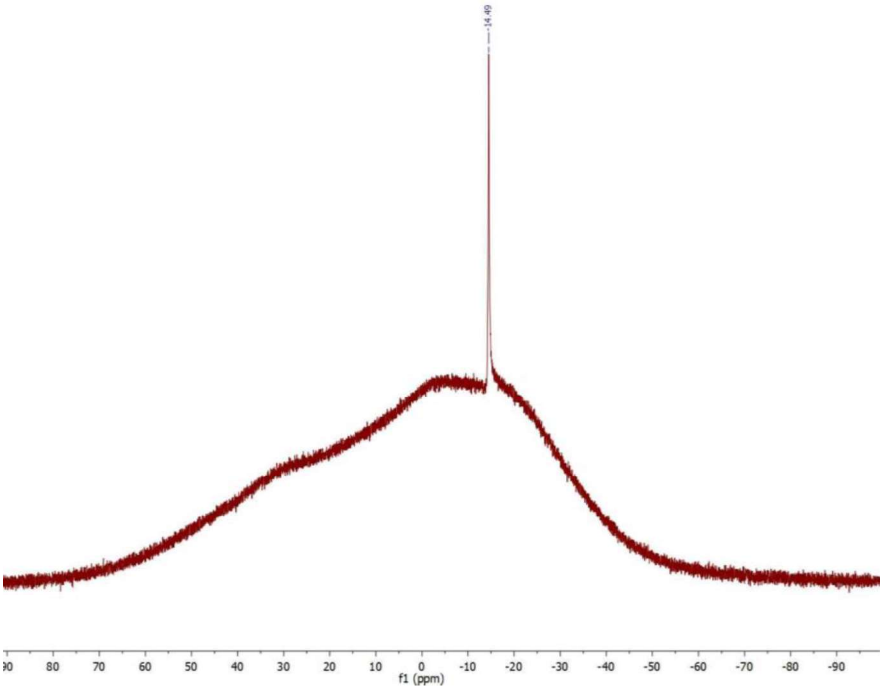

Figure S2. $^{11}B\{^{1}H\}$ NMR of complex **2a**.

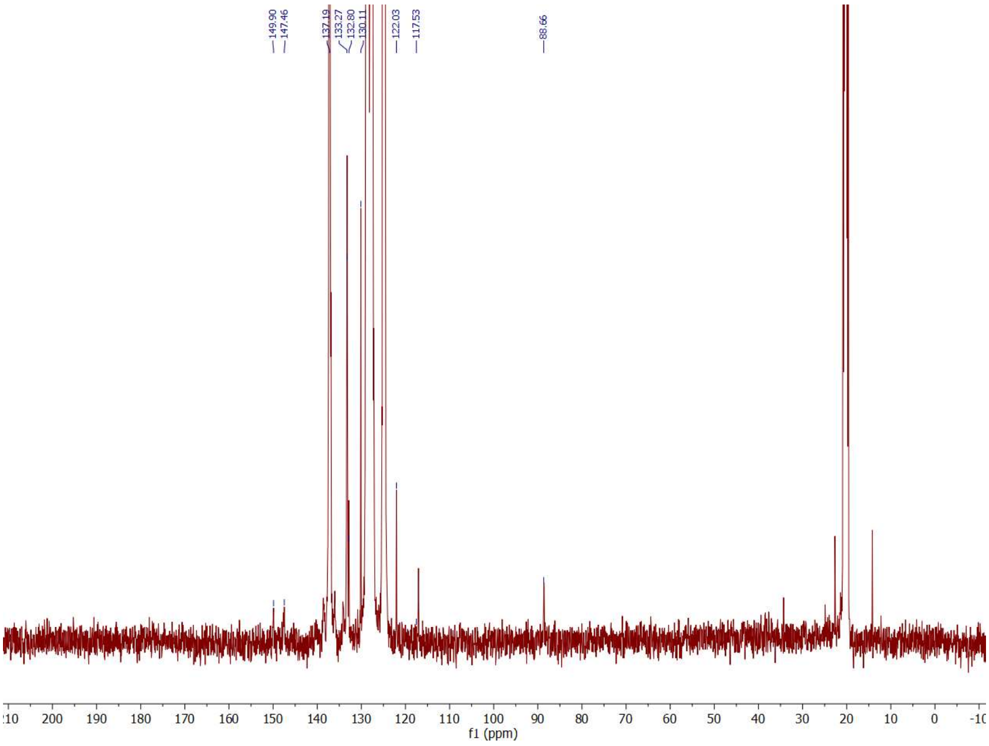

Figure S3. $^{13}C\{^{1}H\}$ NMR of complex **2a**.

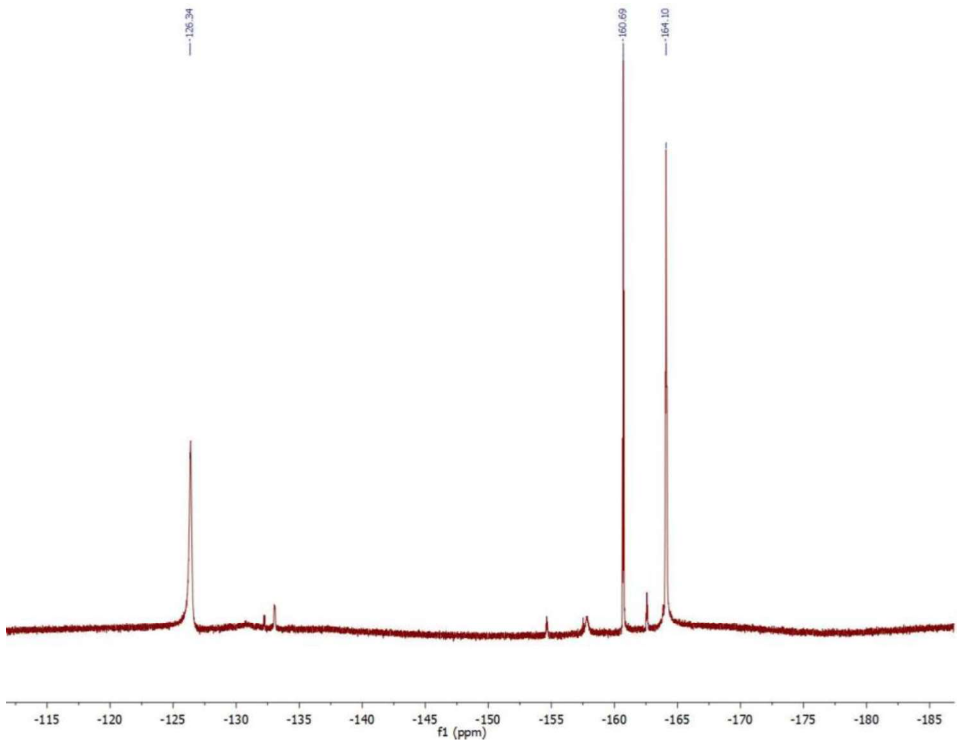

Figure S4. $^{19}F\{^{1}H\}$ NMR of complex **2a**.

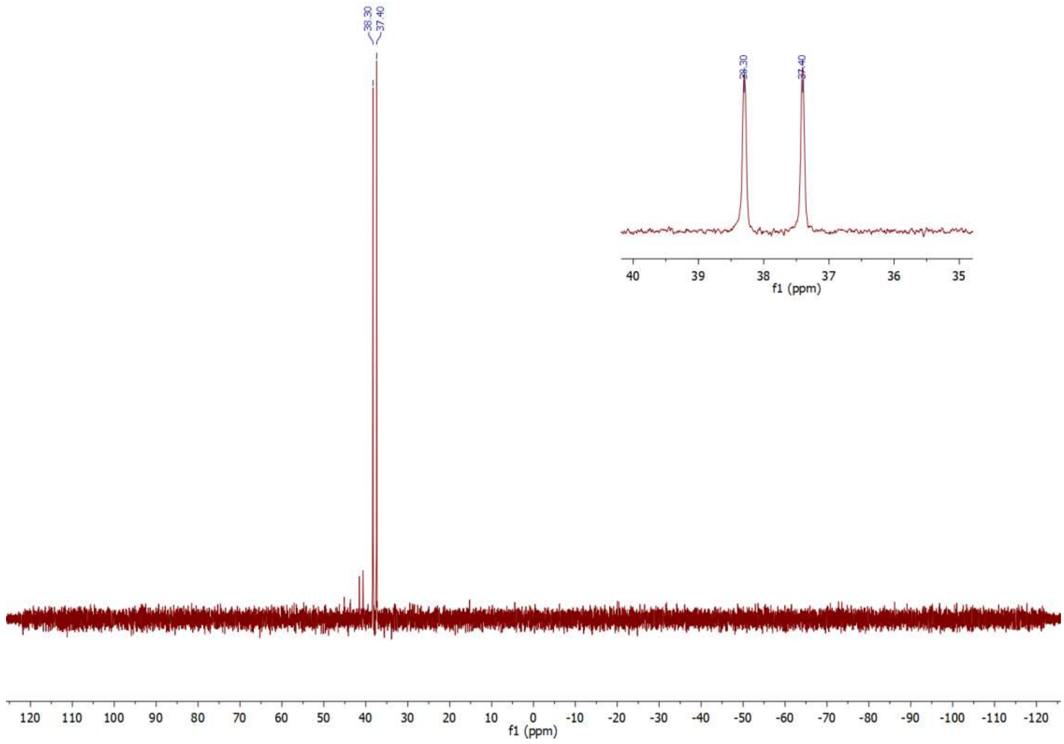

Figure S5. $^{31}P\{^{1}H\}$ NMR of complex **2a**.

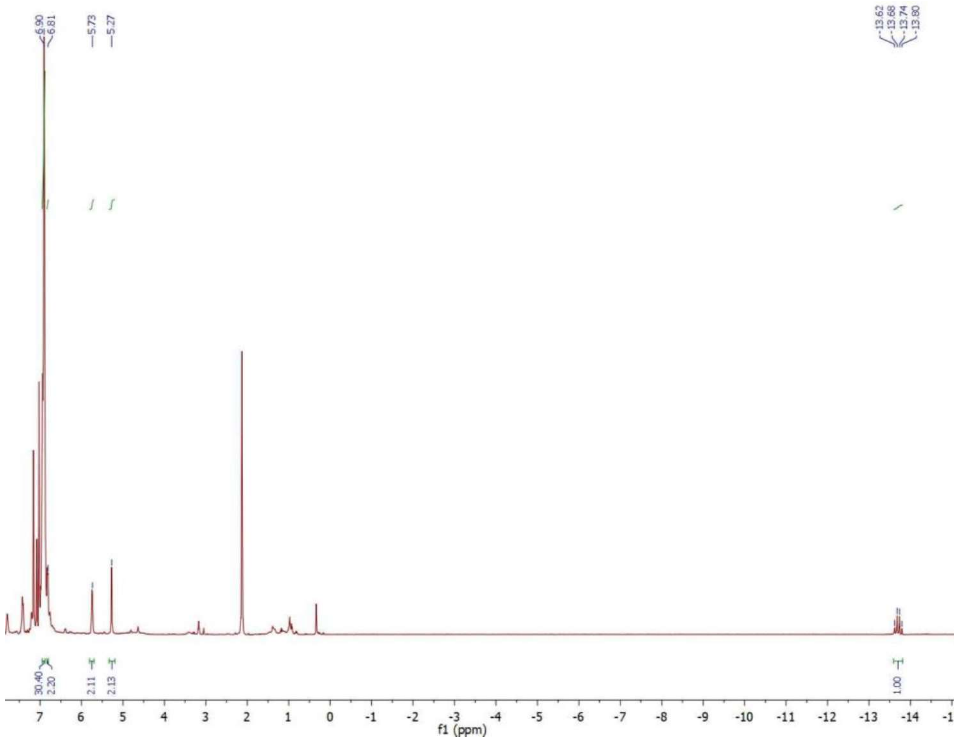

Figure S6. ^{1}H NMR of complex **2b**.

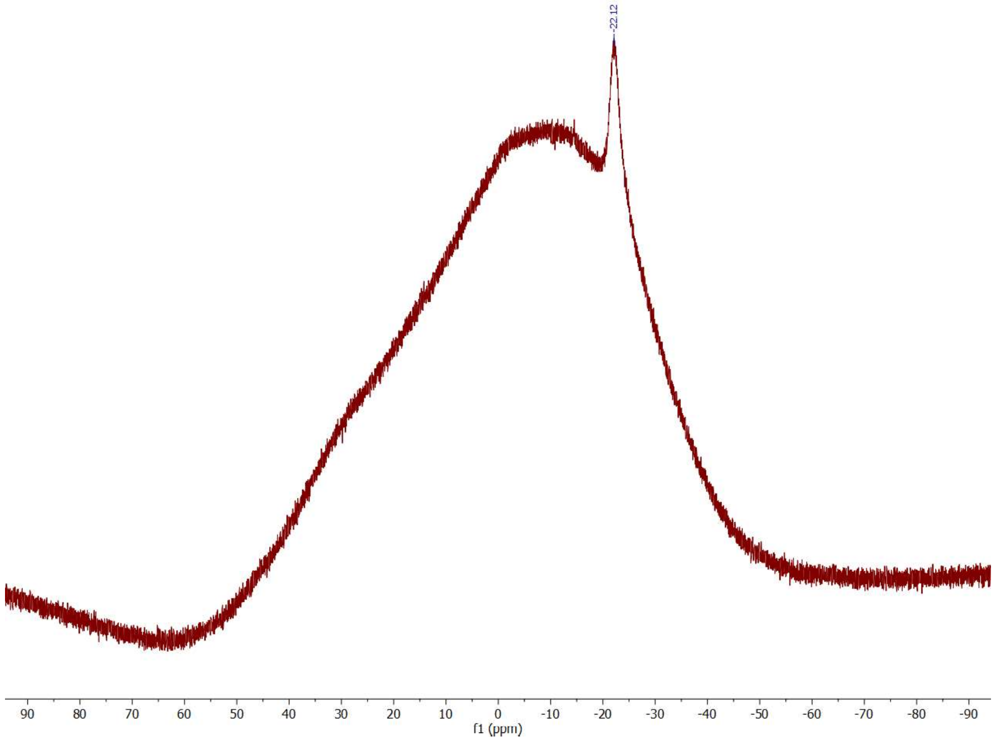

Figure S7. $^{11}B\{^{1}H\}$ NMR of complex **2b**.

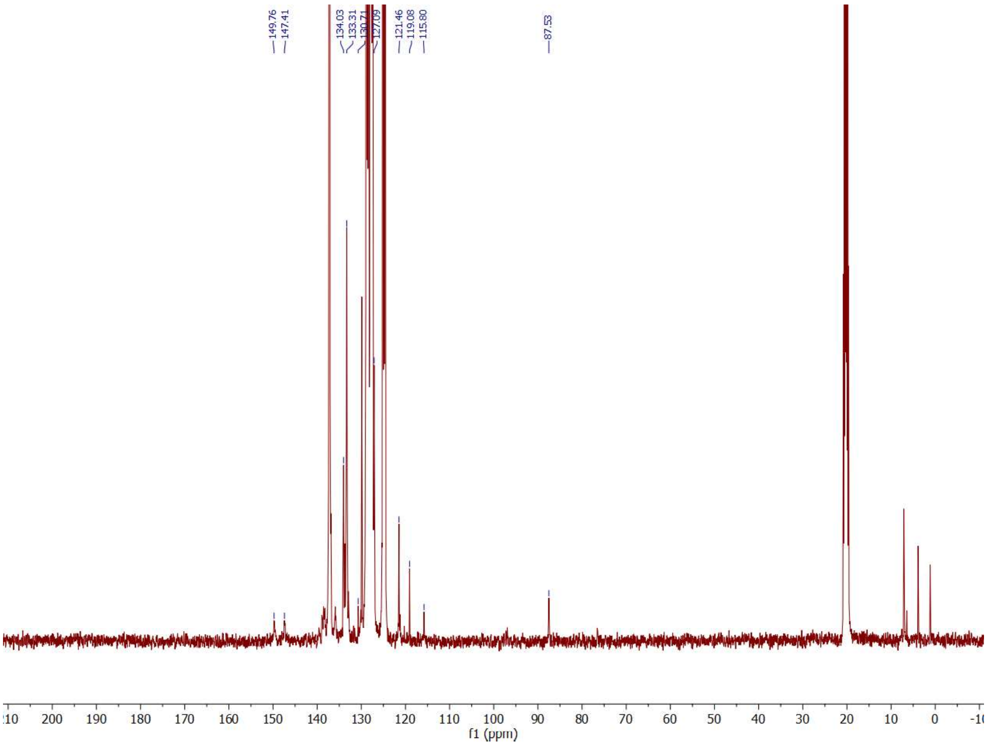

Figure S8. $^{13}C\{^{1}H\}$ NMR of complex **2b**.

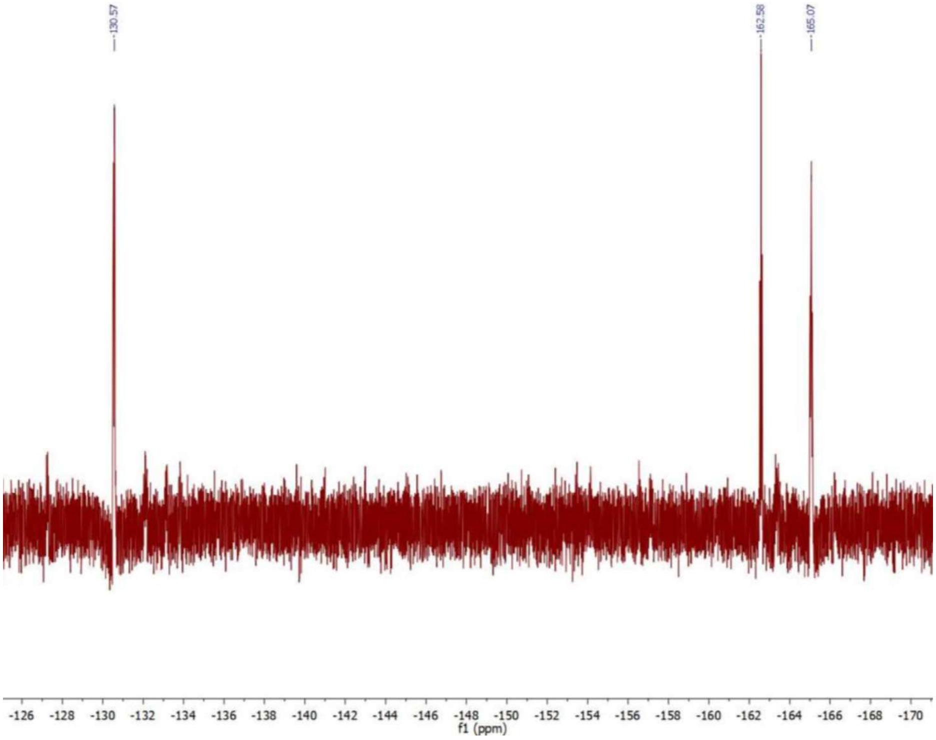

Figure S9. $^{19}F\{^{1}H\}$ NMR of complex **2b**.

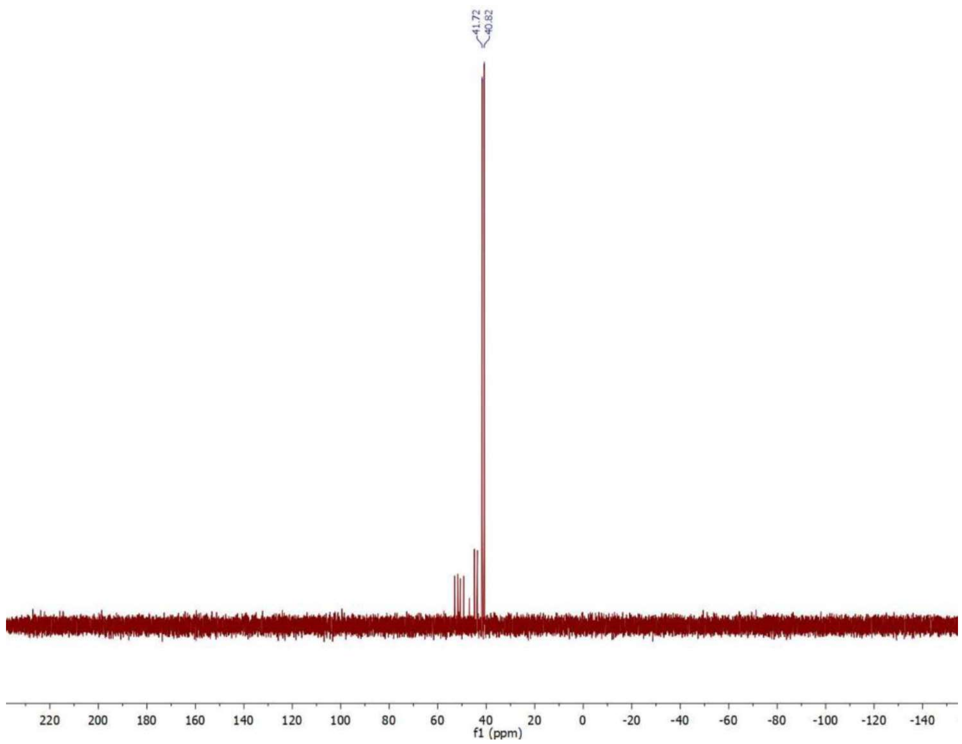

Figure S10. $^{31}P\{^{1}H\}$ NMR of complex **2b**.

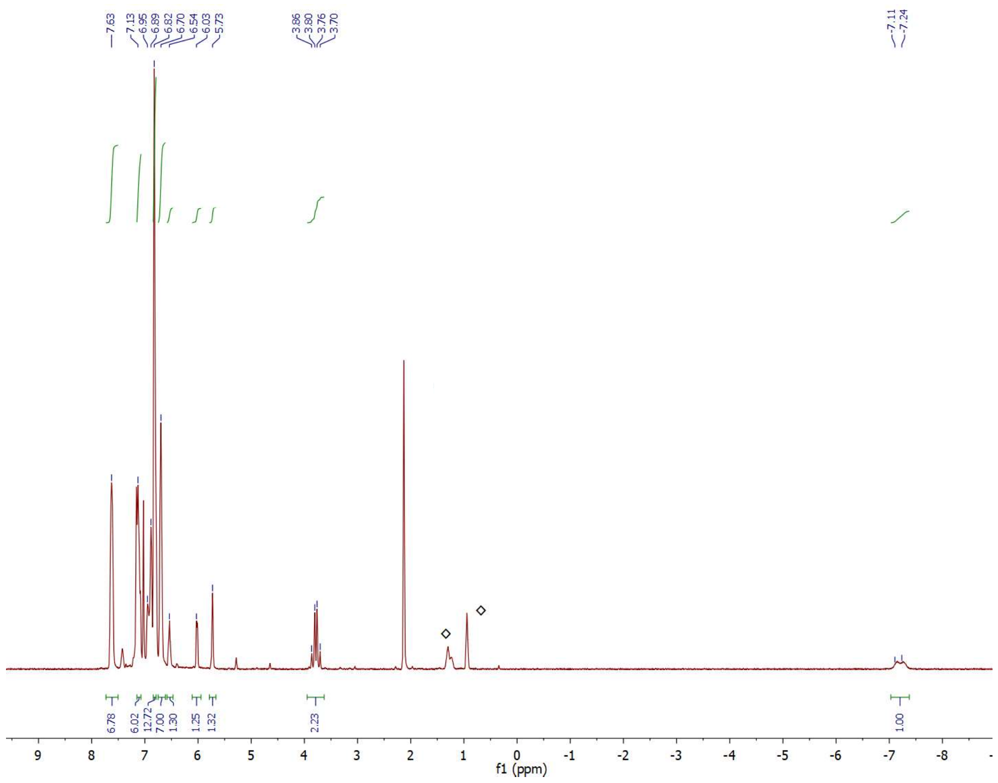

Figure S11. ^{1}H NMR of complex **3**. Signals labeled with ◊ correspond to pentane.

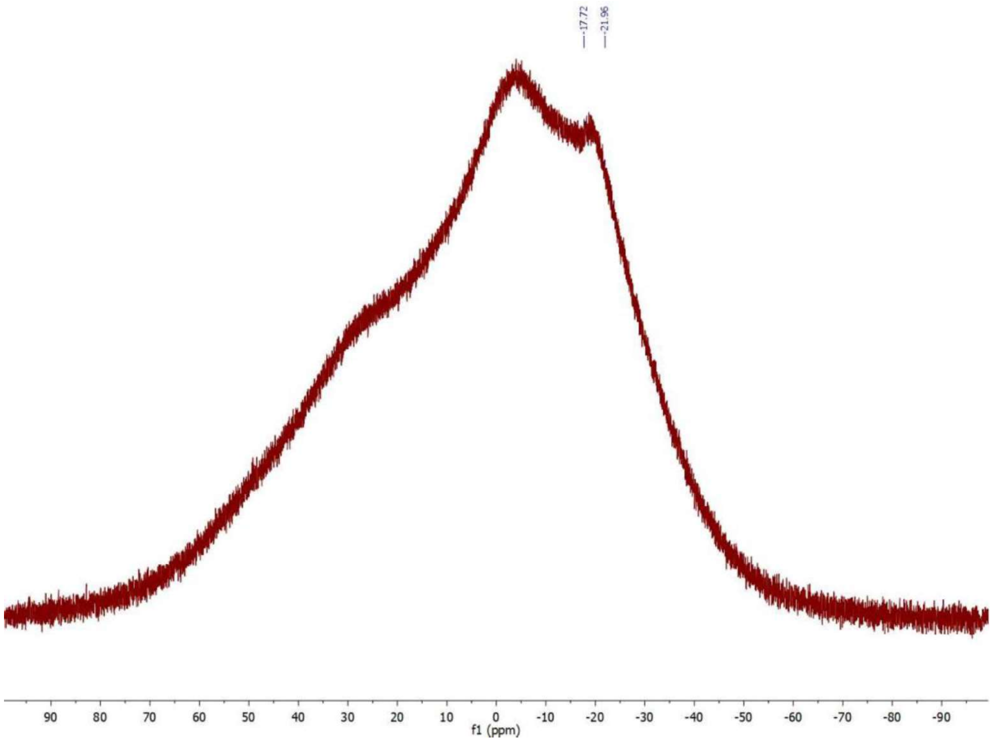

Figure S12. $^{11}B\{^{1}H\}$ NMR of complex **3**.

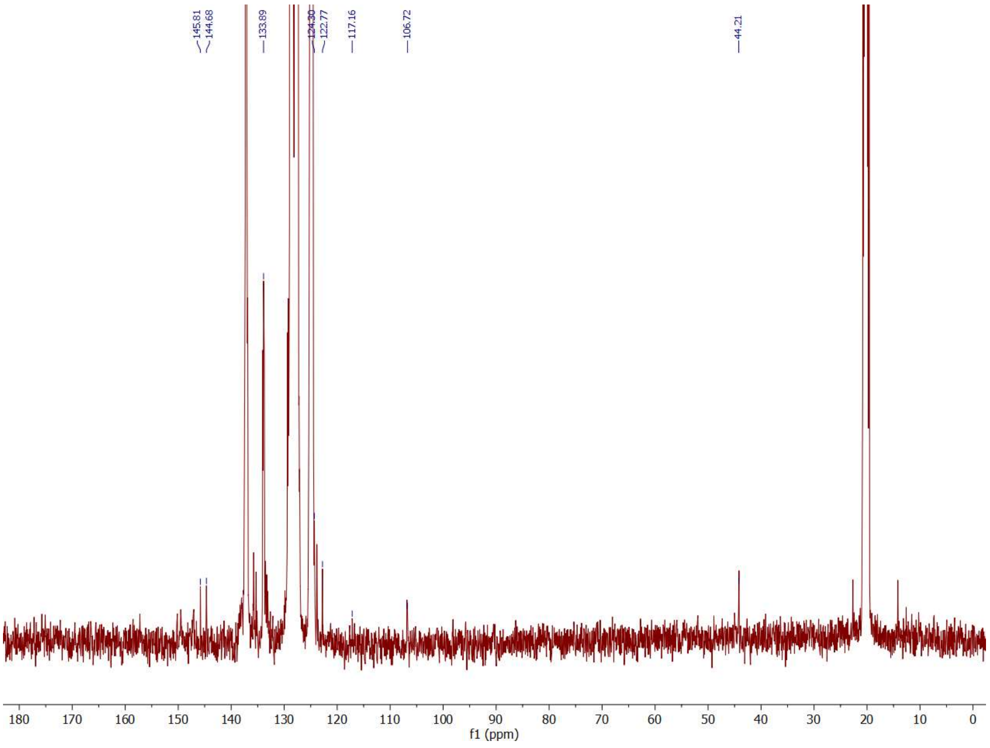

Figure S13. $^{13}C\{^{1}H\}$ NMR of complex **3**.

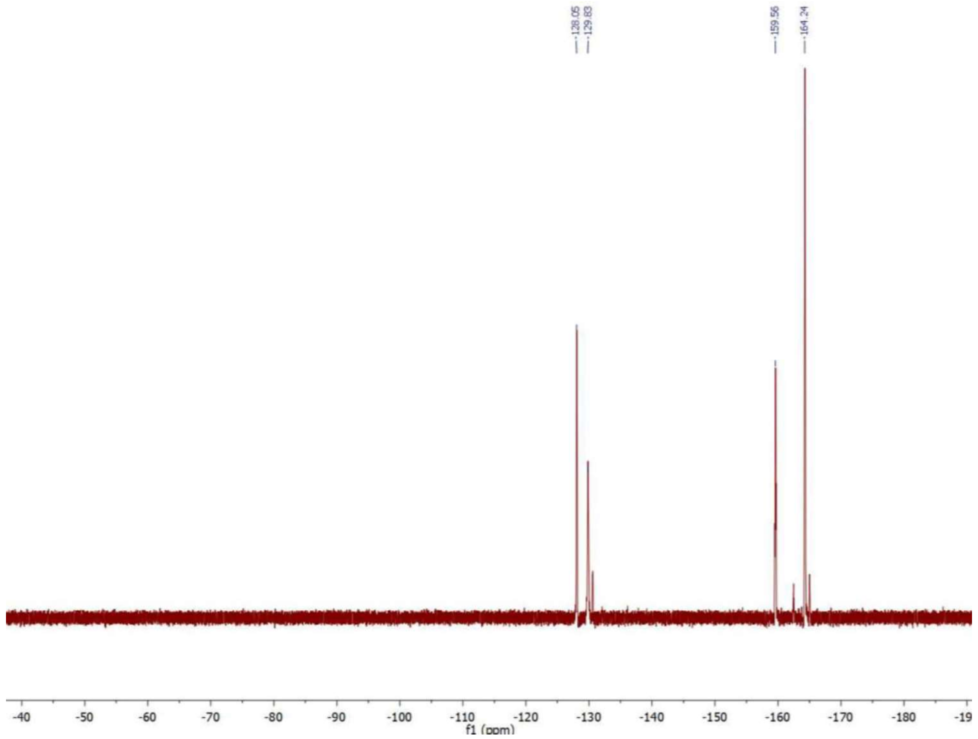

Figure S14. $^{19}F\{^{1}H\}$ NMR of complex **3**.

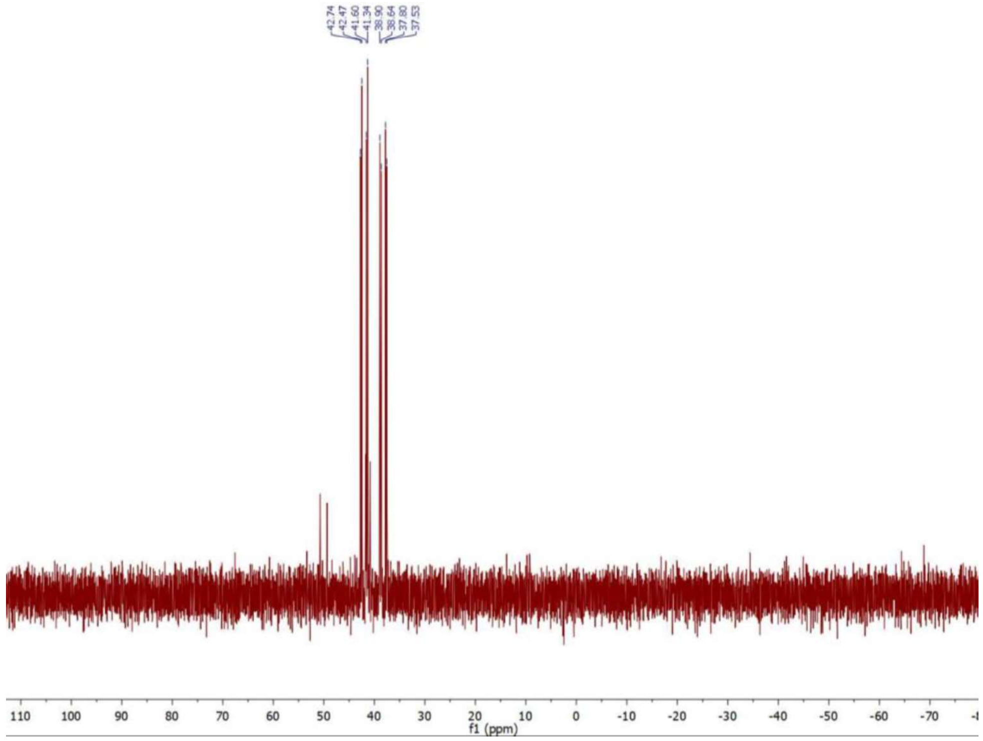

Figure S15. $^{31}P\{^{1}H\}$ NMR of complex **3**.

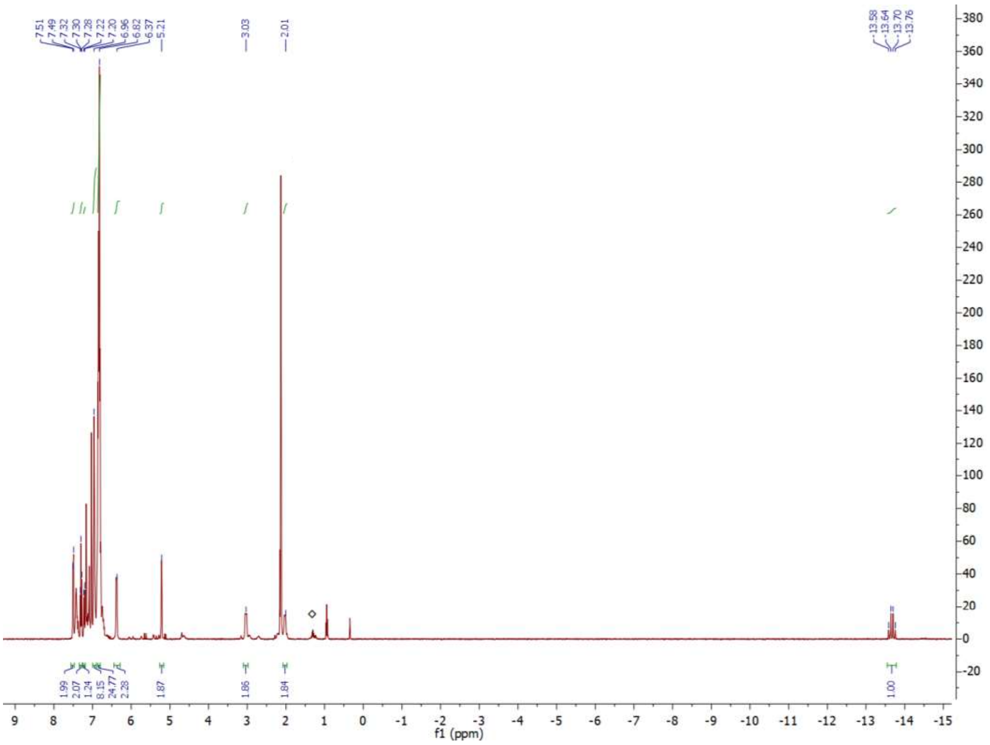

Figure S16. ^{1}H NMR of complex **4**. Signals labeled with ◊ correspond to pentane.

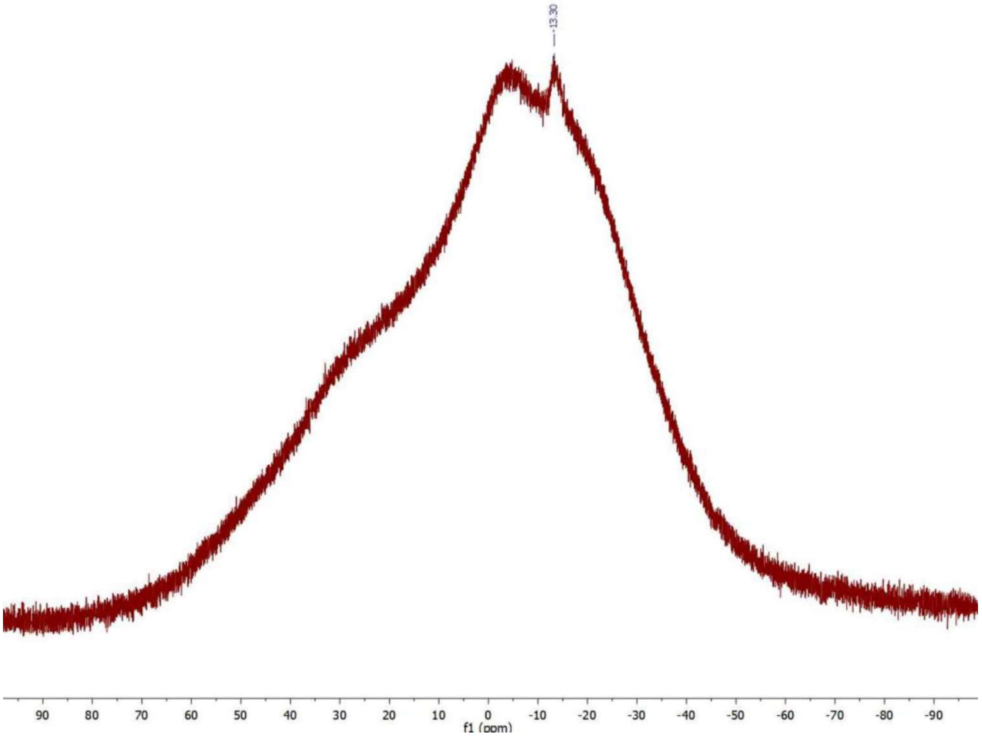

Figure S17. $^{11}B\{^{1}H\}$ NMR of complex **4**.

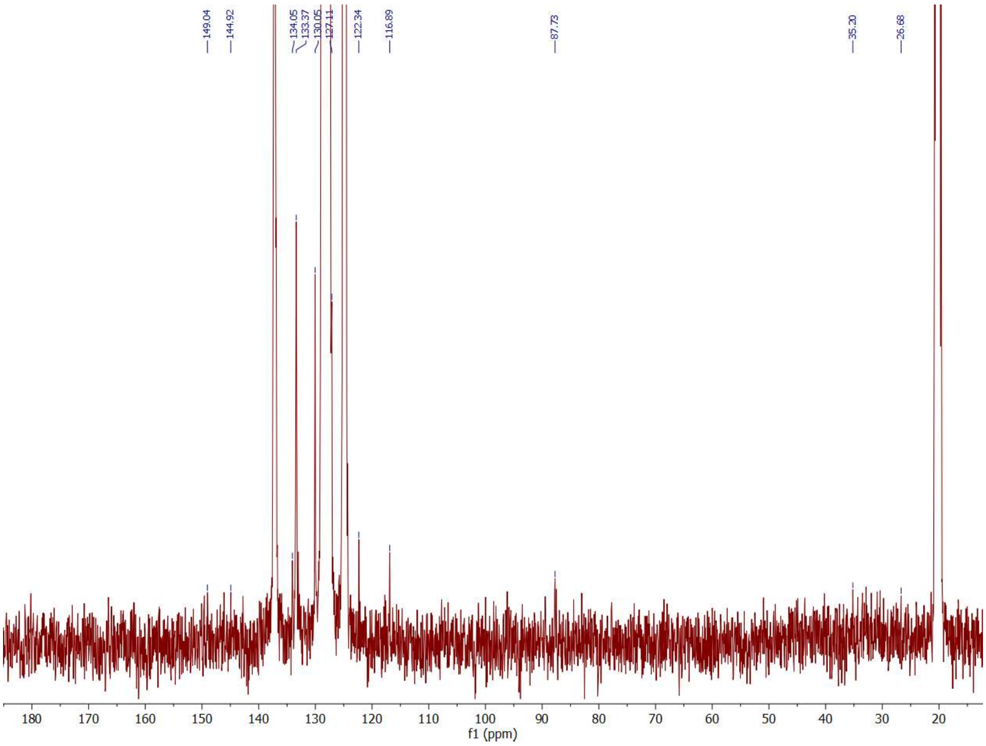

Figure S18. $^{13}C\{^{1}H\}$ NMR of complex **4**.

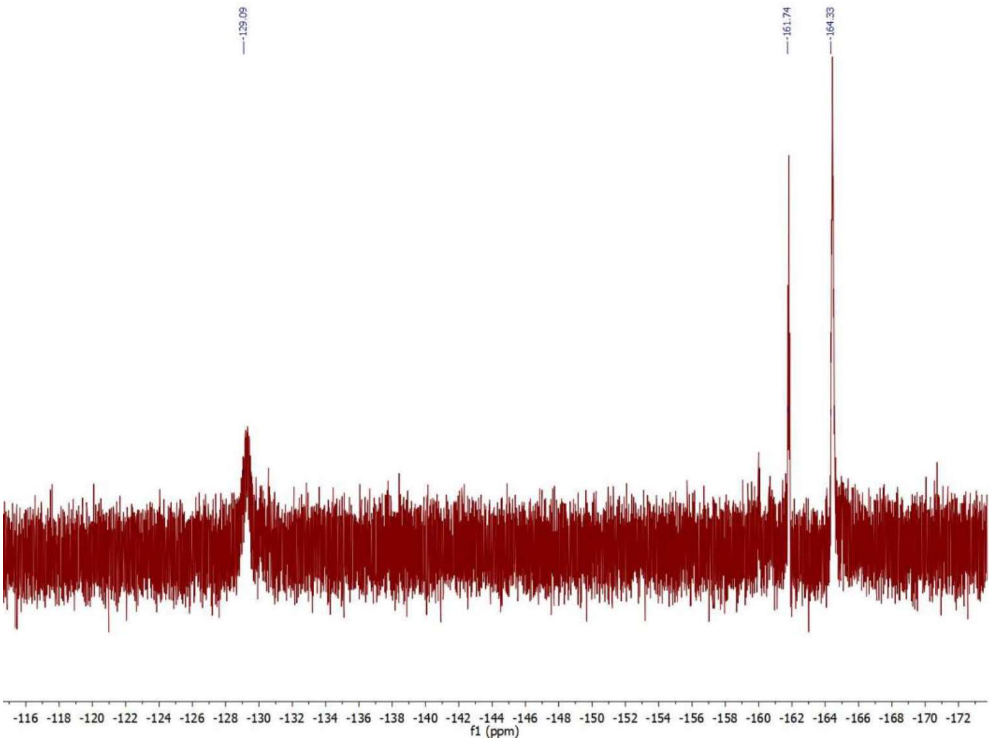

Figure S19. $^{19}F\{^{1}H\}$ NMR of complex **4**.

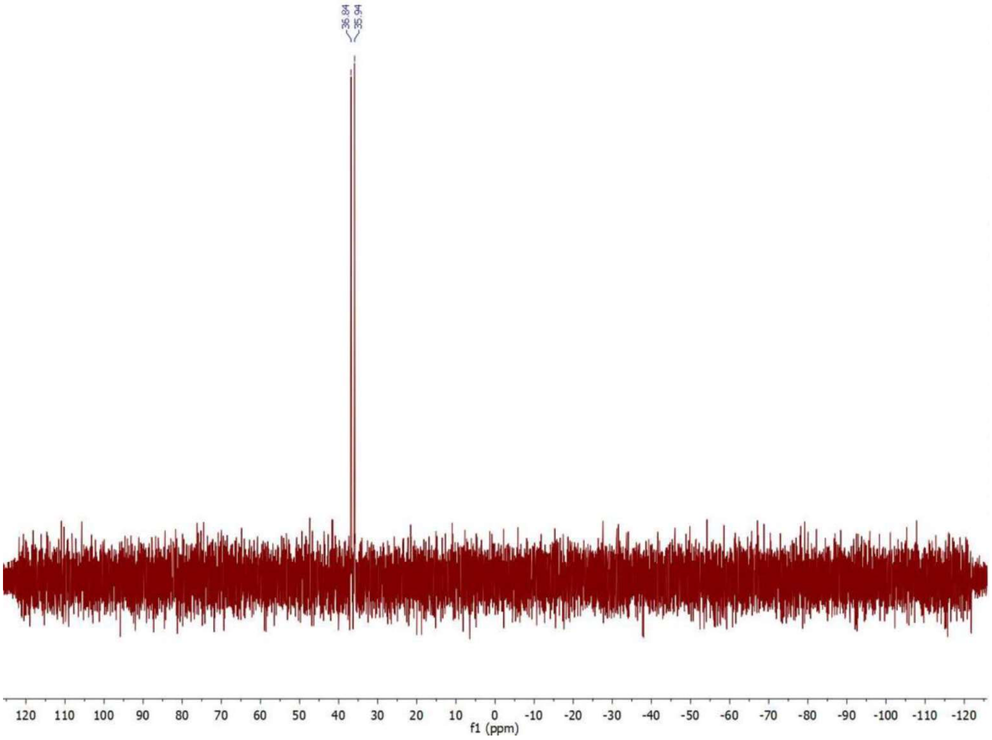

Figure S20. $^{31}P\{^1H\}$ NMR of complex **4**.

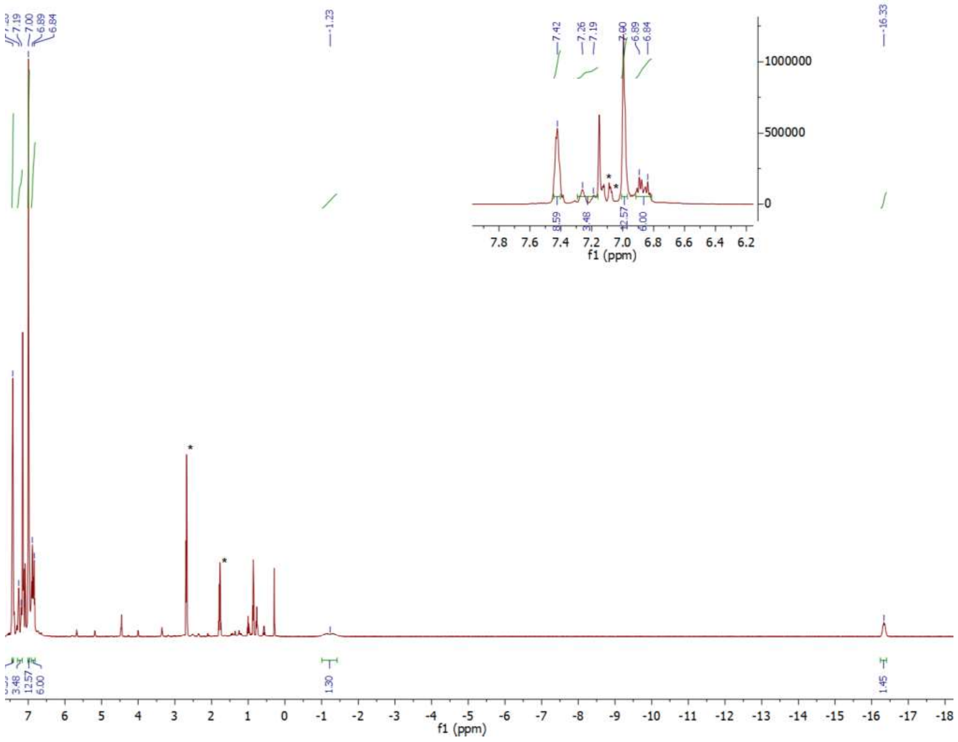

Figure S21. ^1H NMR of complex **5**. The symbol * denotes signals due to the released indane (C_9H_{10}).

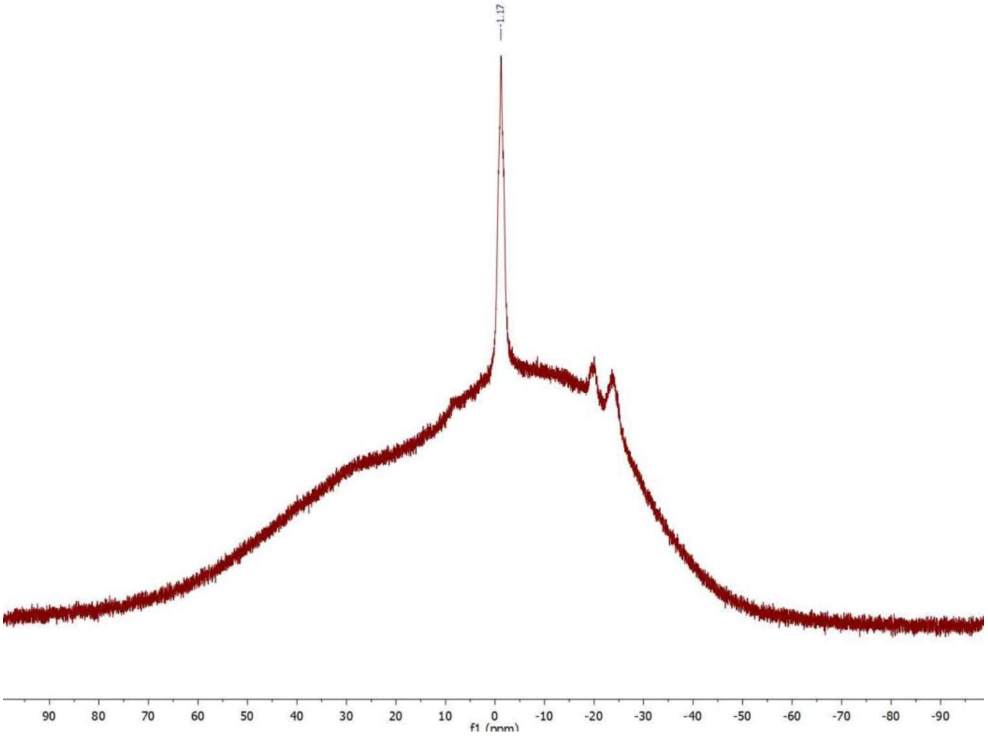

Figure S22. ${}^{11}B\{{}^{1}H\}$ NMR of complex **5**. Broad minor signals at −20 and −24 ppm are attributed to hydroborate salts.[3]

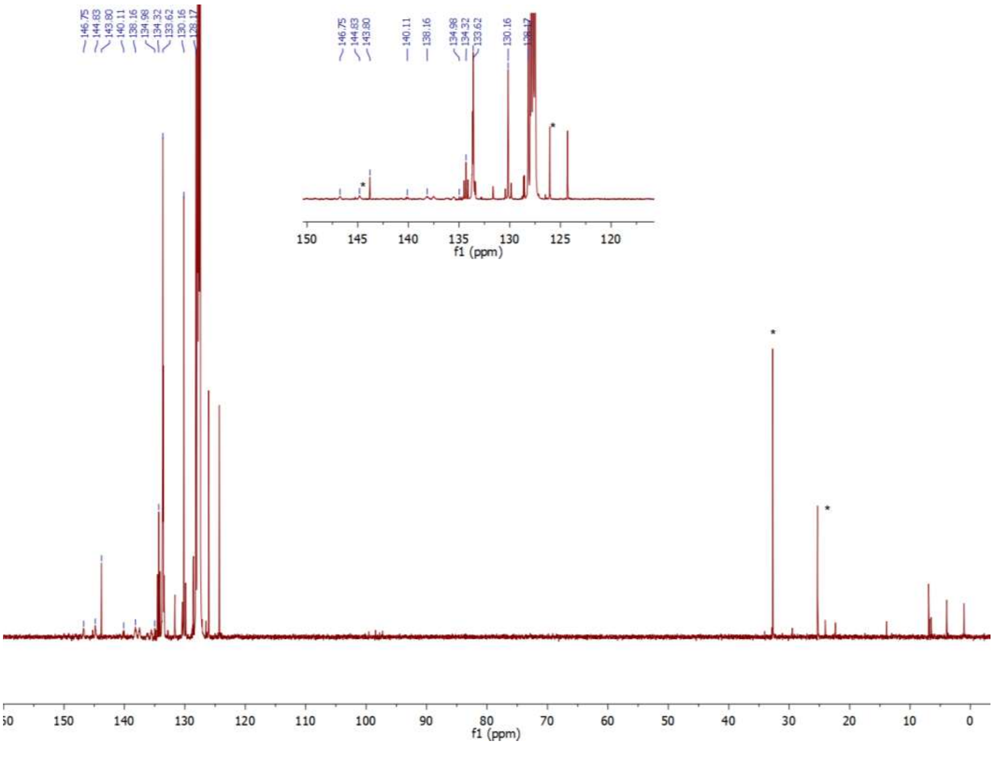

Figure S23. ${}^{13}C\{{}^{1}H\}$ NMR of complex **5**. The symbol * denotes signals due to the released indane (C_9H_{10}).

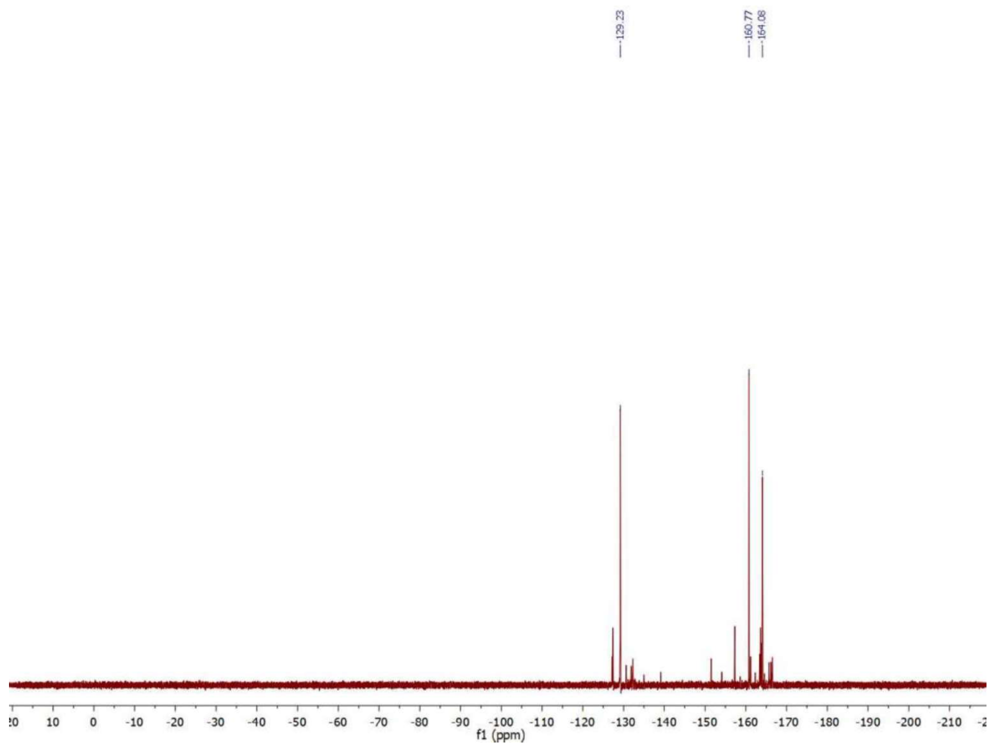

Figure S24. $^{19}F\{^{1}H\}$ NMR of complex **5**. Most minor signals are attributed to hydroborate salts.[3]

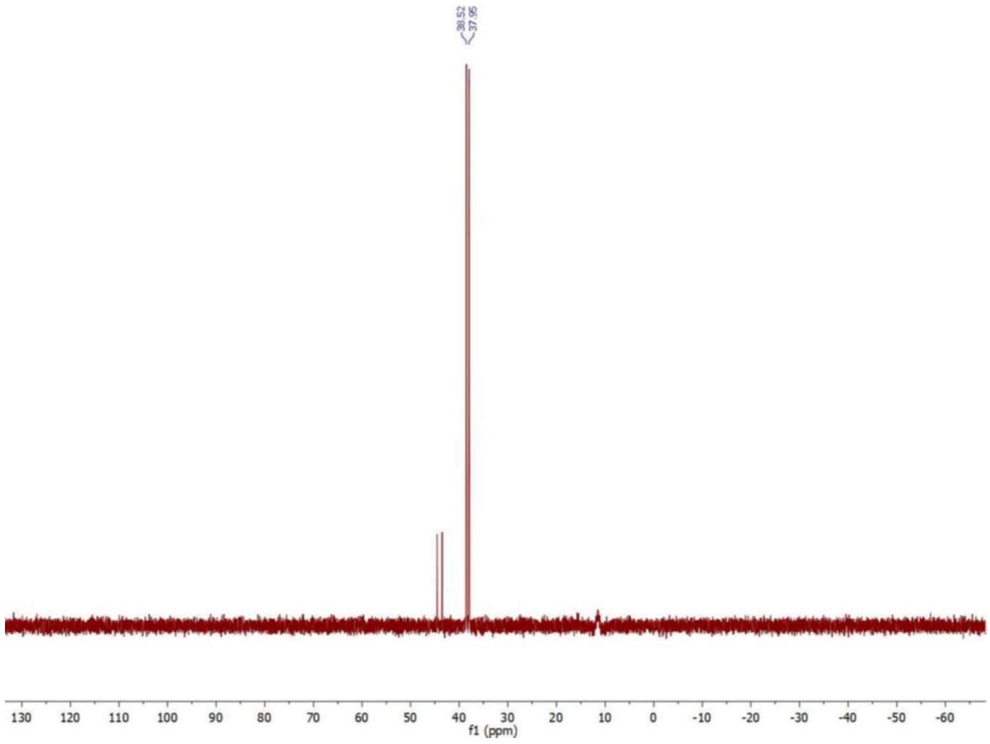

Figure S25. $^{31}P\{^{1}H\}$ NMR of complex **5**.

Crystal structure determinations

Crystallographic details. Low-temperature diffraction data were collected on a D8 Quest APEX-III single crystal diffractometer with a Photon III detector and an IμS 3.0 microfocus X-ray source (**2a, 2b, 3, 4 and 5**) at the Instituto de Investigaciones Químicas, Sevilla. Data were collected by means of ω and φ scans using monochromatic radiation λ(Mo Kα1) = 0.71073 Å. The diffraction images collected were processed and scaled using APEX-III software. Using Olex2[4], the structures **2a** and **2b** with olex2.solve and the structures **3**, **4** and **5** with SHELXT. All were refined against F^2 on all data by full-matrix least squares with SHELXL.[5] All non-hydrogen atoms were refined anisotropically. Hydrogen atoms were included in the model at geometrically calculated positions and refined using a riding model. The isotropic displacement parameters of all hydrogen atoms were fixed to 1.2 times the U value of the atoms to which they are linked (1.5 times for methyl groups). An exception is the hydrogens H and H1 bonded to Rh and B, respectively, in structure **7**. They were located from different Fourier maps and refined isotopically.

In three (**2b**, **3** and **5**) of these reported structures, the scattering contributions of disordered solvent molecules were removed with the solvent mask procedure implemented in Olex2.

A summary of the fundamental crystal and refinement data is given in Table S1. Atomic coordinates, anisotropic displacement parameters and bond lengths and angles can be found in the cif files, which have been deposited in the Cambridge Crystallographic Data Centre with no. 2239975, 2239977, 2239979, 2239980, 2239981. These data can be obtained free of charge from The Cambridge Crystallographic Data Centre via www.ccdc.cam.ac.uk/data_request/cif.

Table S1. Crystal data and structure refinement for compounds **2a**, **2b**, **3**, **4** and **5**.

	**2a**	**2b**	**3**	**4**	**5**
formula	$C_{63}H_{37}BF_{15}P_2Rh$, $+2.5xC_6H_6$	$C_{57}H_{38}BF_{10}P_2Rh$	$C_{57}H_{38}BF_{10}P_2Rh$	$C_{65}H_{45}BF_{10}P_2Rh$	$C_{48}H_{32}BF_{10}P_2Rh$
Fw	1449.85	1050.73	1088.53	1191.67	974.39
cryst.size, mm	0.19 × 0.13 × 0.12	0.15 × 0.11× 0.09	0.18 × 0.15 × 0.12	0.23 × 0.14 × 0.10	0.15 × 0.12× 0.10
crystal system	Triclinic	Triclinic	Triclinic	Triclinic	Triclinic
space group	*P-1*	*P-1*	*P-1*	*P-1*	*P-1*
a, Å	13.5421(8)	12.4932(7)	11.9503(5)	13.4147(7)	10.5086(8)
b, Å	13.6952(7)	14.5721(7)	16.2587(7)	14.3442(8)	12.6977(9)
c, Å	19.1317(12)	15.3049(8)	17.3192(7	18.7013(10)	17.3473(13)
α, deg	84.8277(18)	92.693(2)	113.5454(13)	72.746(2)	98.977(3)
β, deg	75.114(2)	102.918(2)	104.8225(13)	73.105(2)	99.247(3)
γ, deg	68.2511(16)	100.272(2)	98.0361(14	89.419(2)	98.137(3)
V, Å^3	3185.0(3)	2661.3(2)	98.0361(14	3276.9(3)	2223.5(3)
T, K	193	193	193	193	193
Z	2	2	2	2	2
ρ_{calc}, g cm^{-3}	1.512	1.311	1.260	1.208	1.455
μ, mm^{-1} (MoKα)	0.409	0.450	0.419	0.373	0.531
F(000)	1470	1025	1100	m_exptl_crystal_F_000	980
absorption corrections	multi-scan, 0.63 – 0.75	multi-scan, 0.35 – 0.75	multi-scan, 0.67 – 0.75	multi-scan, 0.69 – 0.75	multi-scan, 0.68– 0.75
θ range, deg	2.203 – 25.013	1.886 – 28.331	1.988– 28.277	2.084 – 28.281	1.993 – 29.558
no. of rflns measd	43950	94656	134471	110761	36015
R_{int}	0.0978	0.0558	0.0621	0.0570	0.0502
no. of rflns unique	11019	13220	14226	16233	11673
no. of params / restraints	878/ 30	674/ 0	656/ 0	815 / 0	567/ 0
R_1 ($I > 2σ(I)$) [a]	0.0441	0.0679	0.0359	0.0379	0.0636
R_1 (all data)	0.0761	0.0871	0.0564	0.0518	0.0836
wR_2 ($I > 2σ(I)$)	0.0759	0.1988	0.0766	0.0988	0.1740
wR_2 (all data)	0.0856	0.2273	0.0919	0.1059	0.1882
Diff.Fourier.peaks min/max, eÅ^{-3}	-0.630/ 0.615	-0.823 / 2.998	-0.781 / 1.020	-0.565/ 0.395	-0.863 / 1.625
CCDC number	2239975	2239979	2239981	2239980	2239977

NMR reaction monitoring of catalytic runs.

Figure 26 shows an example of reaction monitoring used to build the kinetic profiles represented in Figure 6 of the main text. In this case, complex **5** (0.0002 mmol) and styrene (0.004 mmol) were dissolved in C_6D_6 (0.2 mL) in a high-pressure NMR tube. The tube was freeze-pumped to remove the nitrogen gas and filled with 6 bar of H_2 pressure. The reaction was monitored by ^1H NMR spectroscopy at different times.

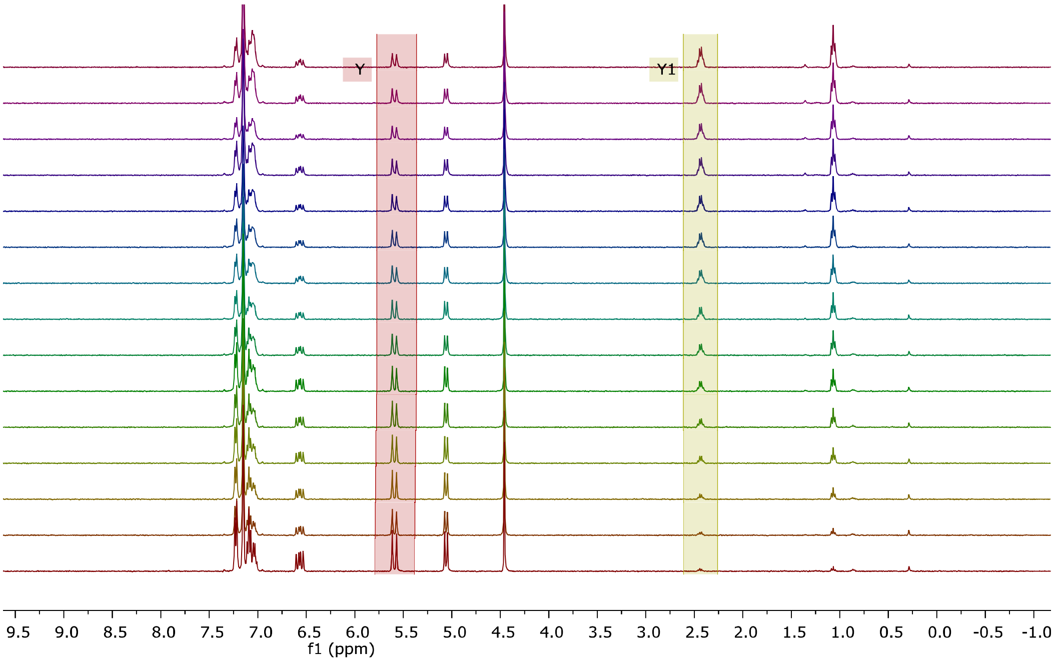

Figure S26. ^1H NMR monitoring of the catalytic hydrogenation of styrene. The proton signals of styrene are marked in red, and the proton signals of the hydrogenated styrene product are marked in yellow.

Exchange EXSY experiments for compound 5.

The spectroscopic characterization of **5** reveals the presence of two chemical exchange processes taking place in solution, more precisely the intramolecular exchange between the two hydrides and the intermolecular exchange between the Rh−H unit and free H_2 in solution. To investigate further these processes, we carried out 2D-exchange spectroscopy (EXSY) studies. Experimentally, complex **3** (0.138 mmol) was dissolved in C_6D_6 (0.2 mL) in a high-pressure NMR tube. The tube was freeze-pumped to remove the nitrogen gas, then filled with 6 bar of H_2 pressure. The solution was intermittently shaken to favour diffusion of dihydrogen into the solution over a period of 2 hours to assure full conversion towards **5.** Then the solution was monitored by NOESY (or EXSY) spectroscopy at different temperatures.

The intramolecular exchange between hydrides was observed only at higher temperatures, while the exchange of the rhodium hydride with free H_2 was more facile. Figures S27 and S28 contain representative examples of 2D-EXSY experiments at a mixing time where there is exchange (0.3 s) and when there is no exchange (0.005 s) for the intramolecular process. Similarly, Figures S30 and S31 are representative examples of analogous experiments associated to the intermolecular exchange with H_2.

These studies were done in the temperature interval between 50 and 65 ºC for the intramolecular exchange and from 0 to 25 °C for the exchange with H_2. The visible higher facility of the later exchange is in agreement with our Eyring analyses, which rendered values of $\Delta S^{\ddagger}$ = 2.03±0.11 cal/Kmol and $\Delta H^{\ddagger}$ = 18.8±0.8 kcal/mol, corresponding to $\Delta G^{\ddagger}_{298}$ of 18±3 kcal/mol. The intramolecular exchange is characterized by a higher $\Delta G^{\ddagger}_{298}$ of 28.2±0.5 kcal/mol, and associated $\Delta S^{\ddagger}$ and $\Delta H^{\ddagger}$ values of 3.0±0.1 cal/Kmol and 29.1±0.7 kcal/mol, respectively.

Intramolecular exchange between the two hydride ligands in complex 5.

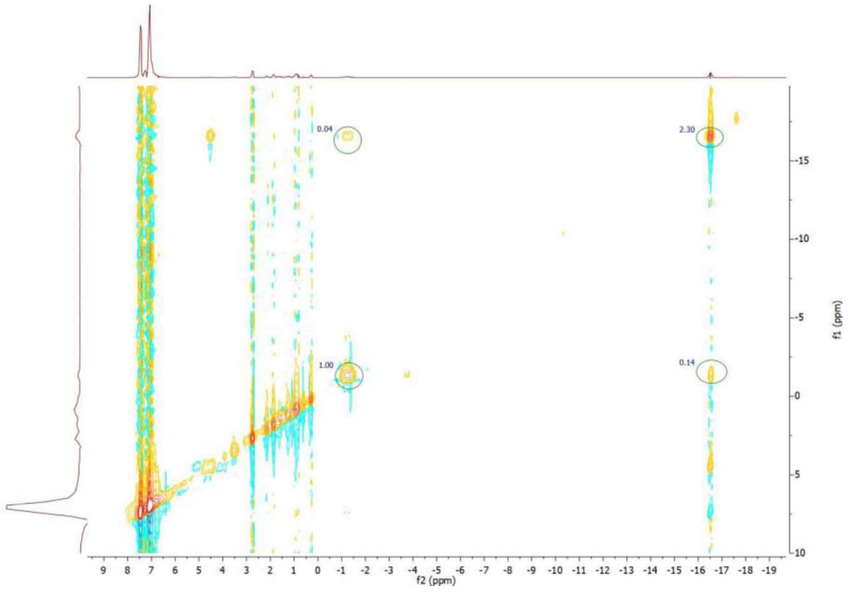

Figure S27. Example of NOESY experiment with mixing time of 0.3 s.

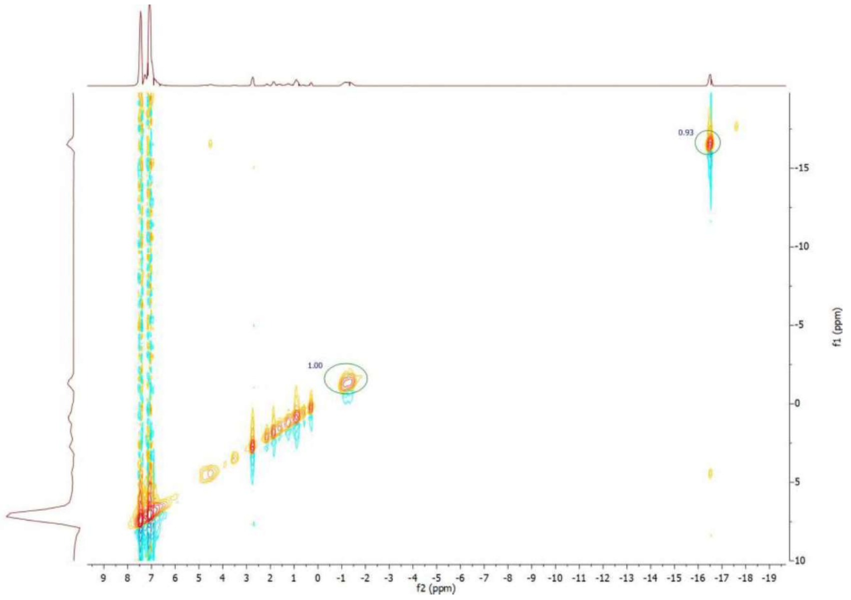

Figure S28. Example of NOESY experiment with mixing time of 0.005 s.

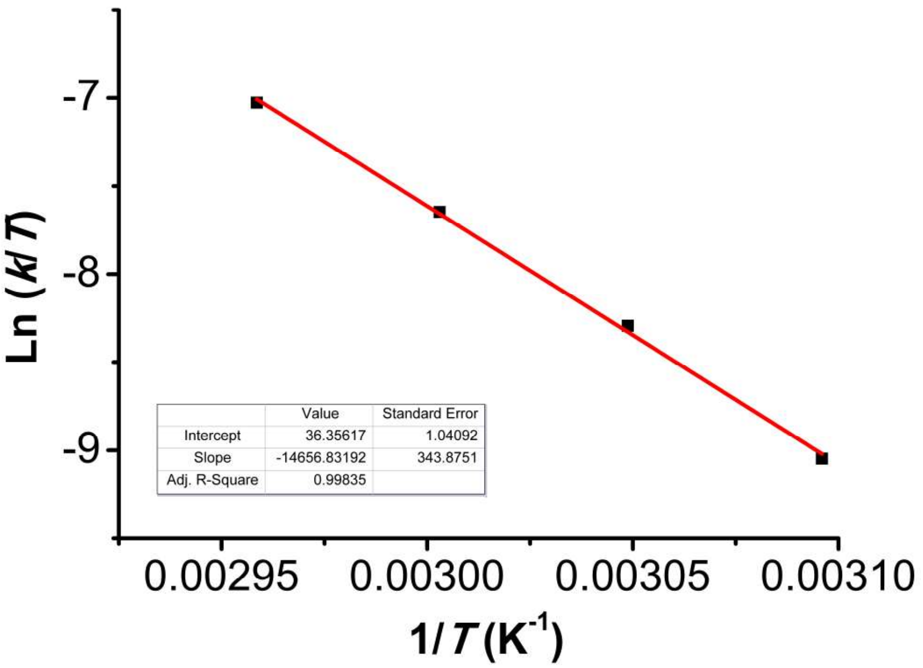

Figure S29. Eyring plot for the intramolecular exchange of the two hydrides in complex **5** in the temperature interval from 65 to 50 °C.

Intermolecular exchange between the Rh hydride in complex 5 and free H_2.

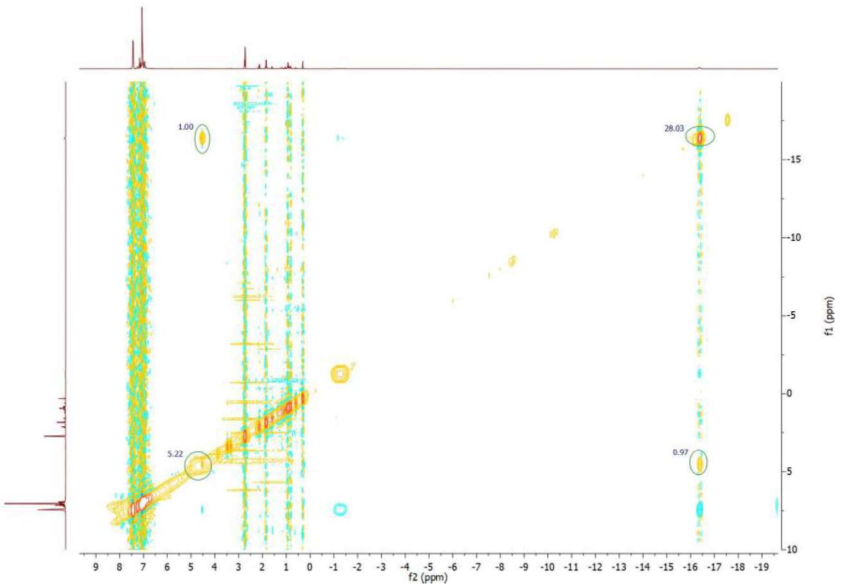

Figure S30. Example of NOESY experiment with mixing time of 0.3 s.

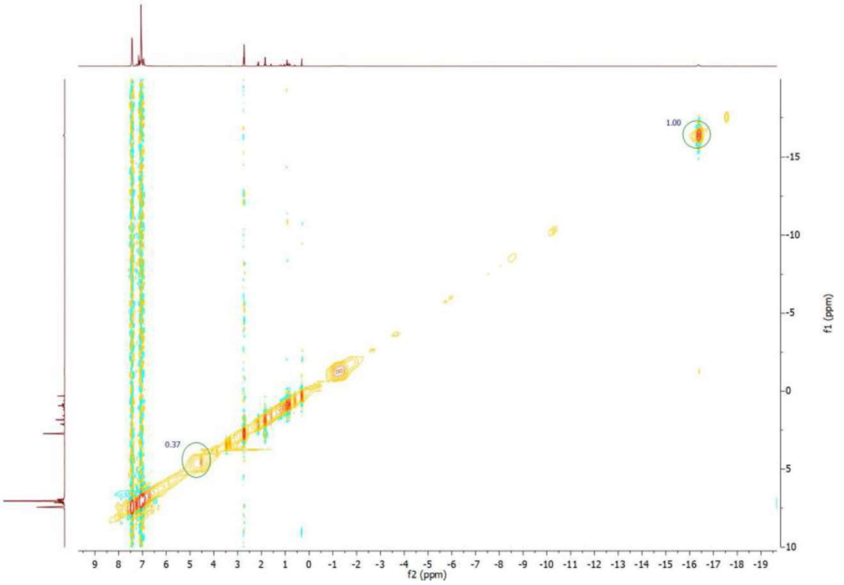

Figure S31. Example of NOESY experiment with mixing time of 0.005 s.

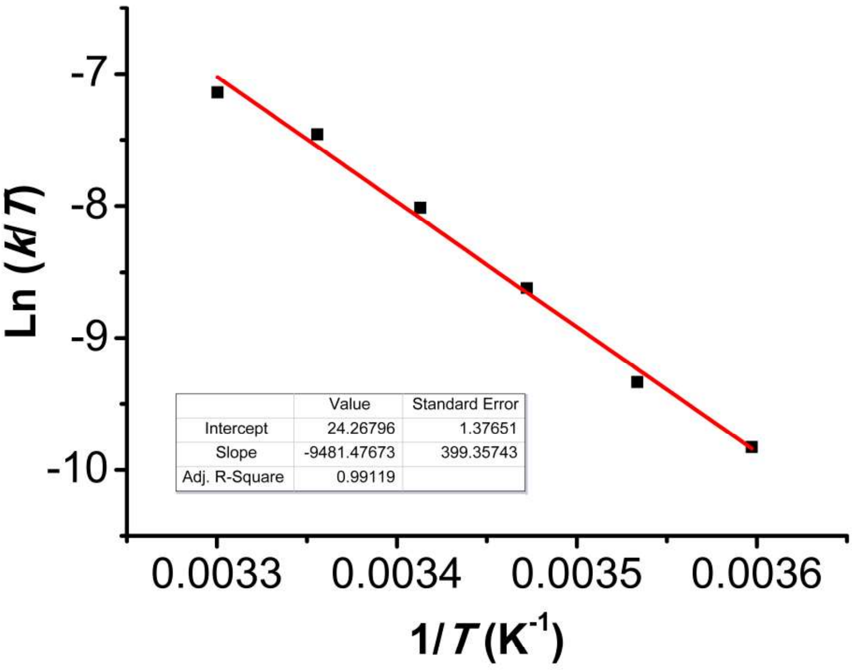

Figure S32. Eyring plot for the intermolecular exchange between the rhodium hydride in complex **5** and free H_2 from 25 to 0 ºC.

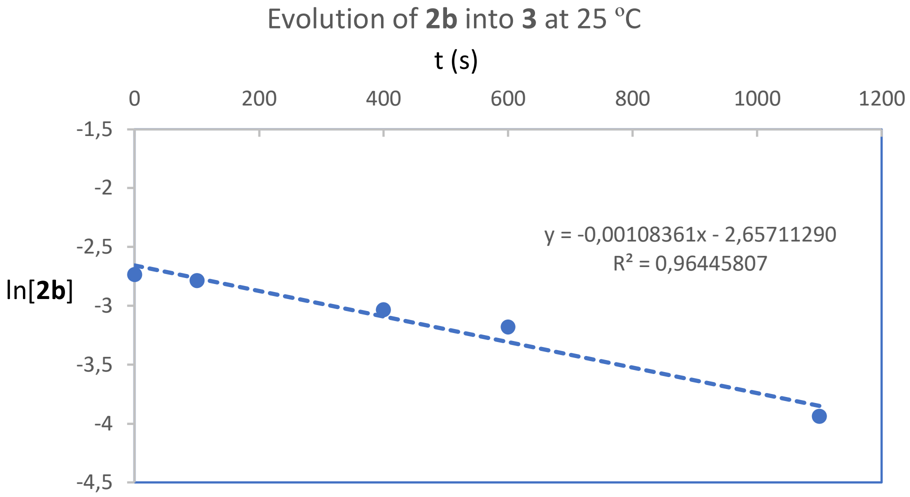

Figure S33. Pseudo-first order kinetic representation of the evolution of **2b** into **3** at 25 °C monitored by $^{31}P\{^1H\}$ NMR spectroscopy, corresponding to a ΔG_{298} of 21.5 kcal/mol.

Optimization of catalytic conditions using complex 3.

In a typical experiment, the reaction takes place either on a J. Young ampoule (for H_2 pressures up to 1 atm) or in a Fisher-Porter reactor (4 atm, H_2). In both cases, catalysis was carried out under strictly dry conditions. When the amount of precursors was easily weighable, complex **1** and $HB(C_6F_5)_2$ were dissolved in benzene to form complex **3** in situ and then the corresponding amount of styrene was added. The reaction mixture was then freeze-pumped to remove the nitrogen gas, filled with the corresponding H_2 pressure and placed at the corresponding temperature (25 – 60 °C). In cases where the amount of catalyst was too low to be weighed, aliquots from stock solutions in benzene were used instead. Substrate conversion was determined by ^1H NMR using hexamethylbenzene as an internal standard. Table S2 summarizes these results.

Table S2. Screening of conditions for the hydrogenation of styrene mediated by complex **3**.

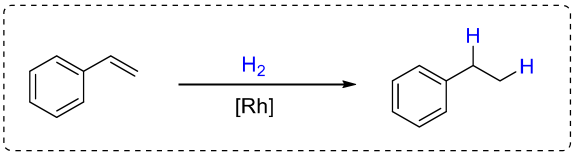

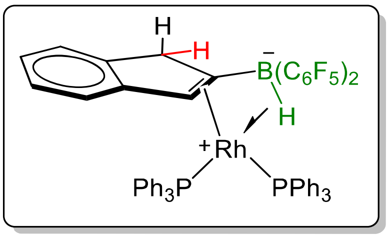

Entry	Rh (mol%)	H_2 (atm)	T (°C)	t (h)	Solvent	Additive	Yield (%)	TON	TOF (h^{-1})
1	1	0.5	25	3	Benzene	-	100	100	33
2	0.1	1	25	41	Benzene	-	100	1,000	24
3	0.1	1	25	16	Benzene	-	40	400	25
4	0.1	4	25	3	Benzene	-	100	1,000	333
5	0.01	1	25	70	Benzene	-	70	7,000	100
6	0.01	4	40	8	-	-	100	10,000	1,250
7	0.01	4	40	8	-	Hg	100	10,000	1,250
8	0.01	4	40	8	-	CS_2	0	0	0
9	0.01	4	40	8	-	CS_2[a]	35	3,500	437
10	0.01	4	40	8	-	CS_2 (10 %)	90	9,000	1,125
11	0.01	4	40	8	-	CS_2 (10 %)[a]	93	9,300	1,162
12	0.001	4	60	12	-	-	100	100,000	8,333
13	1 ppm	4	60	17	-	-	100	1,000,000	58,823
14	0.1 ppm	4	60	20	-	-	25	2,500,000	125,000

[a]In these poisoning experiments CS_2 was added after around two hours of reaction, at variance with entries 8 and 10 where it was added since the beginning. While CS_2 quenches catalysis when added stoichiometrically with respect to the catalyst, it minimally affects catalysis when added at 10% loading with respect to rhodium. Altogether, although not definitive, this speaks in favor of a homogeneous active species.

Preliminary studies on substrate scope

Experimentally, all reactions were carried out following the same procedure as described in the previous section for screening of conditions. All hydrogenated compounds were identified through ^{1}H NMR spectroscopy compared with pure samples of those species obtained from commercial sources, except for compounds **v**[6] and **xxx**[7], whose spectroscopic pattern was compared to literature records.

Computational details

Calculations were performed at the DFT level with the Gaussian 09 (Revision E.01) program.[8] The hybrid functional PBE0[9] was used throughout the computational study, and dispersion effects were accounted for by using Grimme's D3 parameter set with Becke–Johnson (BJ) damping.[10] Geometry optimizations were carried out without geometry constraints, using the 6-31G(d,p)[11] basis set to represent the C, H, P, B, and F atoms and the Stuttgart/Dresden Effective Core Potential and its associated basis set (SDD)[12] to describe the Rh atoms. Bulk solvent effects (dichloromethane) were included at the optimization stage with the SMD continuum model.[13] Energies were refined by means of single-point calculations with the larger 6-311+G(2d,p) basis set. The stationary points and their nature as minima or saddle points (TS) were characterized by vibrational analysis, which also produced zero-point (ZPE), enthalpy (H), entropy (S) and Gibbs energy (G) data at 298.15 K. The minima connected by a given transition state were determined by perturbing the transition states along the TS coordinate and optimizing to the nearest minimum. NBO analysis was performed with the NBO6.0 suite.[15] The EDA-NOCV calculations were carried out with the program package ADF 2020[16]using the PBE0-D3/6-31G(d,p) optimized geometries employing the BP86-D3 functional in conjunction with a triple-ζ-quality basis set using uncontracted Slater-type orbitals (STOs) augmented by two sets of polarization functions (TZ2P) with a frozen-core approximation for the core electrons.

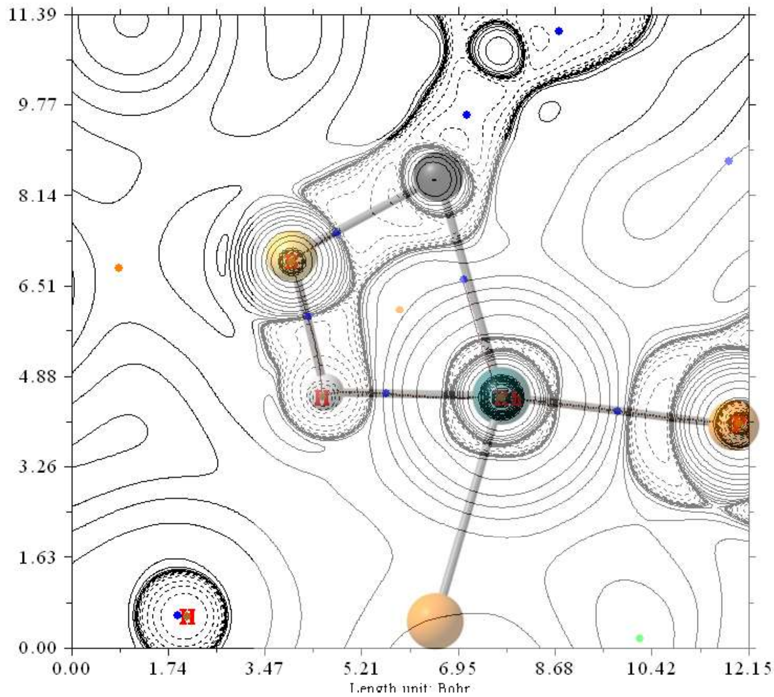

Figure S34. Laplacian of the electron density, $\nabla^2\rho$, plot of **3**, showing bond critical points (blue) and paths for the B–H, Rh–H, B–C and Rh–C bonds.

The value of the electron density at a bcp (ρ) has been associated with bond strength. The value of the laplacian of the electron density at a bcp, $\nabla^2\rho$, is negative in covalent interactions (density of charge accumulates) and positive in closed shell or ionic interactions (the density of charge is depleted). The total energy density (H) tends to be negative for covalent interactions and positive for ionic interactions. Another criterion to determine the nature of an interaction between two atoms is the ratio between the local Potential (V) and Kinetic (G) energies. A ratio $|V|/G < 1$ is found in ionic interactions whereas in covalent interactions $|V|/G > 2$ (intermediate interactions including metal-metal and metal-ligand interactions are characterized by positive values of $\nabla^2\rho$, by values of H close to zero and $1 < |V|/G < 2$).[14]

Table S3. Selected properties of the electron density at relevant bcps for **3**. ^{a}e·bohr^{-3}, [7]

Bond	ρ^a	G^b	V^b	H^b	$\|V\|/G$	$\nabla^2\rho^c$
**H−B**	1.21E-01	1.00E-01	-2.12E-01	-1.12E-01	2.12E+00	-4.79E-02
**Rh−H**	2.63E-01	4.33E-01	-8.00E-01	-3.67E-01	1.85E+00	2.62E-01

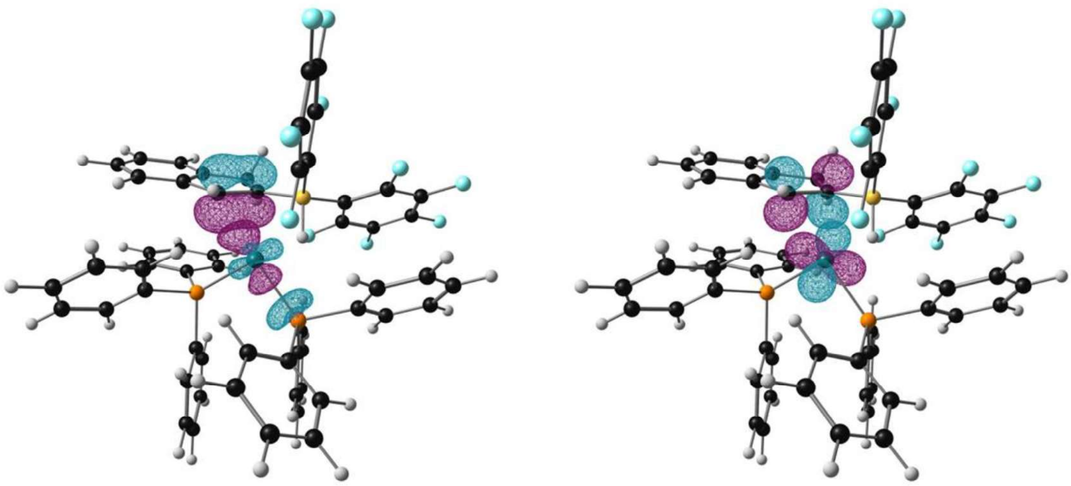

Figure S35. NBOs 137 (donor, BD C=C) and 263 (acceptor, BD* Rh−P) (left), NBOs 86 (donor, LP Rh) and 282 (acceptor, BD* C=C) of complex **3**.

Energy Decomposition Analysis for **5**.

$$\Delta E_{\text{int}} = \Delta E_{\text{elstat}} + \Delta E_{\text{Pauli}} + \Delta E_{\text{orb}} + \Delta E_{\text{disp}}$$

$$\Delta E_{\text{int}} = -88.63 \text{ kcal/mol}$$

$$\Delta E_{\text{Pauli}} = 201.36 \text{ kcal/mol}$$

$$\Delta E_{\text{elstat}} = -136.58 \text{ kcal/mol}$$

$$\Delta E_{\text{orb}} = -113.56 \text{ kcal/mol}$$

$$\Delta E_{\text{disp}} = -39.86 \text{ kcal/mol}$$

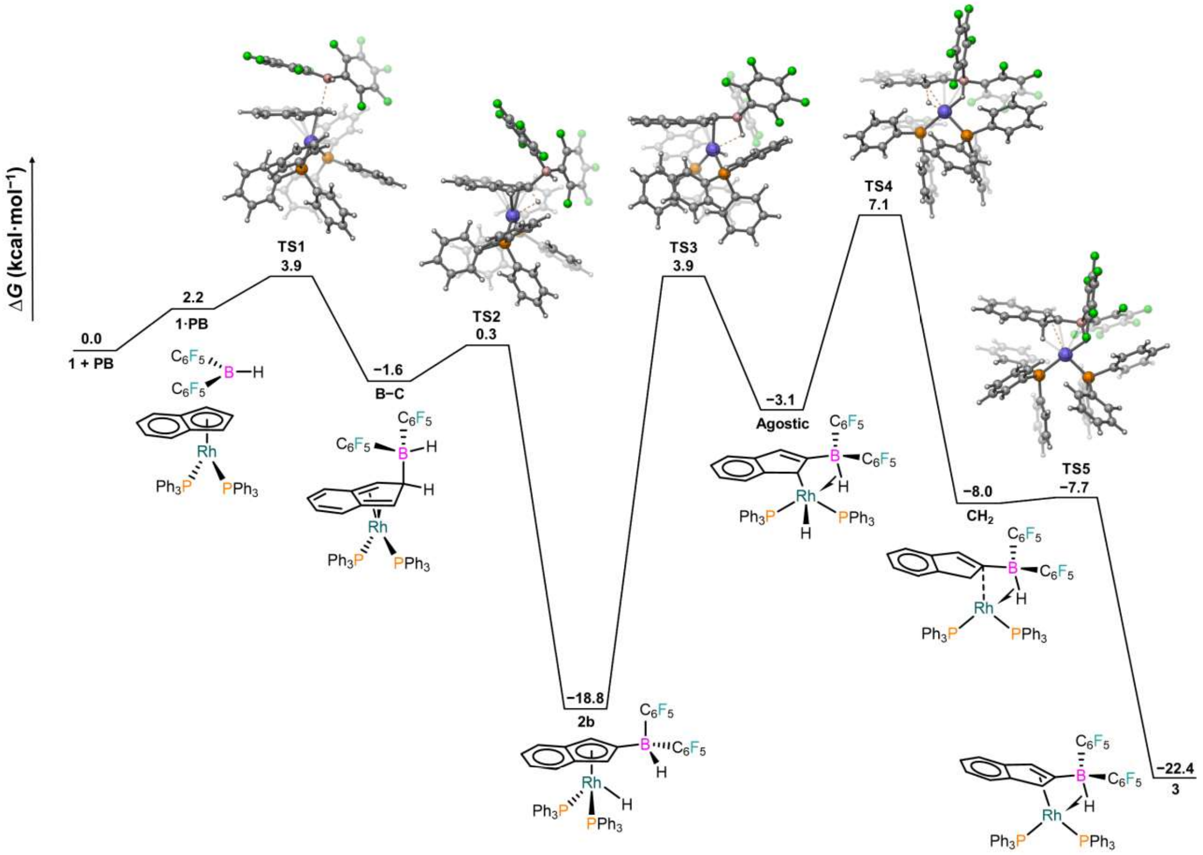

Figure S36. Free energy profile of an alternative pathway for the conversion of **1** and Piers' borane into **3** at the SMD(dichloromethane)-PBE0-D3(BJ)/SDD(Rh)/6-311+G(2d,p)//SMD(dichloromethane)-PBE0-D3(BJ)/SDD(Rh)/6-31G(d,p) level of theory.

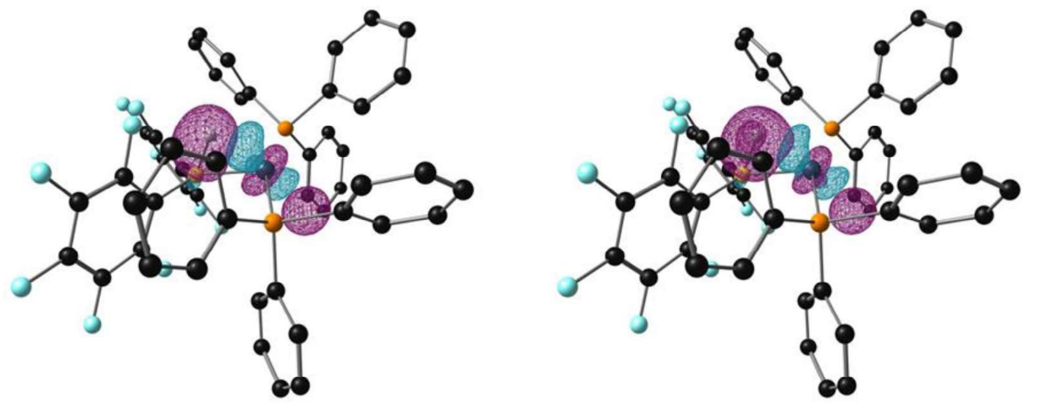

Figure S37. NBOs (left) and NLMOs (right) 209 (donor, BD B−H) and 234 (acceptor, BD* Rh−H) of complex **5**.

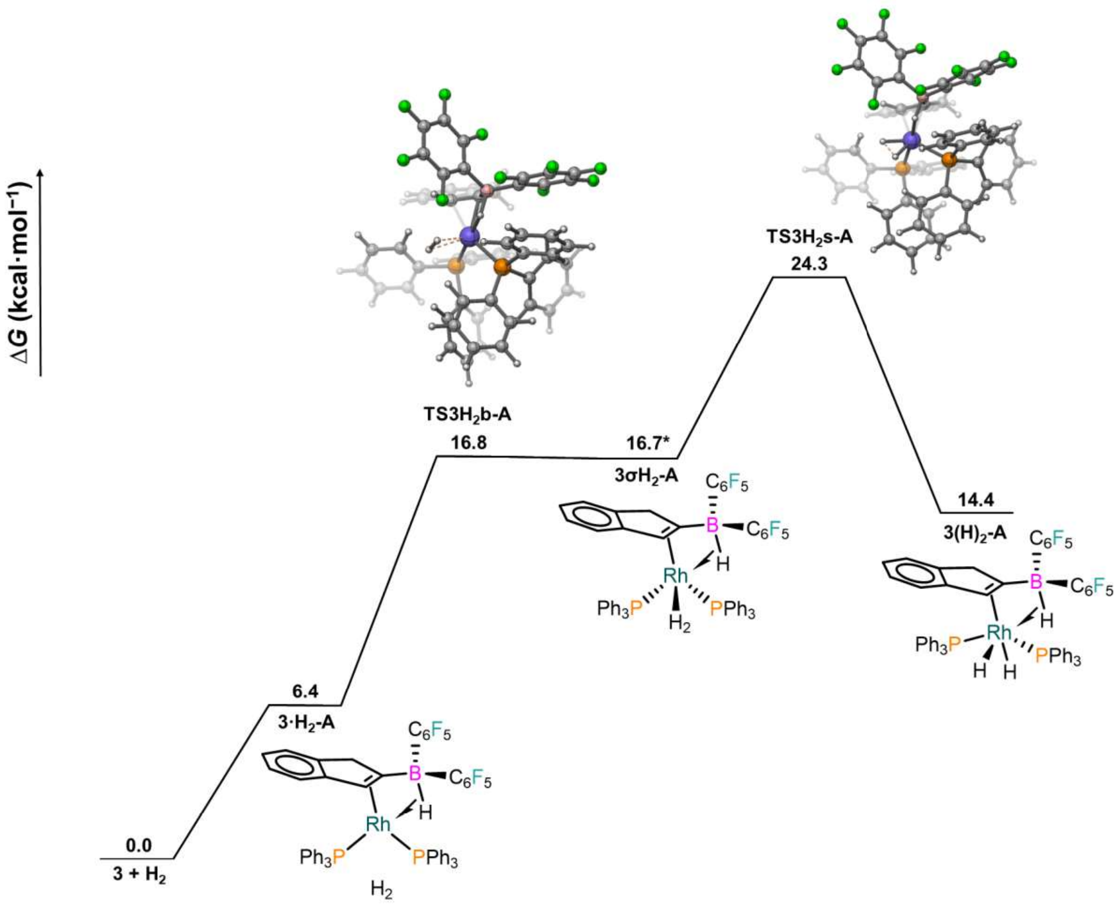

Figure S38. Free energy profile for the coordination and oxidative addition of H_2 at complex **3** at the SMD(dichloromethane)-PBE0-D3(BJ)/SDD(Rh)/6-311+G(2d,p)//SMD(dichloromethane)-PBE0-D3(BJ)/SDD(Rh)/6-31G(d,p) level of theory.

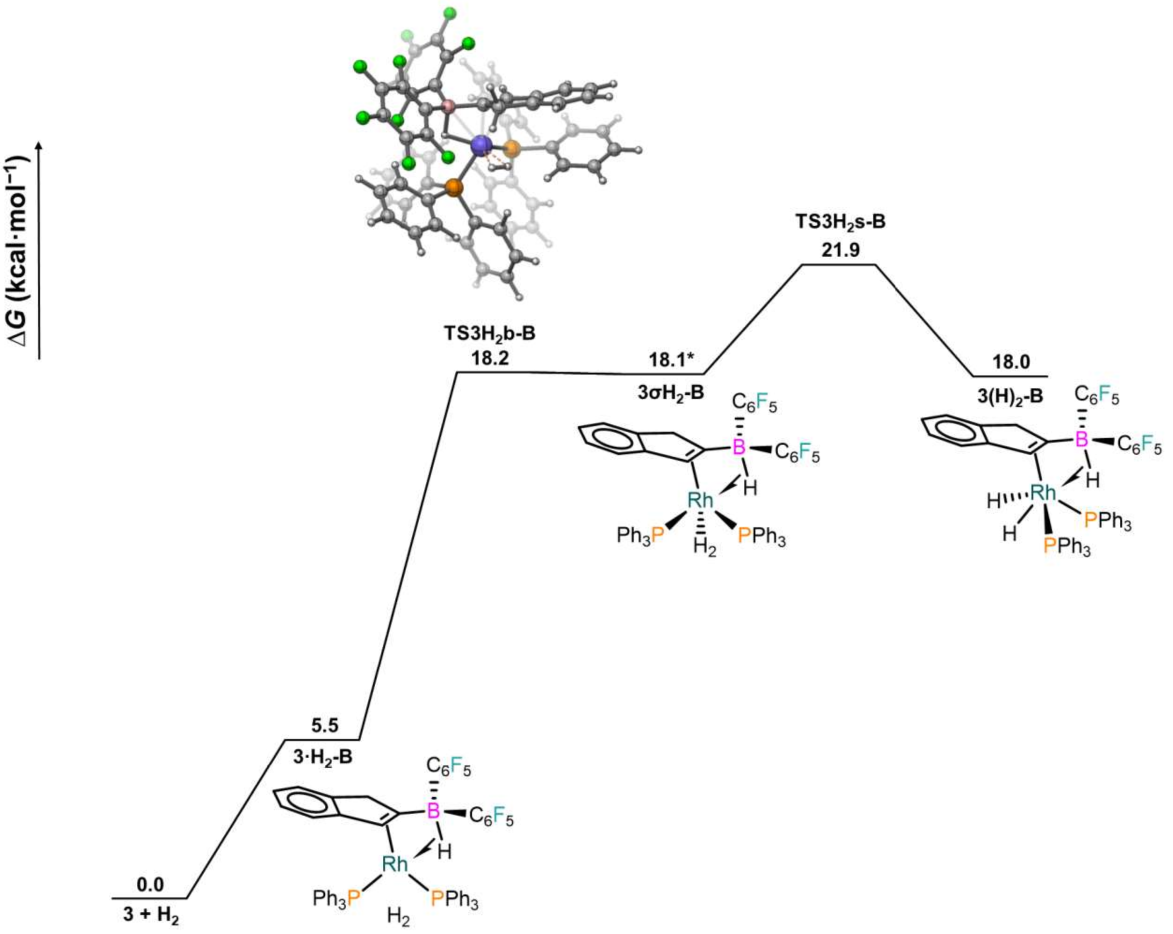

Figure S39. Alternative free energy profile for the coordination and oxidative addition of H_2 at complex **3** at the SMD(dichloromethane)-PBE0-D3(BJ)/SDD(Rh)/6-311+G(2d,p)//SMD(dichloromethane)-PBE0-D3(BJ)/SDD(Rh)/6-31G(d,p) level of theory.

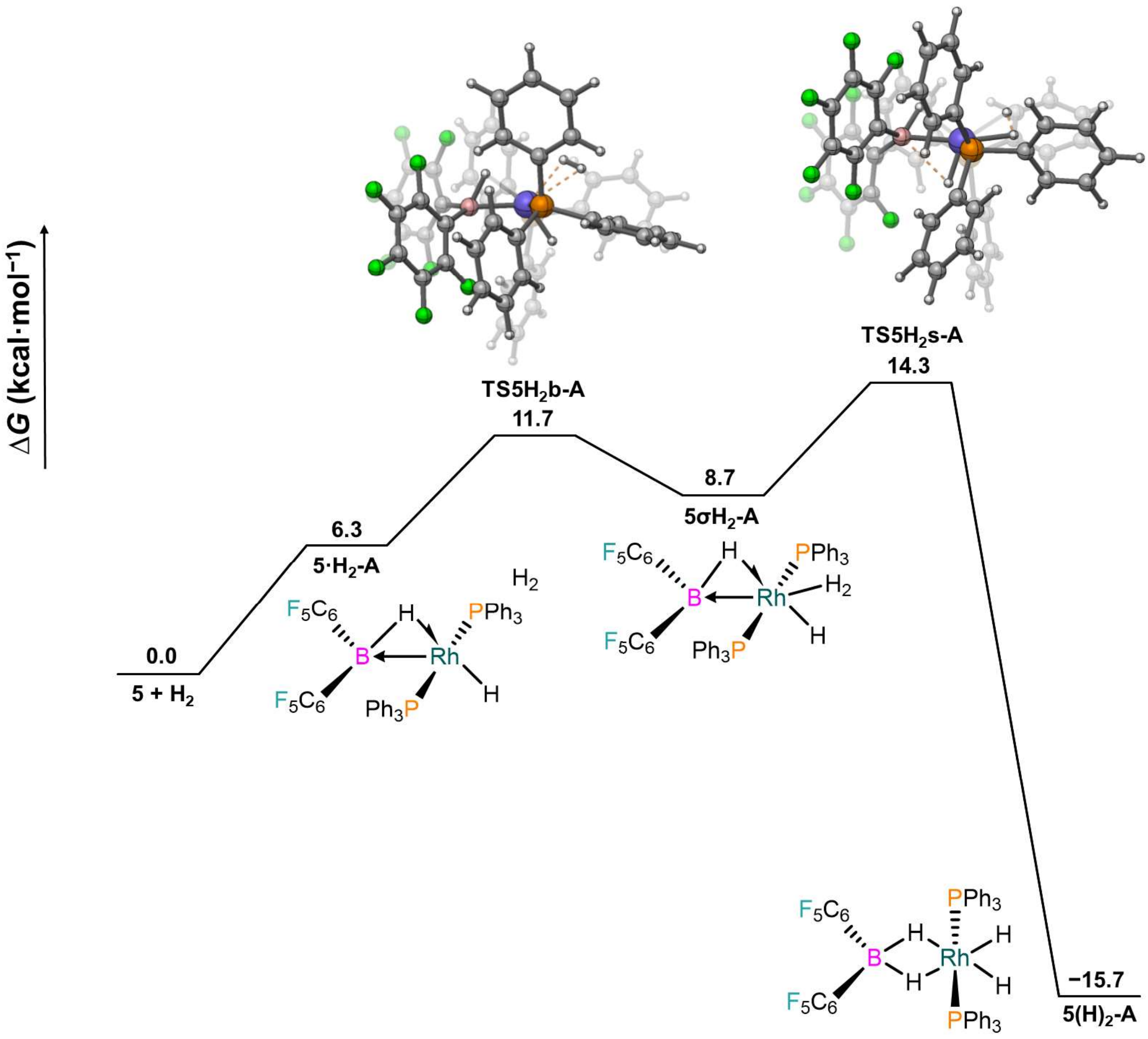

Figure S40. Free energy profile for the coordination and oxidative addition of H_2 at complex **5** at the SMD(dichloromethane)-PBE0-D3(BJ)/SDD(Rh)/6-311+G(2d,p)//SMD(dichloromethane)-PBE0-D3(BJ)/SDD(Rh)/6-31G(d,p) level of theory.

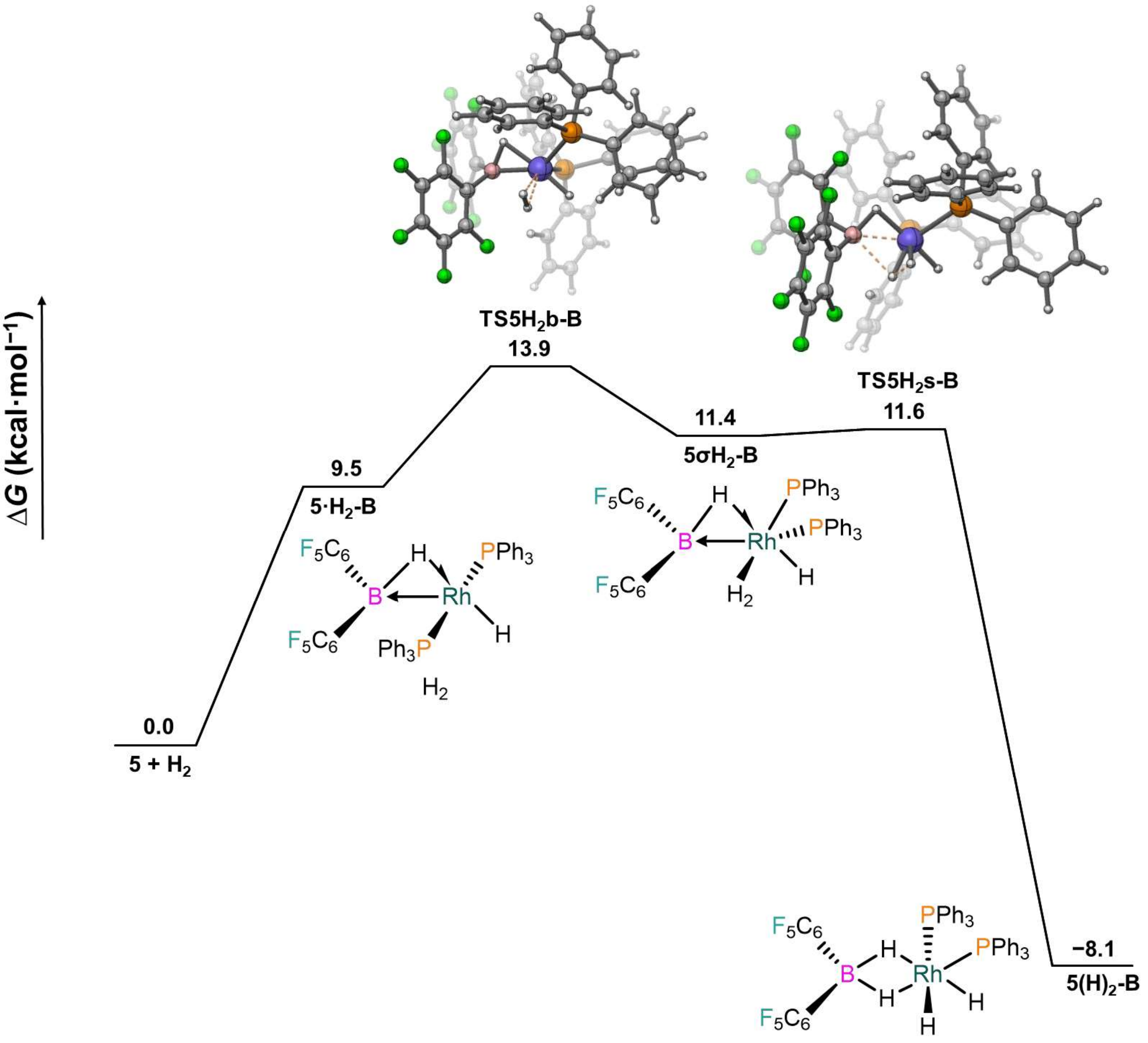

Figure S41. Alternative free energy profile for the coordination and oxidative addition of H_2 at complex **5** at the SMD(dichloromethane)-PBE0-D3(BJ)/SDD(Rh)/6-311+G(2d,p)//SMD(dichloromethane)-PBE0-D3(BJ)/SDD(Rh)/6-31G(d,p) level of theory.

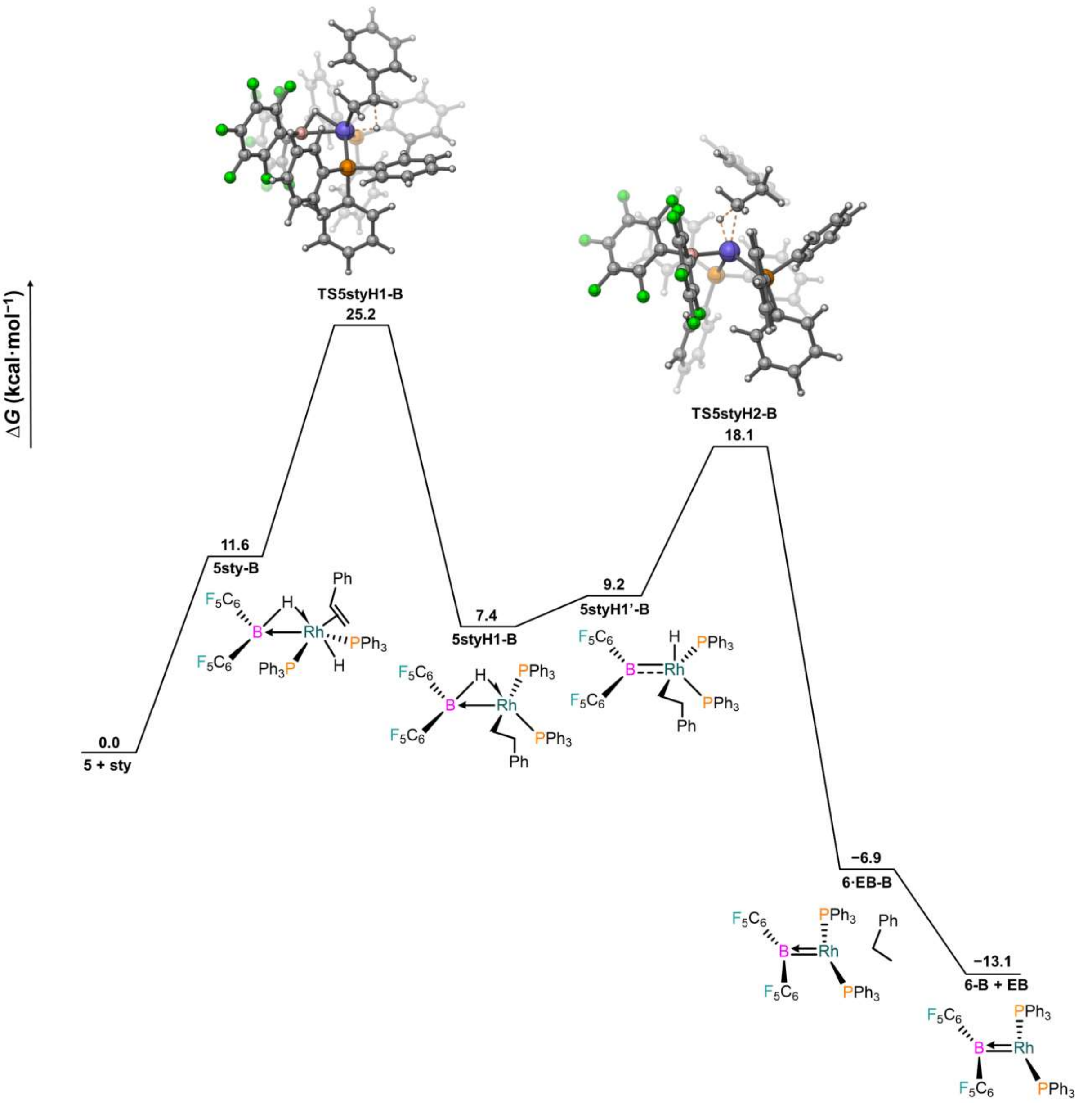

Figure S42. Alternative free energy profile for the hydrogenation of styrene at complex **5** at the SMD(dichloromethane)-PBE0-D3(BJ)/SDD(Rh)/6-311+G(2d,p)//SMD(dichloromethane)-PBE0-D3(BJ)/SDD(Rh)/6-31G(d,p) level of theory.

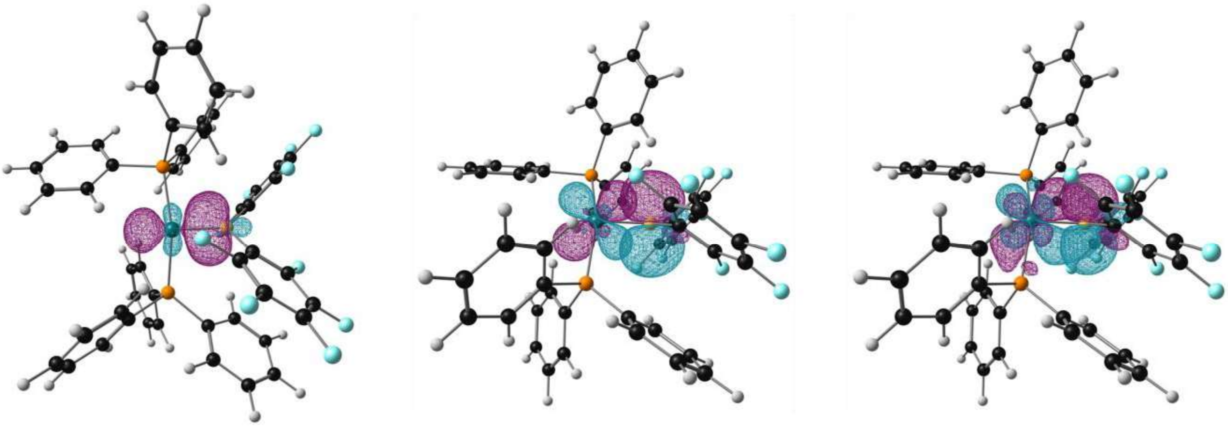

Figure S43. NBOs 110 (left, BD Rh−B) and 77 and 231 (center, donor (Rh) and acceptor (B), respectively) and NLMOs 77 and 231 (right, donor (Rh) and acceptor (B), respectively) of species **6**.

Energy Decomposition Analysis for 6.

A) Fragments: $[Rh(PPh_3)_2]^+$ and $[B(C_6F_5)_2]^-$

$$\Delta E_{\text{int}} = -212.48 \text{ kcal/mol}$$

$$\Delta E_{\text{Pauli}} = 368.85 \text{ kcal/mol}$$

$$\Delta E_{\text{elstat}} = -379.93 \text{ kcal/mol}$$

$$\Delta E_{\text{orb}} = -164.63 \text{ kcal/mol}$$

$$\Delta E_{\text{disp}} = -36.76 \text{ kcal/mol}$$

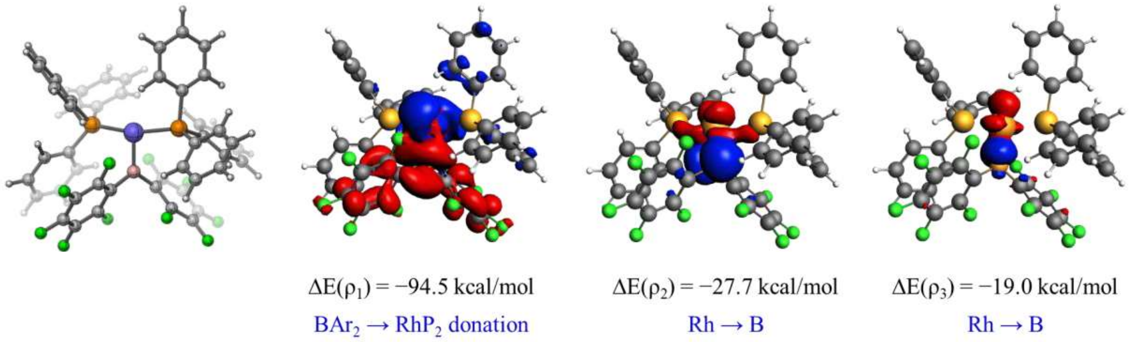

Figure S44A. DFT-optimized structure (left). Contour plots of NOCV deformation densities Δρ and associated energies ΔE(ρ) in 6, charged closed-shell fragments. Electron-density charge flows in the direction red → blue.

B) Fragments: $[Rh(PPh_3)_2]^{\cdot}$ and $[B(C_6F_5)_2]^{\cdot}$

$$\Delta E_{\text{int}} = -116.77 \text{ kcal/mol}$$

$$\Delta E_{\text{Pauli}} = 259.45 \text{ kcal/mol}$$

$$\Delta E_{\text{elstat}} = -202.97 \text{ kcal/mol}$$

$$\Delta E_{\text{orb}} = -136.49 \text{ kcal/mol}$$

$$\Delta E_{\text{disp}} = -36.76 \text{ kcal/mol}$$

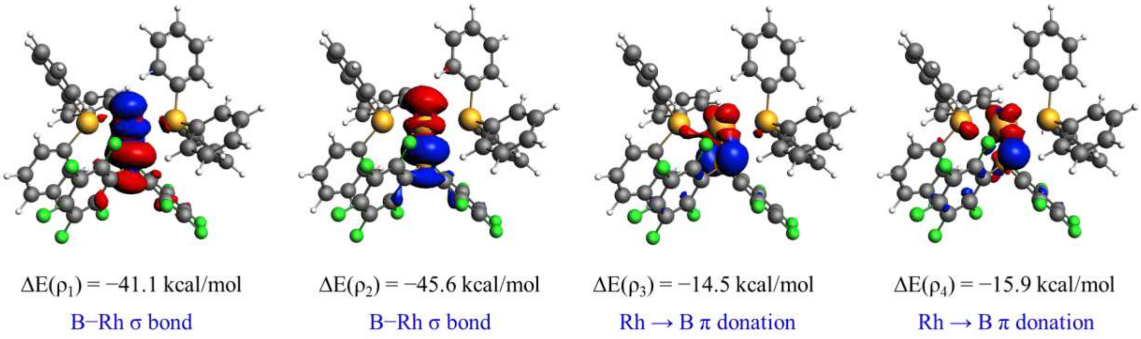

Figure S44B. DFT-optimized structure (left). Contour plots of NOCV deformation densities Δρ and associated energies ΔE(ρ) in 6, open-shell fragments. Electron-density charge flows in the direction red → blue.

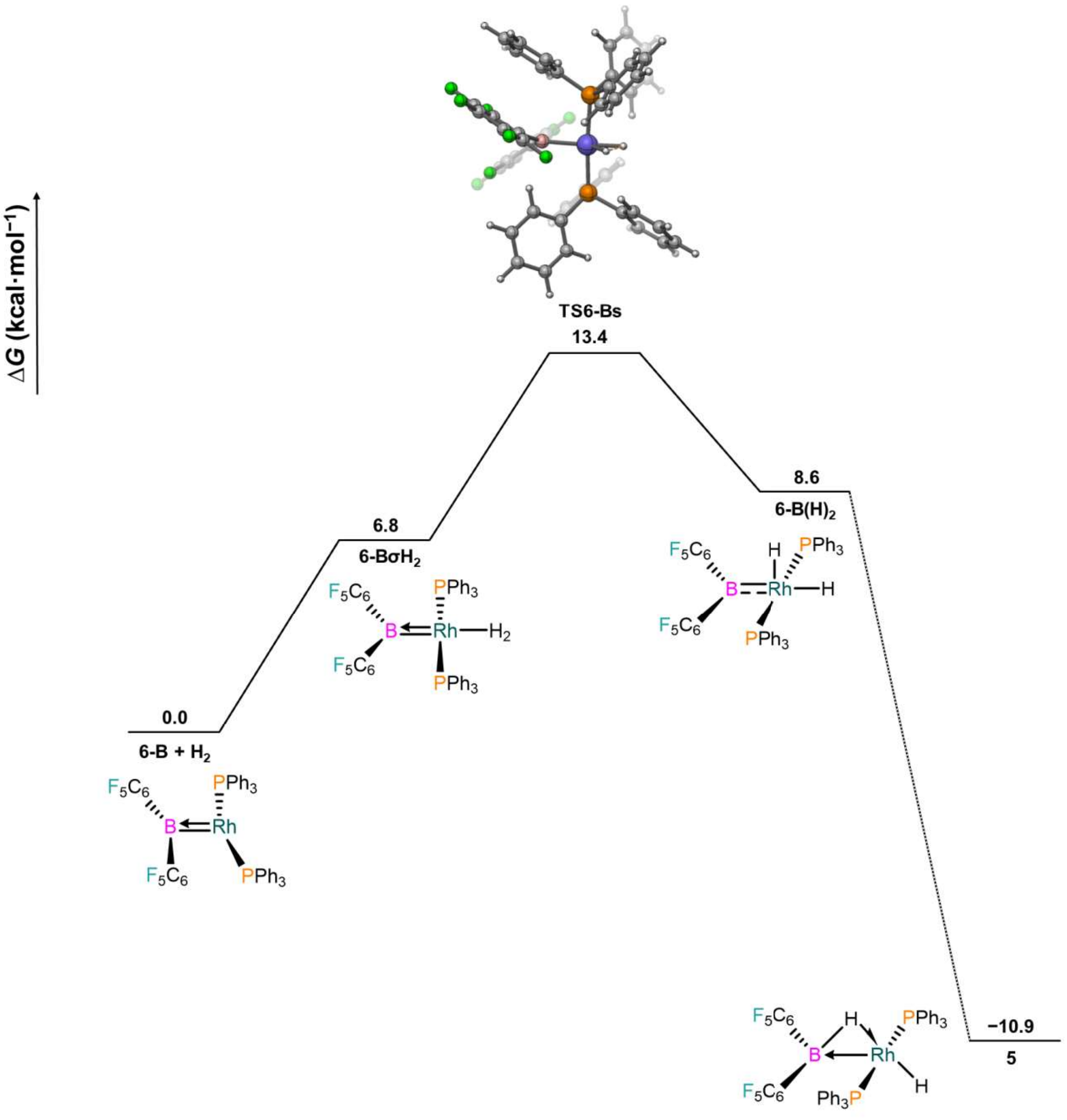

Figure S45. Free energy profile for the coordination and oxidative addition of H_2 at species **6** at the SMD(dichloromethane)-PBE0-D3(BJ)/SDD(Rh)/6-311+G(2d,p)//SMD(dichloromethane)-PBE0-D3(BJ)/SDD(Rh)/6-31G(d,p) level of theory.

References

1 a) Klingert, B.; Werner, H. Basische Metalle, XLII. Die Metall-Basizität der Komplexe $C_5Me_5Rh(PMe_3)_2$, $C_5Me_5Rh(C_2H_4PMe_3$ und $C_5Me_5Rh(C_2H_4)P_2Me_4$: Neue Pentamethyl-cyclopentadienylrhodium(I)- und -rhodium(III)-Verbindungen. *Chem. Ber.,* **1983**, *116*, 1450−1462. b) Alférez, M. G.; Moreno, J. J.; Maya, C.; Campos, J. Polarized Au(i)/Rh(i) bimetallic pairs cooperatively trigger ligand non-innocence and bond activation. *Dalton Trans*., **2023**,*52*, 3835−3845. DOI: 10.1039/D3DT00410D

2 Bentley, J. N.; Pradhan, E.; Zeng, T.; Caputo, C. B. Bis(pentafluorophenyl)phenothiazylborane – an intramolecular frustrated Lewis pair catalyst for stannane dehydrocoupling. *Dalton Trans.,* **2020**, *49*, 16054−16058.

3 Schnurr, A.; Samigullin, K.; Breunig, J. M.; Bolte, M.; Lerner H.-W.; Wagner, M. One-Pot Synthesis of $[(C_6F_5)_2BH_2]^-$ from $C_6F_5MgBr/BH_3{\cdot}SMe_2$ and Its in Situ Transformation to Piers' Borane*Organometallics*, 2011, **30**, 10, 2838−2843. DOI: 10.1021/om200186w

4 a) Bourhis, L. J., Dolomanov, O. V., Gildea, R. J., Howard, J. A. K., Puschmann, H. The anatomy of a comprehensive constrained, restrained refinement program for the modern computing environment - Olex2 dissected. *Acta Cryst*. **2015,** A71, 59−75. b) Dolomanov, O. V., Bourhis, L. J., Gildea, R. J, Howard, J. A. K.; Puschmann, H. OLEX2: A Complete Structure Solution, Refinement and Analysis Program. *J. Appl. Cryst.* **2009**, 42, 339−341. c) Sheldrick, G. M. Integrated space-group and crystal-structure determination. *Acta Cryst*. **2015**, C71, 3−8.

5 Sheldrick, G. M. Crystal structure refinement with SHELXL *Acta Cryst*. **2008,** *A64*, 112.

6 a) Royal society of chemistry home page http://www.chemspider.com/Chemical-Structure.4482215.htmla, CSID:4482215, (accessed 2022-11-10) b) Wiley spectra data base, https://spectrabase.com/compound/KKKkq1TeEDX. (accessed 2022-11-10)

7 Chen, C.; Daniliuc, C. G.; Mück-Lichtenfeld, C.; Kehr, G.; Erker, G. A rare olefin 1,1-carboboration reaction opens a synthetic pathway to an unusually structured frustrated Lewis pair. *Chem. Commun.*, **2020**, *56*, 8806−8809. DOI: 10.1039/D0CC01255F

8 Frisch, M. J.; Trucks, G. W.; Schlegel, H. B.; Scuseria, G. E.; Robb, M. A.; Cheeseman, J. R.; Scalmani, G.; Barone, V.; Mennucci, B.; Petersson, G. A.; Nakatsuji, H.; Caricato, M.; Li, X.; Hratchian, H. P.; Izmaylov, A. F.; Bloino, J.; Zheng, G.; Sonnenberg, J. L.; Hada, M.; Ehara, M.; Toyota, K.; Fukuda, R.; Hasegawa, J.; Ishida, M.; Nakajima, T.; Honda, Y.; Kitao, O.; Nakai, H.; Vreven, T.; Montgomery, J. A. J.; Peralta, J. E.; Ogliaro, F.; Bearpark, M.; Heyd, J. J.; Brothers, E.; Kudin, K. N.; Staroverov, V. N.; Kobayashi, R.; Normand, J.; Raghavachari, K.; Rendell, A.; Burant, J. C.; Iyengar, S. S.; Tomasi, J.; Cossi, M.; Rega, N.; Millam, J. M.; Klene, M.; Knox, J. E.; Cross, J. B.; Bakken,V; Adamo, C.; Jaramillo, J.; Gomperts, R.; Stratmann, R. E.; Yazyev, O.; Austin, A. J.; Cammi, R.; Pomelli, C.; Ochterski, J. W.; Martin, R. L.; Morokuma, K.; Zakrzewski, V. G.; Voth, G. A.; Salvador, P.; Dannenberg, J. J.; Dapprich, S.; Daniels, A. D.; Farkas, O.; Foresman, J. B.; Ortiz, J. V.; Cioslowski, J.; Fox, D. J. Gaussian 09, Revision E.01, Gaussian, Inc.: Wallingford CT, **2013**.

9 Adamo, C.; Barone, V. Toward reliable density functional methods without adjustable parameters: The PBE0 model. *J. Chem. Phys*. **1999**, *110*, 6158−6170.

10 Grimme, S.; Antony, J.; Ehrlich, S.; Krieg, H. A consistent and accurate ab initio parametrization of density functional dispersion correction (DFT-D) for the 94 elements H-Pu. *J. Chem. Phys*. **2010**, *132*, 154104.

11 a) Hehre, W. J.; Ditchfield, R.; Pople, J. A. Self—Consistent Molecular Orbital Methods. XII. Further Extensions of Gaussian—Type Basis Sets for Use in Molecular Orbital Studies of Organic Molecules. *J. Phys. Chem.* **1972**, *56*, 2257−2261. b) Hariharan, P. C.; Pople, J. A. The influence of polarization functions on molecular orbital hydrogenation energies. *Theor. Chim. Acta.* **1973**, *28*, 213−222. c) Francl, M. M.; Pietro, W. J.; Hehre, W. J.; Binkley, J. S.; Gordon, M. S.; Defrees, D. J.; Pople, J. A. Self-consistent molecular orbital methods. XXIII. A polarization-type basis set for second-row elements. *J. Chem. Phys.* **1982**, *77*, 3654−3665.

12 Andrae, D.; Haeussermann, U.; Dolg, M.; Stoll, H.; Preuss, H.; Energy-adjustedab initio pseudopotentials for the second and third row transition elements. *Theor. Chim. Acta* **1990**, *77*, 123−141..
13 Marenich, A. V.; Cramer, C. J.; Truhlar, D. G. Universal Solvation Model Based on Solute Electron Density and on a Continuum Model of the Solvent Defined by the Bulk Dielectric Constant and Atomic Surface Tensions. *J. Phys. Chem. B* **2009**, *113*, 6378−6396.
14 a) Varadwaj, P. R.; Cukrowski, I.; Marques, H. M.. DFT-UX3LYP Studies on the Coordination Chemistry of Ni^{2+}. Part 1: Six Coordinate $[Ni(NH_3)_n(H_2O)_{6-n}]^{2+}$ Complexes. *J. Phys. Chem. A* **2008**, *112*, 10657−10666; b) Varadwaj, P. R.; Varadwaj, A.; Marques, H. M. DFT-B3LYP, NPA-, and QTAIM-Based Study of the Physical Properties of $[M(II)(H_2O)_2(15\text{-crown-}5)]$ (M = Mn, Fe, Co, Ni, Cu, Zn) Complexes. *J. Phys. Chem. A* **2011**, *115*, 5592−5601; c) Curado, N.; Carrasco, M.; Álvarez, E.; Maya, C.; Peloso, R.; Rodríguez, A.; López-Serrano, J.; Carmona, E. Lithium Di- and Trimethyl Dimolybdenum(II) Complexes with Mo–Mo Quadruple Bonds and Bridging Methyl Groups. *J. Am. Chem. Soc.* **2015**, *137*, 12378−12387.

15) NBO 6.0. E. D. Glendening, J. K. Badenhoop, A. E. Reed, J. E. Carpenter, J. A. Bohmann, C. M. Morales, C. R. Landis, and F. Weinhold (Theoretical Chemistry Institute, University of Wisconsin, Madison, WI, **2013**); http://nbo6.chem.wisc.edu/

16) ADF program: www.scm.com
